# C-2 Thiophenyl Tryptophan Trimers Inhibit Cellular Entry of SARS-CoV-2 through Interaction with the Viral Spike (S) Protein

**DOI:** 10.1021/acs.jmedchem.3c00576

**Published:** 2023-07-20

**Authors:** Marta Gargantilla, Clara Francés, Anmol Adhav, Alicia Forcada-Nadal, Belén Martínez-Gualda, Olaia Martí-Marí, María Luisa López-Redondo, Roberto Melero, Clara Marco-Marín, Nadine Gougeard, Carolina Espinosa, Antonio Rubio-del-Campo, Rafael Ruiz-Partida, María del Pilar Hernández-Sierra, Laura Villamayor-Belinchón, Jerónimo Bravo, José-Luis Llacer, Alberto Marina, Vicente Rubio, Ana San-Félix, Ron Geller, María-Jesús Pérez-Pérez

**Affiliations:** † Instituto de Química Médica (IQM, CSIC), c/Juan de la Cierva 3, Madrid 28006, Spain; ‡ Institute for Integrative Systems Biology (I2SysBio), UV-CSIC, c/Catedrático Agustin Escardino, 9, Paterna 46980, Valencia, Spain; § Instituto de Biomedicina de Valencia (IBV, CSIC), c/Jaime Roig 11, Valencia 46010, Spain; ∥ Group 739, Centro de Investigación Biomédica en Red en Enfermedades Raras (CIBERER-ISCIII), Madrid 28049, Spain; ⊥ Centro Nacional de Biotecnología (CNB, CSIC), c/Darwin 3, Madrid 28049, Spain

## Abstract

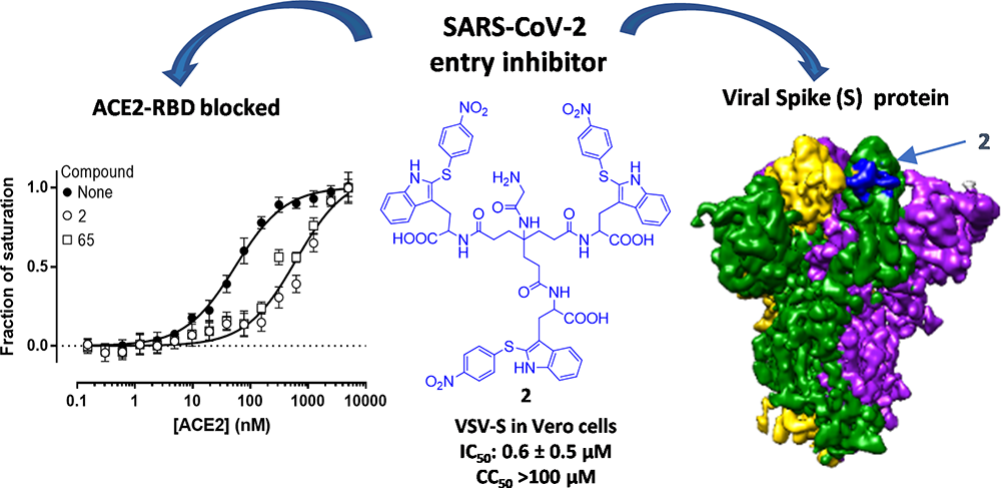

Severe acute respiratory syndrome coronavirus 2 (SARS-CoV-2) causes COVID-19, by infecting cells via the interaction of its spike protein (S) with the primary cell receptor angiotensin-converting enzyme (ACE2). To search for inhibitors of this key step in viral infection, we screened an in-house library of multivalent tryptophan derivatives. Using VSV-S pseudoparticles, we identified compound **2** as a potent entry inhibitor lacking cellular toxicity. Chemical optimization of **2** rendered compounds **63** and **65**, which also potently inhibited genuine SARS-CoV-2 cell entry. Thermofluor and microscale thermophoresis studies revealed their binding to S and to its isolated receptor binding domain (RBD), interfering with the interaction with ACE2. High-resolution cryoelectron microscopy structure of S, free or bound to **2**, shed light on cell entry inhibition mechanisms by these compounds. Overall, this work identifies and characterizes a new class of SARS-CoV-2 entry inhibitors with clear potential for preventing and/or fighting COVID-19.

## Introduction

**Figure 1:**
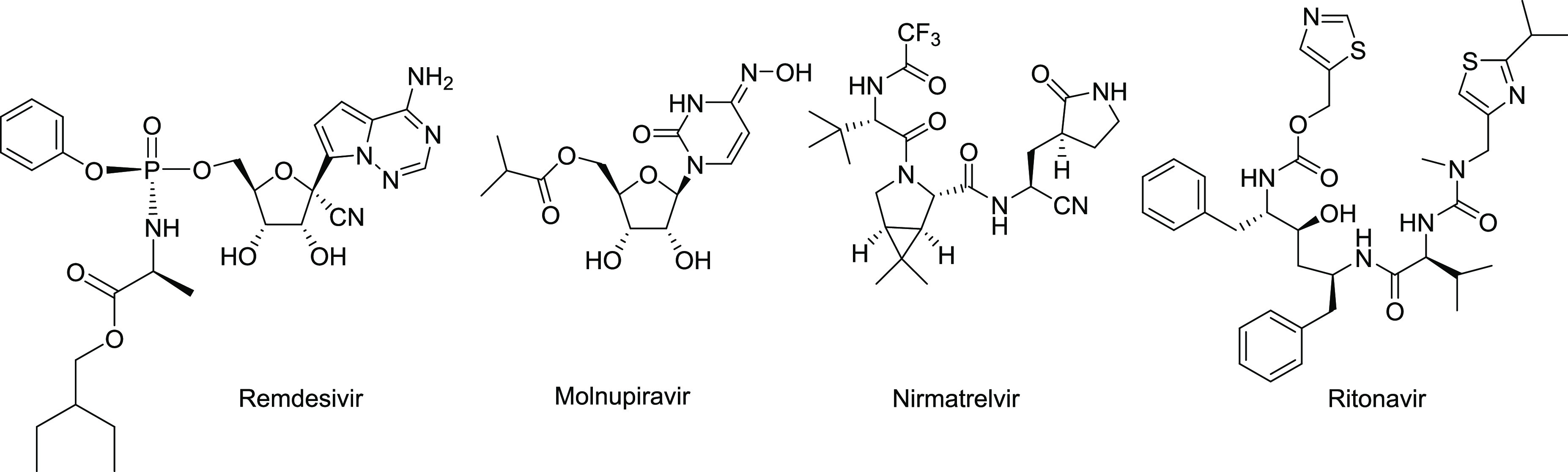
Antivirals approved for emergency use against SARS-CoV-2.

The severe acute respiratory syndrome coronavirus 2 (SARS-CoV-2), discovered in December 2019 as a new betacoranovirus,^1^ is responsible of one of the largest pandemics the world has suffered in recent years, coronavirus disease 19 (COVID-19).^2^ Prior to the emergence of SARS-CoV-2, six different coronaviruses were known to infect humans, most of which caused mild respiratory diseases.^3^ The exceptions were two zoonotic betacoronaviruses, severe acute respiratory syndrome coronavirus (SARS-CoV) and Middle East respiratory syndrome coronavirus (MERS-CoV), that caused high mortality in relevant epidemic outbreaks.^3,4^ Unfortunately, no antiviral drugs against coronavirus infections were available at the time COVID-19 emerged, leaving the world defenseless against this new disease. In the search for nonbiological antivirals, special emphasis was placed on drug repurposing to accelerate the clinical implementation of effective drugs. As a result, remdesivir, a prodrug that targets viral RNA synthesis and that was initially developed against Ebola and Marburg viruses,^5^ was approved for treatment of hospitalized COVID patients (Figure 1).^6^ More recently, two orally available drugs, molnupiravir, a prodrug of 4-hydroxycytidine,^7^ and a combination of nirmatrelvir and ritonavir, named Paxlovid,^8^ have received emergency authorization (Figure 1). Despite these advances, concerns about the potential side effects of these drugs (particularly of molnupiravir), their interference with other drugs (in the case of Paxlovid), the potential appearance of resistant viral strains, and/or the possibility of insufficient therapeutic efficacy warrant the search for novel antiviral drug candidates.

The early stages of viral infection, namely, attachment to host cells and entry, represent attractive targets for antiviral therapy as their inhibition can block infection prior to the exponential growth phase of the virus.^9^ In the case of coronaviruses, the spike protein (S), which protrudes from the viral envelope and gives viruses of this family their characteristic appearance, plays a crucial role in mediating viral entry and represents a key target for recognition by the host immune system.^10,11^ In SARS-CoV-2, S is a transmembrane glycoprotein that forms homotrimers on the viral membrane and that is cleaved during virus maturation into two subunits, S1 and S2, that remain associated in the viral membrane-bound S trimer.^9,12^ The receptor binding domain (RBD), located within S1, mediates the binding to the primary host receptor, the human angiotensin converting enzyme 2 (hACE2), while membrane fusion and viral entry are driven by the S2 domain.^12^ Antibodies targeting the S protein are able to neutralize the virus, and several monoclonal antibodies have been used for COVID19 treatment, although reduced efficacy has been observed against new variants.^13,14^ Moreover, antibody-based therapies are costly,^15^ prohibiting their application in resource-poor settings. Hence, the use of compounds that can block viral entry represents an attractive antiviral strategy that could synergize with approved therapies to increase therapeutic efficacy, reduce the probability of drug-resistance, and cut down treatment costs.

Prior work from our group has shown multivalent functionalized tryptophan (Trp) derivatives to be potent inhibitors of different viruses, including human immunodeficiency virus (HIV), enterovirus 71 (EV-A71), and flavivirus infections, and to show low cytotoxicity.^16−22^ Mechanistic studies demonstrated that these compounds interact with key elements of the viral surface (glycoprotein gp120 of HIV,^19^ 5-fold axis of the EV-A71 capsid^23^ and domain III of the viral envelope glycoprotein dengue 2 virus^22^) preventing virus attachment to the host cell membranes. In previous studies, the existence of carboxylates at the Trps was shown to be critical for antiviral activity. In addition, multivalency was proven to play an important role in the antiviral activity of this class of compounds, in which multiple tryptophan residues are exposed in such a way that their indole side chain and carboxylate groups map toward the periphery of the small dendrimer. It should be mentioned that there are other initiatives to target viral entry that also rely on multivalent inhibitors.^24,25^

In this work, we screened our in-house Trp multivalent compounds for inhibition of SARS-CoV-2 entry using a high-throughput screening (HTS) assay based on pseudotyped vesicular stomatitis virus expressing the S protein of SARS-CoV-2 (VSV-S). The chemical space surrounding the top hit (defined as the compound of low cytotoxicity exhibiting the strongest antiviral activity) was explored via the synthesis of new analogues. These were screened in the VSV-S assay to identify improved compounds and validated against genuine SARS-CoV-2. Mechanistic studies confirmed that these compounds block viral entry. Thermofluor and microscale thermophoresis with pure proteins proved the binding of these compounds to the RBD in the spike of SARS-CoV-2 and corroborated their capacity to interfere with the binding of these proteins to hACE2. Finally, the structure of SARS-CoV-2 S bound to one the best inhibitors was obtained at near atomic resolution using cryoelectron microscopy (cryoEM), providing plausible structural mechanisms for the observed cellular entry-blocking antiviral activity.

## Results and Discussion

### High-Throughput Screening (HTS) Assay for Identification of SARS-CoV-2 Entry Inhibitors

**Figure 2:**
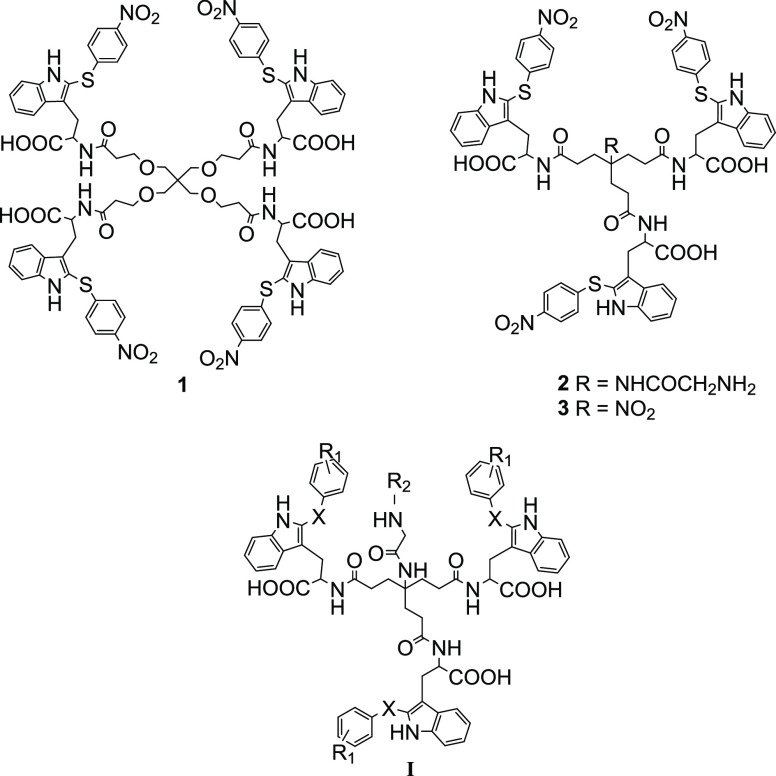
Trp derivatives **1**–**3**, which were initially identified as having antiviral activity and low cytotoxicity in the VSV-S HTS assay, while **I** represents the general structure of the compounds here synthesized and tested.

To assess whether our multivalent Trp derivatives could inhibit SARS-CoV-2, we implemented a HTS assay in Vero cells based on a pseudotyped VSV. Briefly, VSV lacking its own glycoprotein and encoding both GFP and luciferase was produced in cells that are engineered to express the ancestral Wuhan-Hu-1 SARS-CoV-2 S protein (VSV-S).^26^ In the process of budding from the cell, VSV is coated with the S protein. This enables the viral particles to employ the S protein to enter cells via interaction with the ACE2 receptor in an analogous manner to SARS-CoV-2, and infection can be monitored by quantification of GFP fluorescence. Using this system, 50 multivalent Trp derivatives were tested at an initial concentration of 20 μM (data not shown). From this primary screening, tetramer **1** and trimer **2** (Figure 2) showed significant antiviral activity in the absence of cytotoxicity (Figure S1). Both compounds share a 4-NO_2_-thiophenyl ring attached to the C-2 position of the indole ring of each Trp residue. Thus, we screened related Trp derivatives with this functionalization and also identified compound **3** as being effective (Figure 2 and Figure S1).

We next compared the antiviral activity and cytotoxicity of compounds **1**–**3** in the VSV-S assay in Vero cells. While the three compounds showed low cytotoxicity [concentration resulting in 50% cell death (CC_50_) > 100 μM], the concentration that reduced virus infection by 50% (IC_50_) was far better for trimer **2** (0.64 ± 0.47 μM) than for tetramer **1** and trimer **3** (respective IC_50_ values, 21.43 ± 9.45 and 32.85 ± 4.48 μM). Thus, compound **2** with a Gly linked to the NH_2_ at the central quaternary carbon (the focal point) was identified as a suitable hit and prompted us to synthesize trimers of general formula **I** (Figure 2) bearing *S*-phenyl rings with different substituents (R_1_) at the C-2 position of the indole ring for antiviral evaluation. Alternatives to the thioether (X) were also explored to attach the extra phenyl ring to the C-2 position of the indole. Moreover, the introduction of different chains (R_2_) at the focal point was investigated with the aim of modifying the physicochemical properties of the compounds (i.e., lipophilicity).

### Synthesis

**Scheme 1:**
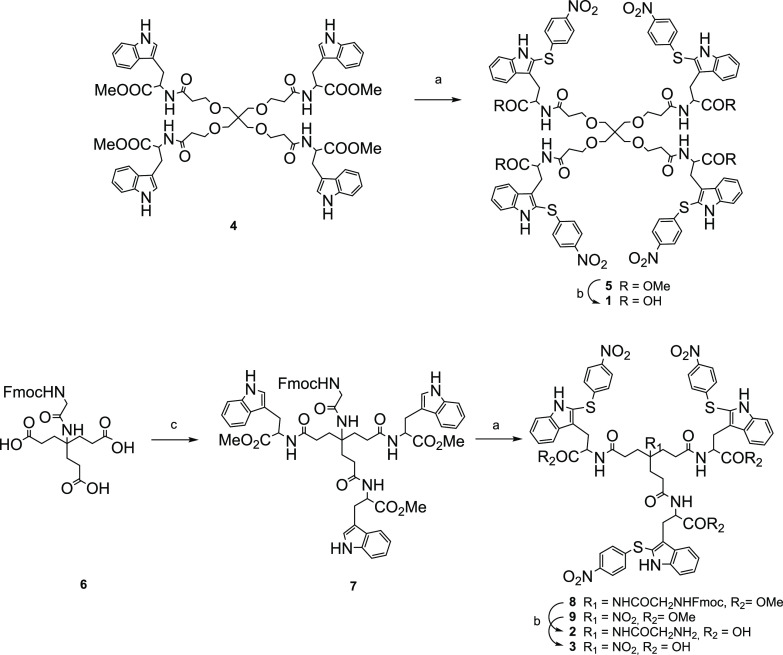
Reagents and Conditions: (a) *p*NPS-Cl, AcOH, THF, rt, 1 h, 50–88% Yields; (b) LiOH·H_2_O, THF/H_2_O, rt, 24 h, 90–95% Yields; (c) H-TrpOMe·HCl, HATU, DIPEA, Anhydrous DMF, 30 °C, 24 h, 93% Yield

Although compounds **1**–**3** were part of our in-house collection, their synthesis has not been previously disclosed. The three compounds were synthesized using the divergent approach shown in Scheme 1, involving the reaction of a multivalent methyl-protected tryptophan derivative with *p*-nitro-benzenesulfenyl chloride (*p*NPS-Cl) under acidic conditions, so that sulfenylation occurs selectively at the C-2 position of each indole ring. The synthesis of the tetrapodal derivative **1** was accomplished by reaction of the tetramer **4**(21) with *p*NPS-Cl in the presence of acetic acid to afford intermediate **5** (71%). The subsequent saponification of the ester moieties (LiOH·H_2_O) gave the desired final compound **1** in 92% yield. In the case of trimer **2**, the tripodal scaffold **7** was first prepared by reaction of the triacid **6**(27) with OMe-protected Trp (H-TrpOMe·HCl) in the presence of HATU, as the coupling reagent, and *N*,*N*-diisopropylethylamine (DIPEA) as the base (Scheme 1). Then, trimer **7** was reacted with *p*NPS-Cl in the presence of acetic acid to afford the C-2 sulfenylated derivative **8**. Subsequent saponification of the protecting ester moieties using LiOH·H_2_O, accompanied with the simultaneous removal of the Fmoc group, afforded the trimer **2** in 90% yield. The trimer **3** was obtained in 95% yield from the OMe-protected intermediate **9**, previously described by our group^22^ using a similar methyl ester saponification.

**Scheme 2:**
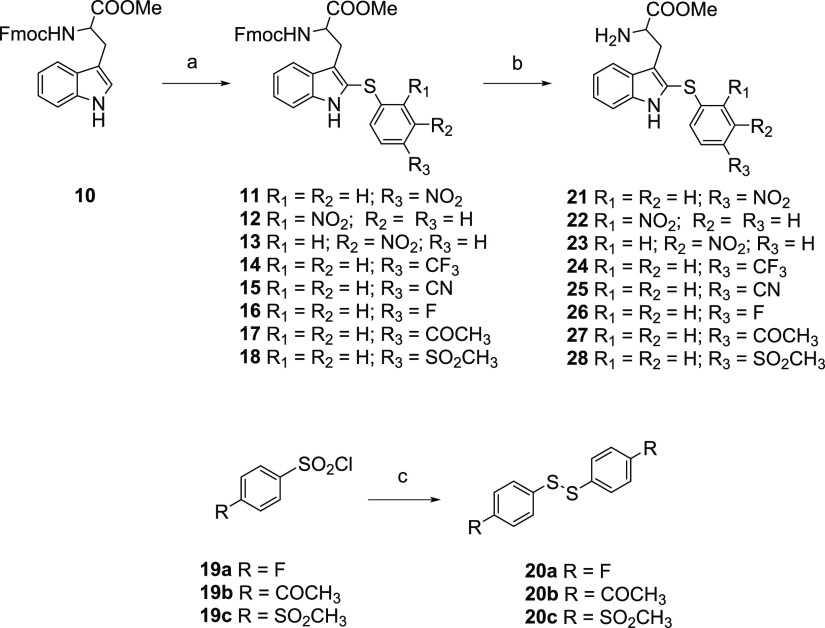
Reagents and Conditions: (a) the Corresponding Phenyl Disulfide, I_2_, MeCN, Sealed Tube, 60 °C, 4 h, 35–83% Yields; (b) Piperidine, DCM, rt, 2 h, 68–89% Yields; (c) TBAI, Anhydrous DMF, rt, 24 h, 22–26% Yields

To synthesize compounds of general formula **I** following the synthetic strategy described in Scheme 1, a great variety of sulfenyl chlorides were needed. However, these chlorides are unstable and/or not commercially available. These limitations prompted us to explore a new synthetic strategy. Among the procedures described for the sulfenylation of indoles, a particularly appealing strategy was the “one-pot” tetrabutylammonium iodide (TBAI)-mediated sulfenylation using sulfonyl chlorides.^28^ This procedure employs metal-free conditions and is compatible with benzenesulfonyl chlorides functionalized with electron-donating and electron-withdrawing groups at position 2, 3, or 4 of the phenyl ring.^28^ However, reaction of the trimer **7** with 4-NO_2_PhSO_2_Cl in the presence of TBAI in DMF failed to yield the expected trimer **8**. This unsuccessful result prompted us to study these reaction conditions on a simpler substrate, FmocTrp(OMe)^29^ (**10**) (Scheme 2). The election of Fmoc as a protecting group of the Trp was based on its stability under the acidic conditions generated in the sulfenylation reaction (due to the presence of IH) and its easy removal under basic conditions. However, reaction of **10** with 4-NO_2_PhSO_2_Cl also failed to provide the C-2 sulfenylated derivative **11**, with most of the starting material remaining unaltered. According to the mechanism proposed by He et al.,^28^ the reaction of TBAI with the sulfonyl chlorides in DMF generates the corresponding disulfides together with I_2_, so that these are indeed the reactive species, as also reported by other groups.^30,31^ Thus, we performed the reaction of FmocTrp(OMe) (**10**) with 4-NO_2_-diphenyl disulfide in the presence of I_2_ in acetonitrile at 60 °C for 4 h. In this way, the C-2 sulfenylated Trp compound **11** was obtained in 75% yield (Scheme 2).

Using this approach, different C-2 sulfenylated derivatives were synthesized (**12**–**18**), including those with a NO_2_ group at positions 2 or 3 of the thiophenyl ring (compounds **12** and **13**), or with other functional groups at position 4 (CF_3_, CN, F, COCH_3_, and SO_2_CH_3_, **14**–**18**, respectively), with yields varying from 35 to 83%. The diphenyl disulfides required to obtain the intermediates **11**–**18** were commercially available in most cases, except for a few (R_1_ = R_2_ = H; R_3_ = F, COCH_3_ or SO_2_CH_3_), which were synthesized from the corresponding benzenesulfonyl chlorides (**19a**–**c**) by reaction with TBAI in DMF at room temperature, as described for similar analogues^32^ (for details see the Supporting Information). In our hands, this procedure to obtain these disulfides (**20a**–**c**) was simpler than previously described alternative methods.^33,34^ Finally, selective Fmoc deprotection of the Trp monomers **11**–**18** using piperidine at rt yielded the intermediates **21**–**28** in good-to-high yields (68–89% yield) (Scheme 2).

**Scheme 3:**
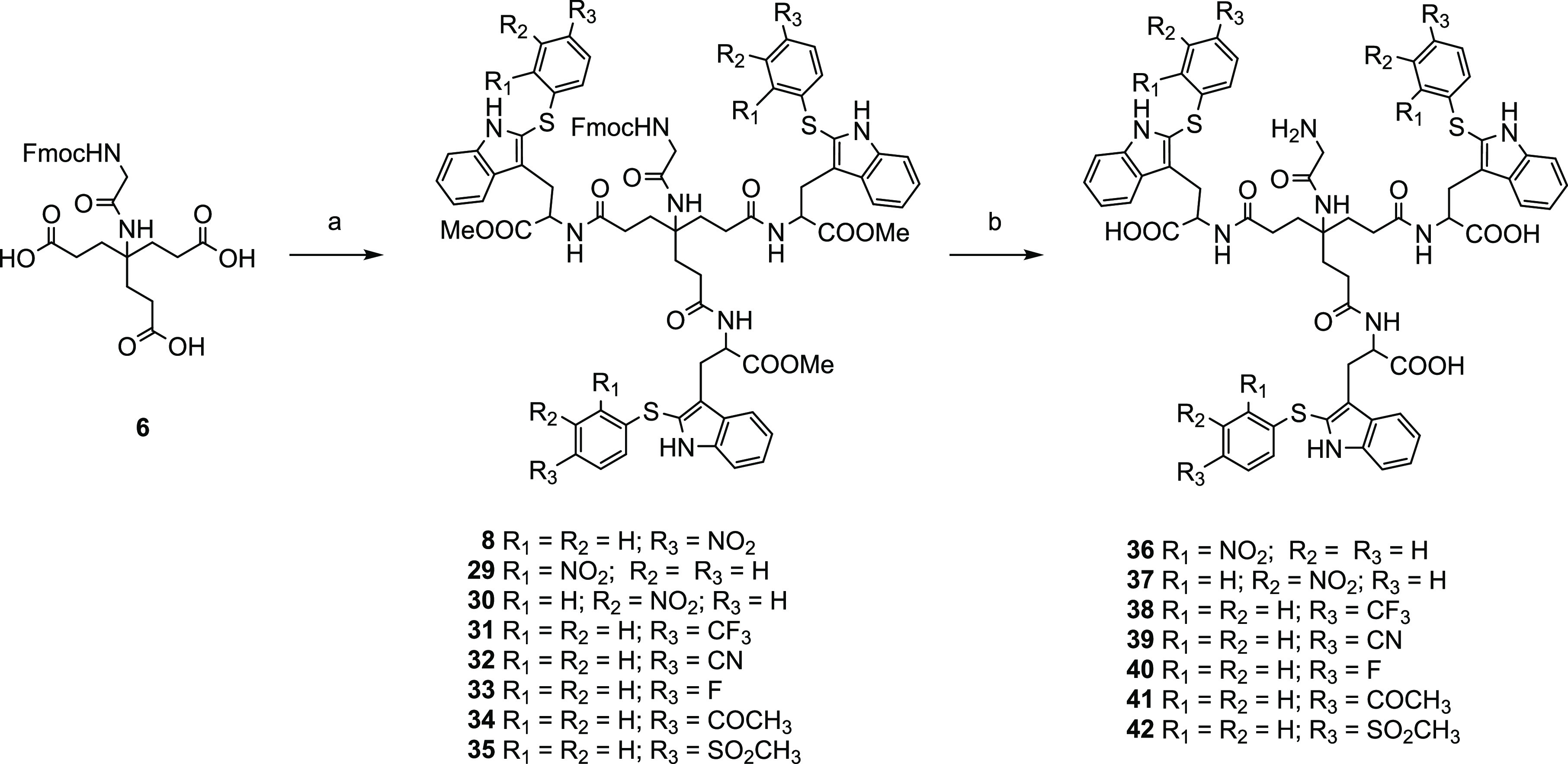
Reagents and Conditions: (a) HATU, DIPEA, Anhydrous DMF, 30 °C, 24 h, 52–89% Yields; (b) LiOH·H_2_O, THF/H_2_O, rt, 24 h, 50%-Quantitative Yields

Coupling of three units of the key intermediate (**21**–**28**) with the carboxylate groups of the Fmoc-protected glycine scaffold **6**,^27^ in the presence of HATU as a coupling reagent and DIPEA as a base, afforded the OMe trimers **8** and **29**–**35** (52–89% yield) (Scheme 3). Treatment of **29**–**35** with LiOH·H_2_O resulted in the saponification of the ester moieties with concomitant removal of the Fmoc protecting group of Gly. After acidification to pH 2 and washing, the deprotected trimers **36**–**42** were obtained (50% to quantitative yields) (Scheme 3).

**Scheme 4:**
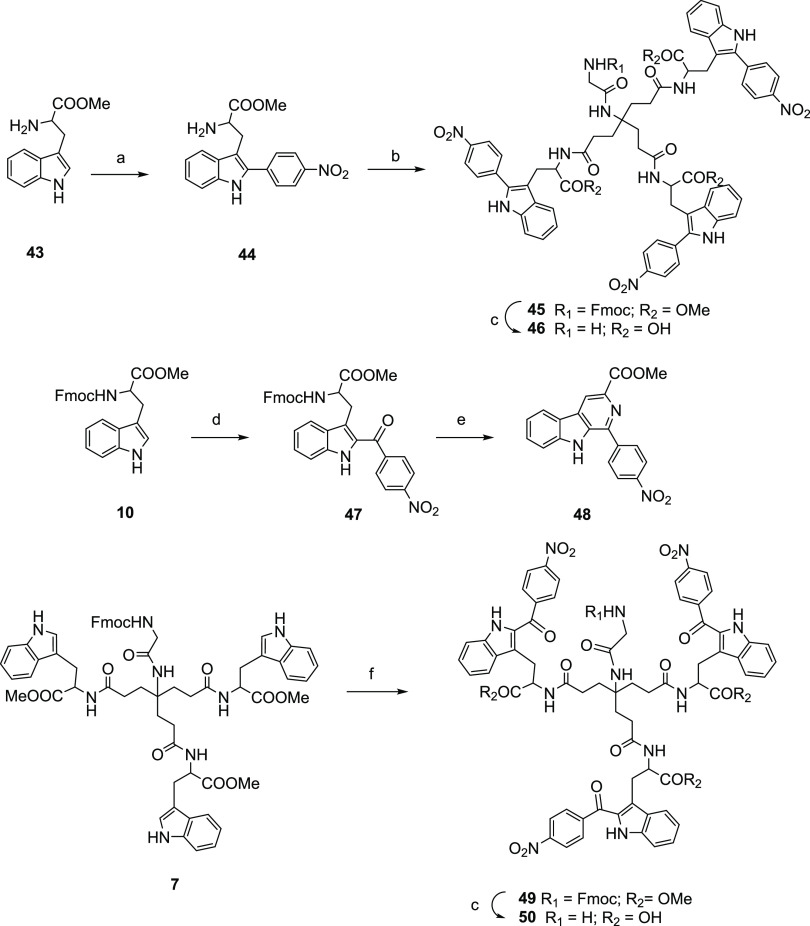
Reagents and Conditions: (a) Pd(OAc)_2_, TFA, Anhydrous DMF, MW: 100 °C, 2 h, 46% Yield; (b) **6**, HATU, DIPEA, Anhydrous DMF, 30 °C, 40 h, 99% Yield; (c) LiOH·H_2_O, THF/H_2_O, rt, 24 h, 51–96% Yields; (d) 4-Nitrobenzoyl Chloride (1.5 equiv), SnCl_4_ (3 equiv), Anhydrous DCM, 0 °C, 3 h, 37% Yield; (e) Piperidine, DCM, rt, 2 h, 53% Yield; (f) 4-Nitrobenzoyl Chloride (4.5 equiv), SnCl_4_ (9 equiv), Anhydrous DCM, 0 °C to rt, 8.5 h, 21% Yield

Alternatives to the thioether used to link the 4-NO_2_-phenyl ring and C-2 position of the indole were also explored. A direct C–C bond was assayed through a metal-catalyzed (Pd II) cross-coupling reaction (Scheme 4). For this, a mixture containing H-Trp OMe (**43**) and 1-iodo-4-nitrobenzene in DMF was MW-irradiated at 120 °C for 30 min in the presence of 5 mol % Pd(OAc)_2_, AgBF_4_, and TFA,^35^ to afford the C-2-arylated derivative **44**. Subsequent HATU-mediated coupling of this Trp-derivative with the protected glycine scaffold **6** provided **45** in high yield. Finally, treatment of **45** with LiOH·H_2_O led to the acid **46**.

We also envisioned the attachment of the 4-NO_2_-phenyl group to the C-2 position of the indole of Trp through a CO linker. To this end, Fmoc-Trp-OMe (**10**) reacted with 4-NO_2_-benzoyl chloride in the presence of SnCl_4_ in anhydrous DCM at 0 °C, as described for a similar indole derivative,^36^ to afford the C-2 substituted derivative **47**. Unexpectedly, reaction of **47** with piperidine led to the removal of the Fmoc group and concomitant cyclization and aromatization to afford the β-carboline **48**. Previous synthesis of this compound involved a Pictet-Spengler condensation of the Trp derivative with 4-NO_2_-benzaldehyde followed by oxidation of the tetrahydro-β-carboline thus formed.^37^

To avoid this cyclization, the acylation reaction was performed at the trimer **7** using 4-NO_2_-benzoyl chloride (4.5 eq) and SnCl_4_ (9 eq). In this way, the acylated derivative **49** was obtained. Subsequent treatment with LiOH·H_2_O afforded the deprotected trimer **50**.

**Scheme 5:**
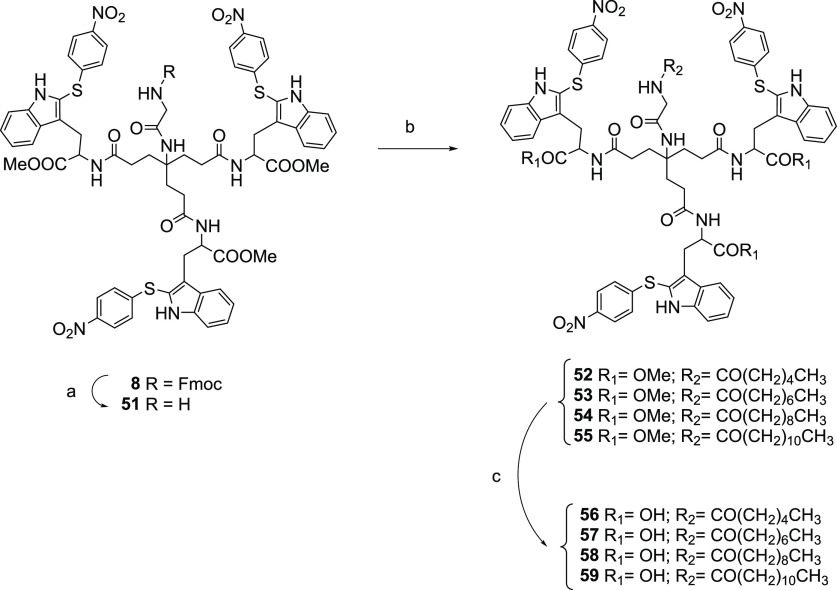
Reagents and Conditions: (a) Piperidine, DCM, rt, 2 h, 73% Yield; (b) the Corresponding Acyl Chloride, Propylene Oxide, Anhydrous DCM, rt, 3.5–5 h, 30–59% Yields; (c) LiOH·H_2_O, THF/H_2_O, rt, 24 h, 41%-Quantitative Yields

As will be later discussed, compound **2** was still the compound providing the best antiviral activity in the VSV-S assay. Thus, the next set of modifications involved the introduction of fatty acid chains of different lengths (from 4 to 10 methylenes) at the NH_2_-group of the glycine moiety at the focal point in compound **2** to modulate the hydrophobicity of the resulting compounds (Scheme 5). Selective NHFmoc deprotection of the glycine intermediate **8** by treatment with piperidine afforded the free NH_2_ derivative **51** (73% yield). Acylation reaction of **51** with aliphatic acyl chlorides with alkyl chains of different lengths in the presence of propylene oxide in dichloromethane afforded derivatives **52**–**55**. Saponification of these methyl esters with LiOH·H_2_O gave the final compounds **56**–**59** (Scheme 5). As will be later discussed, these acyl derivatives maintained antiviral activity in the pseudotyped VSV-S antiviral assay. Thus, other functionalized chains at the focal point were also explored.

**Scheme 6:**
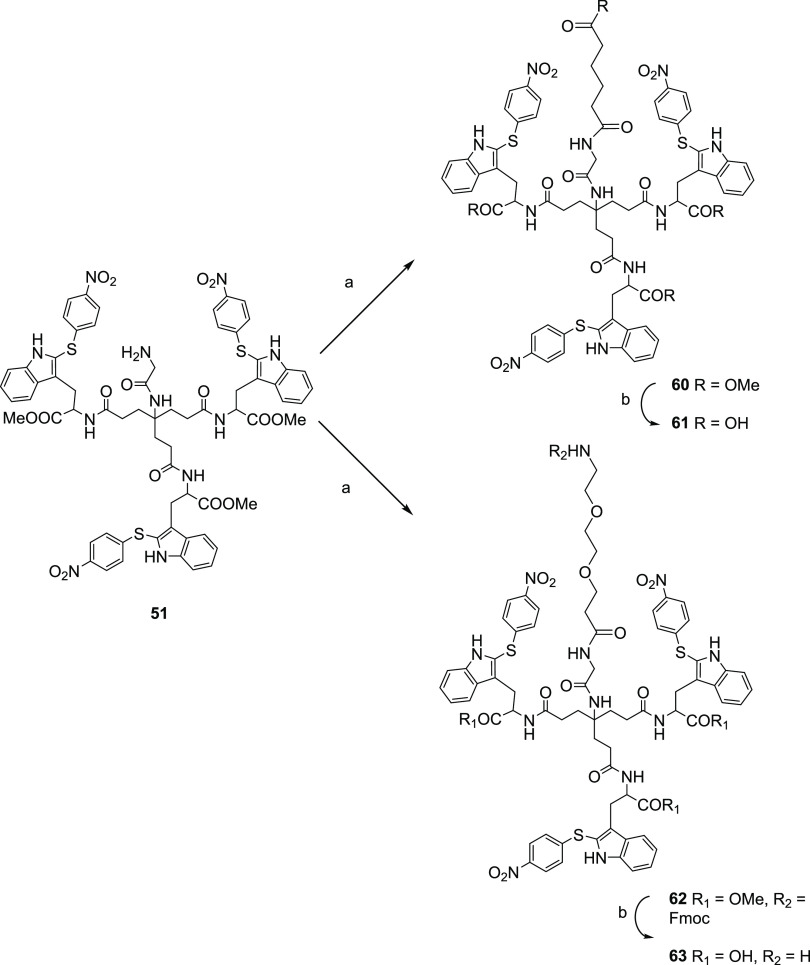
Reagents and Conditions: (a) Monomethyl Adipate for **60**; Fmoc-9-amino-4,7-dioxanonanoic Acid for **62**, HATU, DIPEA, Anhydrous DMF, 30 °C, 24 h, 53–57% Yields; (b) LiOH·H_2_O, THF/H_2_O, rt, 24 h, 60–91% Yields

Reaction of **51** with monomethyl adipate in the presence of HATU and DIPEA afforded the ester derivative **60** in 53% yield (Scheme 6). Saponification of the four ester groups in **60** by treatment with LiOH·H_2_O afforded compound **61** in 60% yield. Similarly, reaction of **51** with Fmoc-9-amino-4,7-dioxanonanoic acid, also with HATU and DIPEA, provided the NHFmoc derivative **62** in 57% yield. Treatment of **62** with LiOH·H_2_O led to the saponification of the methyl ester moieties and concomitant removal of the Fmoc group to provide compound **63** in 91% yield.

**Scheme 7:**
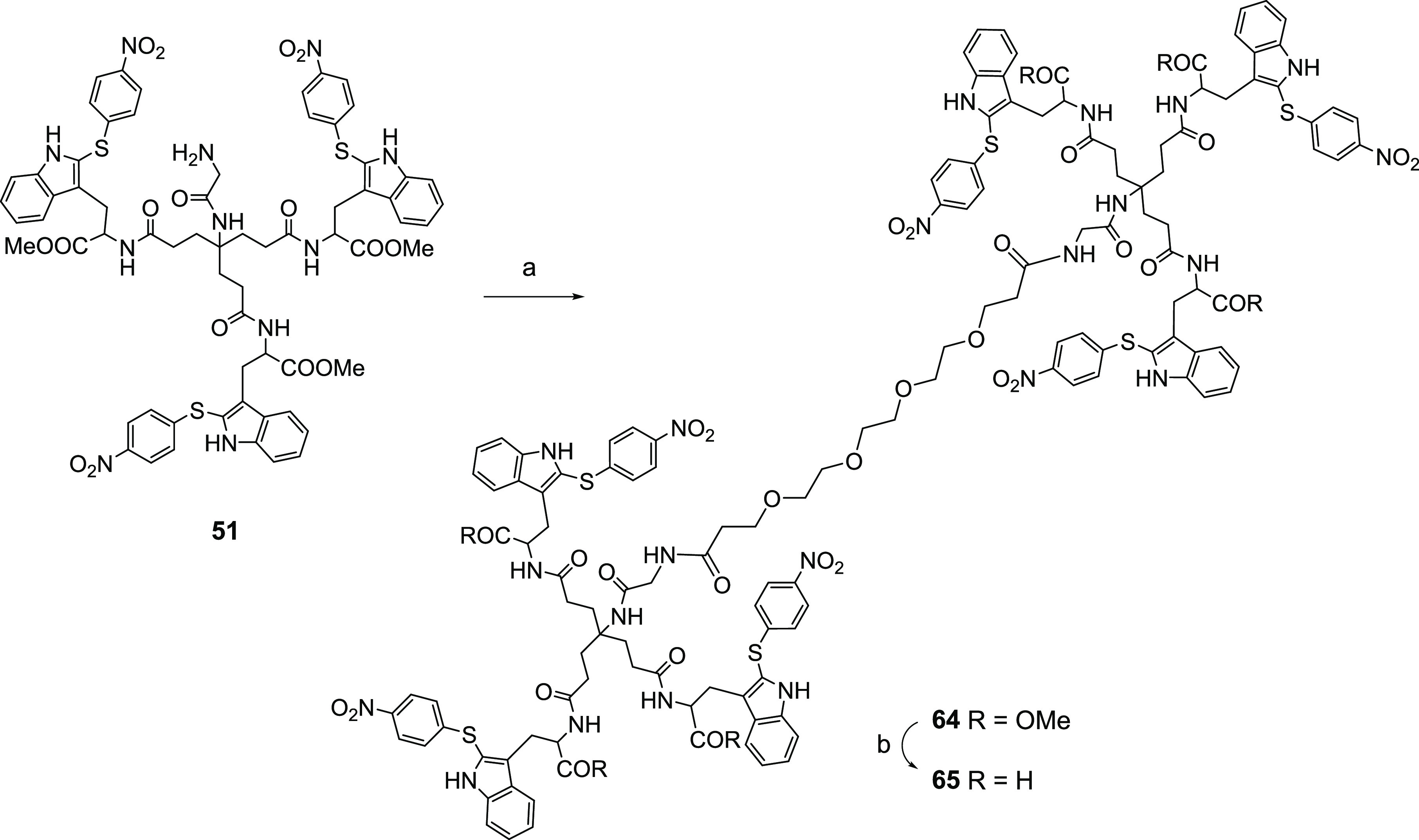
Reagents and Conditions: (a) 4,7,10,13-Tetraoxohexadecane-1,16-dioic Acid, HATU, DIPEA, Anhydrous DMF, 30 °C, 24 h, 65% Yield; (b) LiOH·H_2_O, THF/H_2_O, rt, 48 h, 81% Yield

Finally, we envisioned the synthesis of a dimer containing two units of compound **2** as a way to increase multivalency. Based on the antiviral data obtained with the compounds modified at the focal point (**56**–**59**, **61**, and **63**), a glycol linker was selected as a connecting unit. Thus, reaction of the Gly derivative **51** (2 equiv) with 4,7,10,13-tetraoxohexadecane-1,16-dioic acid (Scheme 7) (1 equiv) in the presence of HATU and DIPEA in DMF at 30 °C for 48 h afforded the dimer **64** in 65% yield. Saponification of the methyl esters by treatment with LiOH·H_2_O at rt overnight led to compound **65** in 81% yield.

In summary, we synthesized new Trp trimers substituted at position 2 of each indole with differently functionalized aryl rings (R_1_ in general formula **I**). In most cases, the aryl ring is connected through a thioether to the C-2 position of the indole (X = S in **I**). Different chains have been incorporated at the NH_2_ group of the Gly moiety at the focal point (R_2_ in **I**), and this approach has been used to synthesize the dimer **65** connecting two units of compound **2** through a glycol spacer.

### Antiviral Evaluation

The compounds were evaluated using the VSV-S assay for their antiviral activity and cytotoxicity. Specifically, VSV-S was mixed with serial dilutions of the compound prior to infection of either Vero E6 or A549-Ace2-TMPRSS2 cells, and the concentration of compound resulting in 50% reduction of virus infection (IC_50_) and 50% reduction in cell viability (CC_50_) values were determined in parallel in the same wells following 16 h of culture (Table 1 and Supplementary Figure S1). Of note, none of the compounds reached 50% cytotoxicity at the highest concentration tested (100 μM), highlighting the low cellular toxicity of these compounds.

**Table 1:** . Antiviral Activity of the Selected Group of Compounds Against VSV-S in Vero E6 and A549-ACE2-TMPRSS2 Cells^a^^,^^b^

	Vero E6 cells		A549-ACE2-TMPRSS2 cells	
compound	IC_50_^c^ (μM)	CC_50_^d^ (μM)	IC_50_^c^ (μM)	CC_50_^d^ (μM)
**1**	21.4 ± 9.5	>100	18.5 ± 3.5	>100
**2**	0.6 ± 0.5	>100	1 ± 0.4	>100
**3**	32.9 ± 4.5	>100	ND	ND
**36**	>100	ND	ND	ND
**37**	37.1 ± 15.9	>100	8.6 ± 4.7	>100
**38**	27.6 ± 12.6	>100	11.6 ± 4.8	>100
**39**	33.6 ± 7.2	>100	34.9 ± 8.7	>100
**40**	33 ± 1.3	>100	46.9 ± 17.5	>100
**41**	3 ± 1.9	>100	2.3 ± 1.7	>100
**42**	>50	>100	ND	ND
**46**	>50	ND	ND	ND
**50**	>50	ND	ND	ND
**56**	18.9 ± 6.8	>100	8.3 ± 1.1	>100
**57**	23.5 ± 3.8	>100	11.1 ± 5	>100
**58**	21 ± 8.7	>100	9.8 ± 4.3	>100
**59**	30.3 ± 5.3	>100	44.4 ± 23.4	>100
**61**	2.1 ± 1.5	>100	2.3 ± 0.8	>100
**63**	0.7 ± 0.9	>100	1.4 ± 1.1	>100
**65**	0.3 ± 0.2	>100	15.8 ± 1.5	>100

a Data represents the mean ± SD of at least three replicates.

b ND: not determined.

c IC_50_: concentration of the compound at which the virus infection is reduced by 50%.

d CC_50_: concentration of the compound at which a 50% reduction in cell viability is observed.

Overall, the antiviral activity of the compounds correlated well between Vero E6 and A549-ACE2-TMPRSS2 cells (Spearmen’s rho = 0.56, *p* < 0.05; Figure S1). As already mentioned in the hit identification section, of the three initial compounds of our home library, compound **2**, a trimer with a NHCOCH_2_NH_2_ at the focal point, showed a more potent antiviral effect than the tetrameric compound **1** or the trimeric compound **3** that has a NO_2_ group at the focal point. Thus, the skeleton of **2** was maintained in the next round of structure–activity relationship (SAR) studies. The data obtained with compounds **36**–**42** highlighted the importance of the substituent on the phenyl ring for the antiviral activity. Moving the 4-NO_2_ group to positions 2 or 3 of the phenyl ring (**36** and **37**, respectively) led to significantly less potent compounds than those with the NO_2_ group at position 4 (as in hit **2**). Replacement of the 4-NO_2_ group in **2** by other electron withdrawing groups (CF_3_ in **38**, CN in **39**, or F in **40**) also led to significantly less potent compounds. Of these derivatives, only **41**, with a COCH_3_ group at position 4, resembled compound **2** in having IC_50_ values around 2 μM in the assays with both cell types. However, a similar compound with a SO_2_CH_3_ group at position 4 (compound **42**) had no antiviral activity. Interestingly, when the 4-NO_2_-phenyl ring is directly attached at the C-2 position of the indole (as in compound **46**) or the 4-NO_2_ phenyl is linked to the C-2 through a CO unit (compound **50**), the antiviral activity is also lost.

Introduction of alkyl chains through acylation of the NH_2_ of the Gly at the focal point (compounds **56**–**59**) also diminished the antiviral activity in Vero E6 cells, although **56**–**58** showed IC_50_ values around 10 μM in A549-ACE2-TMPRSS2 cells. Interestingly, **61**, which carries a four-methylene alkyl chain and distal COOH group, showed IC_50_ values around 2 μM in both cell lines, considerably better than the same analogue with a distal methyl group (compound **56**). An acylated derivative with a glycol chain and terminal amino group (compound **63**) had similar antiviral activity as the hit compound **2**. Finally, the dimer **65** containing two units of **2** linked through a polyethylene glycol spacer had an IC_50_ value of 0.28 ± 0.23 μM in Vero E6 cells. Thus, the data obtained point toward the importance of both the substituent at the aryl ring linked to the Trp and the linking moiety, so that the combination of thioether as the linker and the 4-NO_2_ group at the aryl ring provides the best antiviral activity (compounds **2**, **61**, **63**, and **65**).

### Mechanism of Action Studies in the Cell Culture

**Figure 3:**
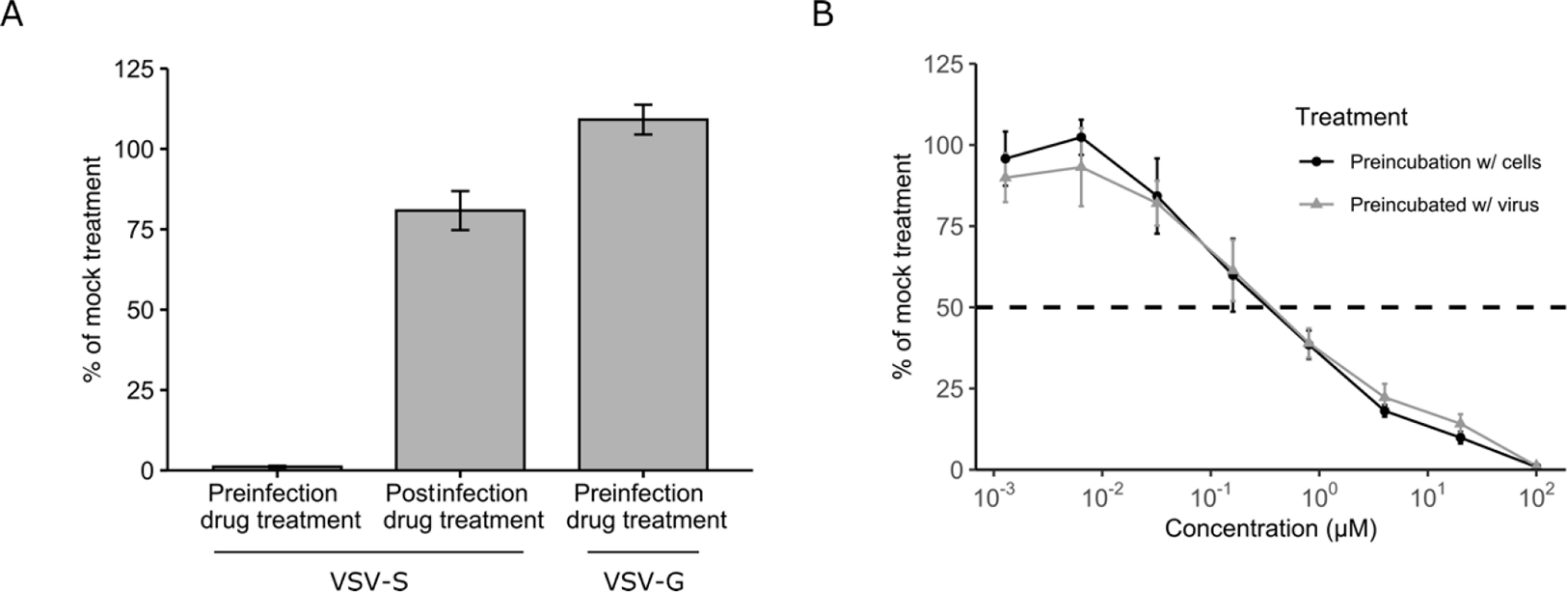
Mechanism of action of compound **2**. (A) Compound **2** at a concentration of 100 μM was preincubated with VSV-S before the virus was added to the cells (preinfection drug-treatment), added 1 h after infection with VSV-S, when the entry process was completed (post-infection drug treatment), or it was preincubated with VSV pseudotyped with the VSV glycoprotein (bar-labeled VSV-G) to assess specificity for S-mediated entry. The degree of infection in each condition was standardized relative to that of infected cells mock-treated with the solvent alone. (B) The effect of preincubating different concentrations of compound **2** with the cells prior to addition of VSV-S (preincubation w/ cells; black line and symbols) or preincubating different concentrations of compound **2** with VSV-S prior to addition to cells (preincubation w/ virus; gray line and symbols). Data represent the mean and SEM of at least three replicates.

As compound **2** and some of its derivatives exhibited low IC_50_ values in the VSV-S assay, we sought to define the mechanism by which these compounds inhibit infection. First, to examine if the compounds acted on the entry step, we compared the antiviral effect of adding the drug during the entry process or one-hour postinfection, when entry has been completed (Figure 3A). For this assay, a high concentration (100 μM) of compound **2** was employed to ensure that even weak effects would be detected. While a strong reduction in infection (assessed as the degree of viral-produced GFP reporter expression) was observed when the compound was added together with the virus, no decrease in infection was observed when the addition of compound **2** was delayed until one-hour post virus addition, when the entry process was completed by removal of the viral inoculum (Figure 3A). Since addition of the compound after infection resulted in a loss of antiviral activity, these data indicate that compound **2** specifically targets the entry process. Next, we evaluated the specificity of compound **2** for interfering with S-mediated entry. For this, we generated a VSV pseudotyped with its native glycoprotein (VSV-G). As all steps in the infection process are identical between VSV-S and VSV-G, with the exception of the glycoprotein used for entry, these viruses can be used to assess the specificity of antivirals targeting S-mediated entry. Unlike for VSV-S, no antiviral activity was observed when VSV-G was preincubated with compound **2** prior to infection of cells (Figure 3A), indicating that compound **2** specifically blocks S-mediated entry. Finally, we compared the effects of preincubation of the compound with the virus for 1 h prior to infection of cell or preincubation of the cells for 1 h, followed by addition of the virus (Figure 3B). In both cases the compound was present when the virus was added to the cells. No significant difference in the antiviral effect at different concentrations of compound **2** was observed (IC_50_ of 0.48 μM ± 0.14 vs 0.32 μM ± 0.13 when preincubated with the virus or with the cells, respectively). This suggests that the binding of compound **2** to its target is faster than the interaction of the virus with its receptor.

### Validation of Antiviral Activity against SARS-CoV-2

To validate the results obtained with the VSV-S pseudotype assay, we next tested compounds **2**, **63**, and **65** against SARS-CoV-2 infection in two cell lines that support robust virus replication: Vero E6-TMPRSS2 and A549-ACE2 cells (Table 2). For this, a SARS-CoV-2 virus carrying the D614G S mutation was preincubated with diluent alone (mock) or with the compounds at 10 μM prior to addition to the cells, and virus production was assayed after 24 h via limiting dilution. Remdesivir (10 μM) was used as a positive control. All compounds reduced virus production by >98% in A549-ACE2 cells and by >89% in VeroE6-TMPRSS2 cells, confirming the strong antiviral activity of these compounds against SARS-CoV-2.

**Table 2:** . Antiviral Activity of Selected Compounds against Genuine SARS-CoV-2 in Two Susceptible Cell Lines^a^

		relative virus production vs mock-treatment	
compound	concentration (μM)	A549-ACE2 cells	VeroE6-TMPRSS2 cells
**2**	10	0.017 ± 0.000	0.039 ± 0.008
**63**	10	0.035 ± 0.032	0.059 ± 0.018
**65**	10	0.011 ± 0.005	0.109 ± 0.018
remdesivir	10	0.002 ± 0.002	0.002 ± 0.001

a Data represents the mean ± S.D. of three replicates.

### Antiviral Activity of Selected Compounds against the Omicron BA.1 Variant

SARS-CoV-2 has undergone significant evolution since its emergence. To assess whether the identified compounds could inhibit the replication of newer SARS-CoV-2 variants, we next studied the ability of compounds **2**, **63**, and **65** to inhibit the replication of pseudotyped VSV carrying the S protein of the SARS-CoV-2 Omicron BA.1 virus (VSV-S_Omicron_) compared to VSV carrying the S protein of the Wuhan-Hu-1 strain (VSV-S_Wuhan-Hu-1_) used for the initial screening (Table 3). Vero E6-TMPRSS2 cells were used in these experiments as they displayed higher susceptibility to infection by BA.1 pseudotyped VSV-S (data not shown). All three compounds showed antiviral activity against pseudotyped VSV-S_Omicron_ at noncytotoxic concentrations. However, IC_50_ values were 1–3 orders of magnitude higher for VSV-S_Omicron_ than for VSV-S_Wuhan-Hu-1_ (Table 3). These data can be explained by the large number of mutations that the Omicron BA.1 variant accumulates in the S gene (35 mutations). Nevertheless, compound **65** exhibited moderate antiviral activity against this omicron variant, with an IC_50_ value <10 μM in VSV-S_Omicron_ assays.

**Table 3:** . Antiviral Potency of Three Selected Compounds Using Pseudotyped VSV Carrying the S Protein of Wuhan-Hu-1 or VSV-S Omicron BA.1 in Vero E6-TMPRSS2 Cells^a^

	IC_50_ ± SD (μM)	
compound	Wuhan-Hu-1	Omicron BA.1
**2**	0.08 ± 0.07	21.73 ± 0.83
**63**	0.01 ± 0.00	25.43 ± 0.00
**65**	0.28 ± 0.23	6.24 ± 0.16

a Data represents the mean and S.D. of three replicates.

### Thermofluor Assays Indicate that Active Compounds Bind to the Receptor Binding Domain of the S Protein

Having proven that the synthesized compounds inhibit SARS-CoV-2 infection by preventing viral entry, the next step was to determine if they interact with the S protein. With this aim, we performed a qualitative test using thermofluor assays.^38^ This technique monitors changes in fluorescence of a protein-binding dye resulting from protein unfolding in solution due to a gradual increase of temperature. The binding of a ligand can modify the thermal stability of a protein, which changes the fluorescence profile obtained. The impact of such binding can be quantified by the increase or decrease of the temperature (*T*_m_) at which the increase in fluorescence is 50% of the maximum fluorescence change. The target protein in our case was the purified recombinant RBD domain, produced in a baculovirus/insect cell system to guarantee its glycosylation (see the Experimental Section).

**Figure 4:**
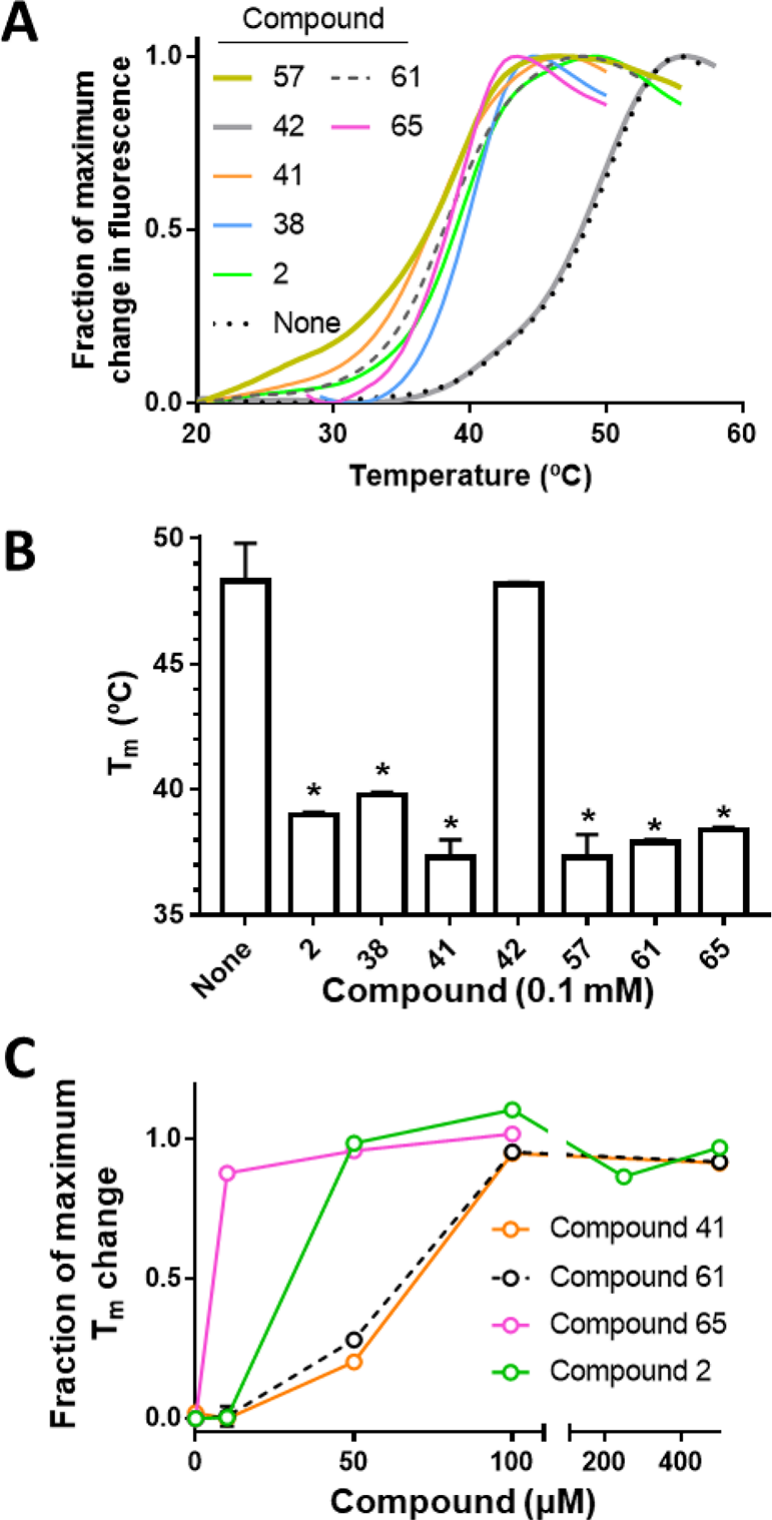
Influence of the investigated compounds on the thermofluor profiles and *T*_m_ values of the RBD. (A) Fluorescence profiles with a gradual thermal increase for the recombinantly produced RBD in the presence of 100 μM of the indicated compounds. The profiles represent the mean of at least three replicates. The fluorescence change is given as a fraction of the maximum change for the observed transition. (B) Temperatures for 50% of the maximum fluorescence change (*T*_m_) for the RBD in the absence (None) or in the presence of 100 μM of the indicated compounds. Data are means ± SD of two or more replicates. Statistical significance (Dunnet multiple comparisons test versus the None column in one-way ANOVA) is marked by * (*P* ≤ 0.0001). (C) Changes in *T*_m_ for RBD with increasing concentration of the indicated compounds. Results are expressed as a fraction of the extrapolated maximum change inferred from sigmoidal fitting of the experimental results (fitting not shown).

Figure 4A shows the fluorescence profile for the RBD domain in the absence of the ligand, and the shift of the curve toward lower temperatures in the presence of a fixed concentration (100 μM) of compounds **2**, **38**, **41**, **57**, **61**, and **65**. All these compounds had been found to inhibit viral entry in the cellular assays (Table 1). In marked contrast, compound **42**, which did not significantly inhibit viral entry, did not cause any change in the curve (Figure 4A). Figure 4B illustrates the differences in *T*_m_ values found in the presence of these compounds relative to the ligand-free protein, showing for all of them except **42**, differences that are statistically significant.

Next, we tested the effect of variable concentrations of four of these compounds (**2**, **41**, **61**, and **65**) on the binding (Figure 4C). Our results showed that compounds **2** and **65**, the two entry inhibitors that showed the lowest IC_50_ in the cellular infection assays (IC_50_: 0.6 and 0.3 μM, respectively), caused RBD destabilization at lower concentrations than **41** and **61**, which exhibited higher IC_50_ in cellular infection assays (IC_50_: 3 and 2.1 μM, respectively).

In summary, the thermofluor assays indicate that the compounds that have a substantial effect on blocking viral entry are able to bind the RBD domain of the S protein.

### Microscale Thermophoresis (MST) Provides Additional Proof of Binding of Active Compounds to the S Protein, Revealing Competition with ACE2 Binding

**Figure 5:**
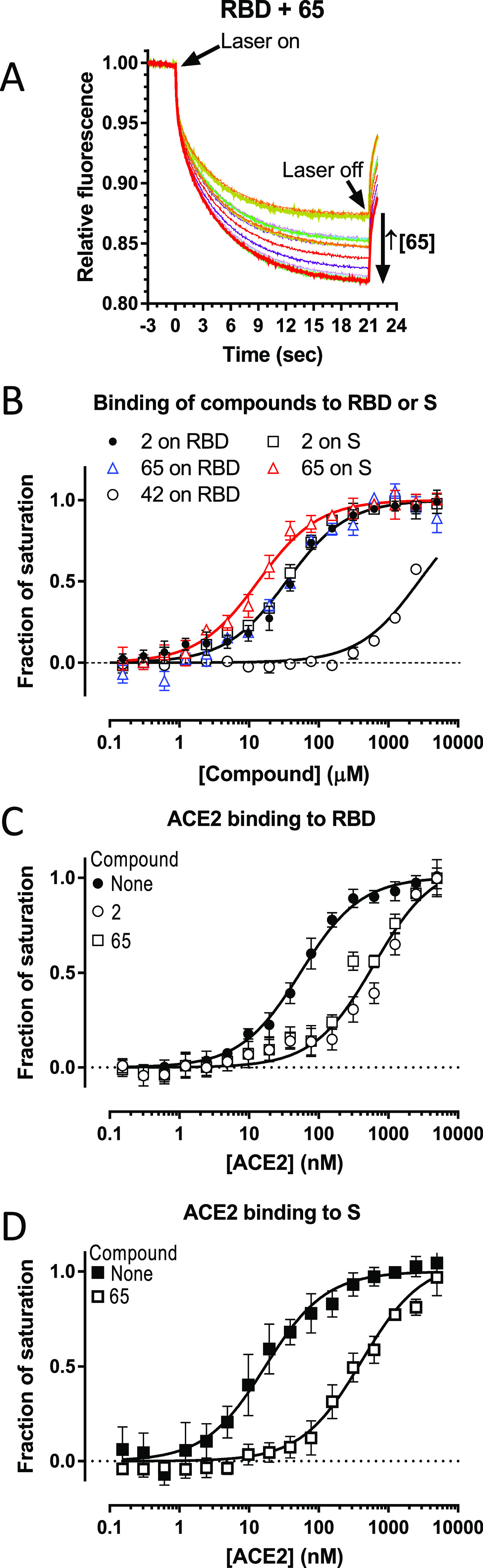
Microscale thermophoresis (MST) results. (A) Crude MST traces exemplified for fluorescently labeled RBD (50 nM) in the presence of increasing concentrations of compound **65** (in 2-fold steps, range 0.15 μM to 5 mM, see the Experimental Section; each step in a different rainbow color). (B–D) Hyperbolic fitting of the plots of fractional fluorescence change arising from fluorescently labeled RBD or S (as indicated in the figures) at different concentrations of: (B) compounds **2**, **65**, and **42**; (C, D) the extracellular catalytic domain of human ACE2 (see the Experimental Section) in the absence or in the presence of 0.5 mM of **2** or **65**, as indicated. The fraction of saturation was estimated for each concentration of the ligand as the quotient (*F_x_* – *F*_0_) / (*F*_∞_ – *F*_0_), where *F*_0_, *F_x_*, and *F*_∞_ are the fluorescence in the absence, at a given concentration, and at infinite concentration of the ligand that is varied, respectively. *F*_∞_ was estimated from the hyperbolic fitting. In the case of compound **42**, the data were fitted to the minimal possible value of *K*_D_ accepted by the fitting program (Graphpad Prism). Curves correspond to hyperbolic fitting (in semilog representation). Each point is the mean (±SE) for three different titrations. The *K*_D_ values are the concentrations giving a half-maximum change. In panel B, given the lack of statistical differences in the *K*_D_ values for **2** versus RBD and versus S, and of **65** versus RBD, a single hyperbola has been drawn fitting all the clumped points for these three data sets (*K*_D_, 34.6 μM). In panel C, a single hyperbola is shown for the clumped results for **2** and **65**, given the lack of statistically significative differences between them (*K*_D_ values of ACE2 for RBD, 55.1 ± 4 and 618 ± 86 nM in the absence or presence of the compounds, respectively). In panel D, *K*_D_ values for the binding of ACE2 to S were 17.2 ± 2.2 and 388 ± 45 nM in the respective absence and presence of 0.5 mM **65**.

Further and more quantitative evidence of active compound binding to the S protein in its RBD was obtained using MST, a technique that titrates the influence of ligand concentration on the fluorescence change when heat is applied locally to capillary tubes hosting solutions of the fluorescent macromolecule and the ligand.^39^ We used purified RBD and S proteins fluorescently labeled via the binding of a fluorophore to their respective polyHis-tags, titrating the effects of increasing concentrations of compounds **2** and **65** (the compounds that showed the highest potency in the cell culture, see above) as exemplified in Figure 5A. From the fluorescence traces obtained, the fraction of saturation of the protein by each concentration of the ligand can be estimated (see the legend of Figure 5).

Figure 5B illustrates the results of these binding assays, which fit single-site binding. *K*_D_ values did not differ significantly for binding of **2** or **65** to the RBD, or for the binding of **2** to the complete S protein (mean ± SE values, 37.8 ± 4.6, 35.4 ± 5.6, and 30.6 ± 3.0 μM, respectively), while the *K*_D_ value for the binding of **65** to S was somewhat lower (*K*_D_, 13.6 ± 1.7 μM; *p* < 0.05). Compound **42** was also tested and was found to be a very poor binder (*K*_D_ ≥ 2.5 mM for binding to the RBD, Figure 5B), in line with its lack of substantial effects on VSV-S cell entry assays (Table 1) and on the thermal stability of the RBD (Figure 4B). Thus, these results confirm that compounds **2** and **65** bind to the S protein in its RBD.

Next, the binding of the unlabeled recombinant catalytic domain of ACE2 to the fluorescent RBD domain was titrated in the absence and presence of 0.5 mM of **2** or **65**. Our results showed that the concentrations of ACE2 needed for binding to the RBD domain were significantly (∼10-fold) increased by the presence of 0.5 mM of any of these two compounds (Figure 5C). From this experiment, we concluded that compounds **2** and **65** compete with the cellular receptor ACE2 for the binding to the RBD domain. This competition was also observed when titrating the binding of ACE2 to the complete S protein, as exemplified for compound **65** in Figure 5D. These results help to explain how the synthesized compounds could act as entry inhibitors.

### Detection of Compound **2** in the Structure of the Viral Spike Explains Viral Cell Entry Inhibition

Prior structural work at near-atomic resolution has shown S to be homotrimeric and to adopt distinct confirmations that can influence its ability to bind hACE2. Specifically, the RBD domains in the S trimer can adopt an “up” conformation or be buried inside S (“down” conformation). Only S trimers with one RBD in the up position (one RBD-up) can bind hACE2, which then promotes adjacent RBDs to adopt an open conformation that further increases the binding affinity for ACE2.^40,41^

**Figure 6:**
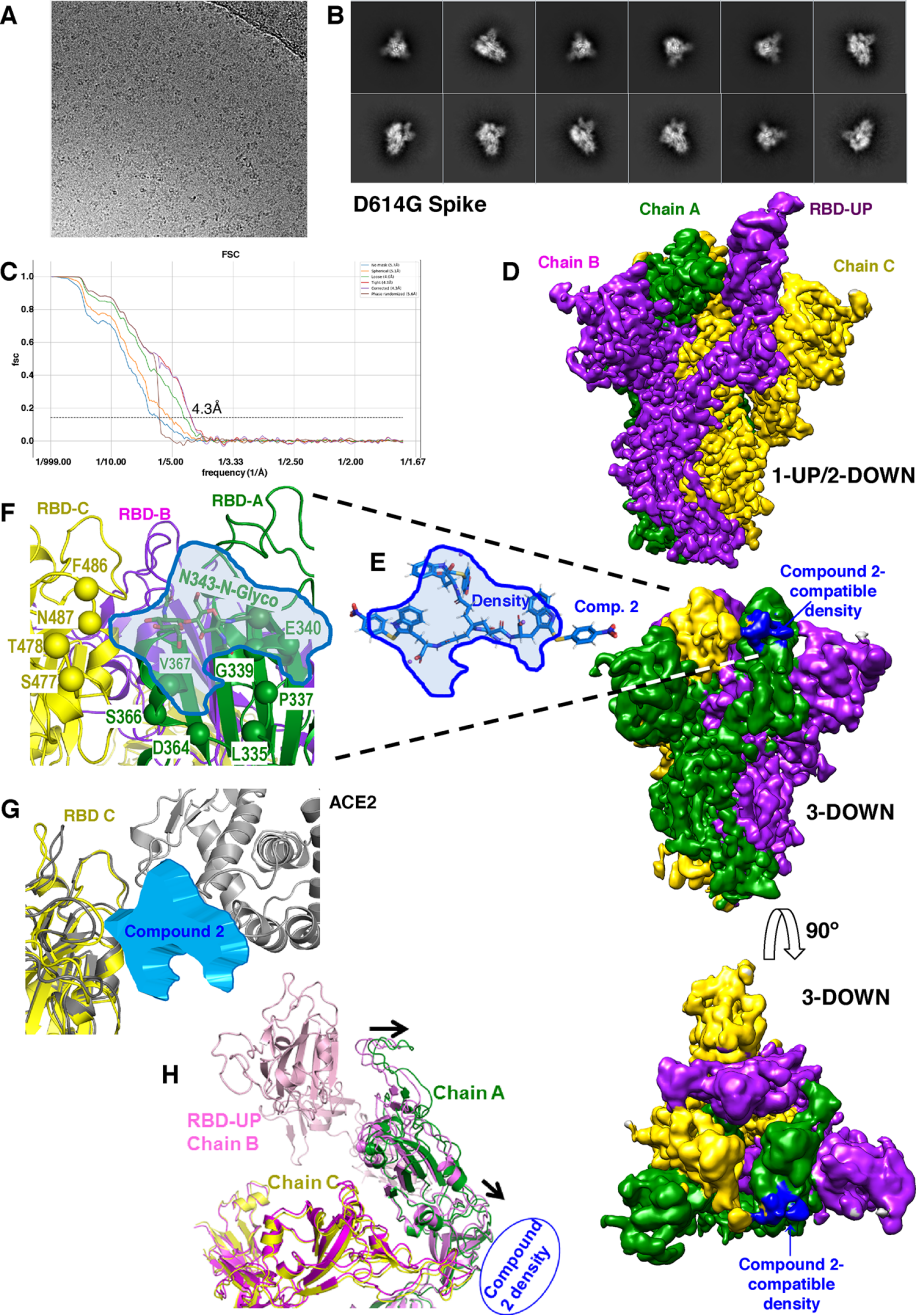
CryoEM imaging sheds light on compound **2** binding to the spike and on its interference with viral entry. (A) Representative cryoEM micrograph of the purified S:D614G protein (see the Experimental Section). (B) Set of representative top and side view class averages obtained after reference-free 2D classification of automatically picked and extracted particles. (C) Fourier Shell Correlation (FSC) resolution curves of the spike in 3-down conformation, shown as the regular cryoSPARC global FSC resolution output, which includes no mask and different masks. Resolution, based on the gold standard 0.143 criterion, is 4.3 Å. (D) Cryo-EM maps of the S:D614G protein homotrimer with one RBD in the up position and two RBDs in down positions (top), or with the three RBDs in down positions (middle and bottom). Each subunit is shown in a different color (green, purple, and yellow). The top and middle panels represent side views of the spike with the molecular 3-fold axis of the trimer vertical, while in the bottom panel, the spike is seen from outside the virus, and the molecular threefold axis is perpendicular to the page. The nonprotein density observed (colored blue) in the middle and bottom panels appears to correspond to bound compound **2**, present in the solution (see the Experimental Section), given the reasonably good gross fitting of this compound (in sticks) to the profile of the nonprotein density (panel E, in blue). (F) Zoom on this density profile to show nearby residues from two subunits. The Cα atoms of the indicated residues are localized with spheres in the backbone (yellow or green depending on the subunit) and are identified in single-letter amino acid notation. The N-glycosylation of N343 was visible on the map, and it is represented in sticks. (G) Superimposition of the structure of the backbone of the RBD of subunit C (colored yellow) of the spike in the 3-down conformation observed here, with that of the RBD (in gray) in the RBD-ACE2 complex (PDB 7A94(41)), to illustrate that the density (blue) attributed here to compound **2** sits at the part of RBD that interacts with ACE2, strongly suggesting interference of compound **2** with the interaction of the spike with its receptor. (H) Partial view of the 1-up/2-down structure (in different shades of pink depending on the subunit) of the spike observed here, superimposed on the 3-down structure also observed here (in green and yellow). The RBDs of the A and C subunits of the two structures are superimposed, while subunit B of the three-down structure is not seen. In the two RBDs that are equally positioned in the two structures, there is some shift in the position of subunit A in the three-down structure (highlighted by the arrows) away from the position of the up RBD, expectedly destabilizing this up position. Given the location of the extra density equated here with compound **2** (symbolized with a blue ellipse), the binding of his compound could stabilize the down position of chain A, away from its optimal position for stabilizing the up position of chain B.

We obtained the cryo-EM structure of the SARS-CoV-2 S protein bound to compound **2**, used as a prototype of present series of compounds with antiviral cell-entry inhibition. We used an S protein harboring the D614G mutation present in all major SARS-CoV-2 strains except the ancestral Wuhan-Hu-1 strain. From a dataset of 3500 movies (Figure 6A–C), two main conformational populations were isolated by 3D EM data classification, without applying any internal symmetry restriction (see the Experimental Section for details). The most abundant conformation (74% of the particles; 3.4 Å resolution) had one RBD in up position and two in down position per S protein trimer (Figure 6D, top panel). The remaining 26% of the particles had the three RBDs in down positions (4.3 Å resolution; Figure 6D, middle and down panels). Comparison of the present dataset with our recently published datasets,^42^ which were prepared in an identical manner in the absence of any compound, showed that the 3-down class of the spike is highly enriched in the presence of compound **2** (24% of the particles versus <2% of the particles without drug). This finding is further supported by similar cryo-EM structures reported by others in which the conformation with the 3 RBDs down was represented in a small minority of the particles.^41,43^ Hence, compound **2** appears to specifically induce a three RBD-down class that should inhibit binding to ACE2. Indeed, the structure of the spike in the one RBD-up conformation found here is very similar (root mean square, RMSD, of 1.27 Å for the superimposition over 3219 Cα atoms) to that of the same spike variant in the absence of compound^42^ (PDB ID:7QDH). Detailed inspection did not reveal remarkable conformational differences nor additional nonprotein densities that could be attributable to the binding of compound **2**. However, in the structure derived from the three RBD-down map, a clear density was found next to and between two RBDs (Figure 6D, middle and down panels).

Although the local resolution of the map was not optimal in the site of this density, the approximate size of this extra density grossly fitted what would be expected for compound **2** (Figure 6E). The limited resolution precludes more accurate compound **2** fitting into the density, or an unequivocal identification of residues interacting with this compound, although it is possible to confidently assign the closest residues to this drug (Figure 6F). These residues are: N343 (with clear structural evidence of its glycosylation), L335, P337, G339, E340, D364, S366, and V367, all from subunit A, and S477, T478, F486, and N487 from the neighboring subunit C. Some of these residues are mutated in the Omicron BA.1 variant (G339D, S477N, and T478K) that may account for the reduced potency observed for the omicron variant (Table 3). Apart from the extra density assigned to compound **2**, the spike in three RBD-down conformation is overall quite similar to other spikes having in their structures, all their RBDs in a down position. This is reflected in RMSDs of 2.02–2.04 Å for the superposition over the entire spike structures (2699 and 2916 Cα, respectively) in PDB IDs 7KRQ(44) and 7BNM.^41^ The three subunits in the S protein form having its three RBDs in the “down” position are highly similar (RMSDs < 1 Å for mutual superpositions).

Biophysical *in vitro* assays showed that compound **2** inhibits the interaction of the isolated RBD domain with the ACE2 receptor (see Figure 5C above), suggesting direct competition of compound **2** with the ACE2 receptor for interaction with the RBD. This suggestion is substantiated structurally here, since ACE2 and compound **2** share the same RBD binding interface (Figure 6G). This primary inhibitory mechanism could be enhanced if compound **2** physically holds two out of three RBDs of the spike in their down position, preventing the establishment of proper contacts of these RBDs with ACE2. Further enhancement of the inhibitory effect could be due to the possibility of an allosteric effect by which compound **2** binding could prevent the adoption of the up position in the third of these RBDs. This is supported by the facts that, in the spikes in 1-up or 2-up conformations, the RBDs in up position/s interact with neighboring RBDs in the down position,^41−43^ while in the present 3-down structure, the contact with compound **2** prevents an optimal location of the compound-contacting subunits to stabilize the third RBD in the up position (Figure 6H).

In summary, our structures confirm the inference of the thermofluor and thermophoresis studies that compound **2** hampers RBD and S protein binding to the ACE2 receptor as a consequence of the binding of this compound to the S protein in its RBD portion, in a part of the protein that directly interacts with the ACE2 receptor. The cryoEM structures strongly suggest other more indirect effects, largely of allosteric nature, also resulting in competitive inhibition with respect to ACE2 binding. In this respect, compound **2**, and by extension, other cell entry inhibitory compounds in our series, would act by a combination of various molecular mechanisms, all derived from the binding of the compound to the RBD domain in the trimeric S protein. The presence of compound **2** at the boundary of the RBD domain with the remainder of S (Figure 6D, down panel) could potentially explain the increased affinity of compound **65** for S relative to compound **2** (Figure 5B), the earlier being a dimeric variant of compound **2**. Thus, the second unit of compound **2** contained in **65** may extend from the RBD domain to other parts of the same S subunit or even to other S subunits. Alternatively, the larger size of compound **65** might impose stronger allosteric restrictions on the S trimer simply by causing steric clashes. Further structural work using compound **65** will be required to try to differentiate between these possibilities, although a definitive answer may not be possible if the extra part of **65** (relative to compound **2**) extends out freely in the S trimer and is not visible in the structures. In contrast with what is observed with compounds **2** and **65**, compound **42** did not substantially inhibit cell entry in the VSV-S system (Table 1), it had no effect on the thermal stability of the RBD in thermofluor assays at 100 μM concentration (Figure 5B) and exhibited extremely poor affinity for the RBD (Figure 6B). All these effects can be attributed to the presence in the structure of **42** of the 4–SO_2_CH_3_ thiophenyl substituent instead of the 4–NO_2_ group present in compound **2**. It is interesting that compound **41**, bearing a 4–COCH_3_ group as the thiophenyl substituent, inhibited cell entry of the VSV-S virus (Table 1) and thermally destabilized the RBD at 100 μM (Figure 5B). Thus, the −SO_2_– moiety seems to be responsible for the lack of interaction of **42** with the RBD domain, inferring a paramount role of the substituents of the C-2 thiophenyl ring. Unfortunately, the low resolution of the structure of bound compound **2** precludes a detailed assessment of the ways in which the three 4-NO_2_ thiophenyl substituents interact with the S protein.

## Conclusions

In conclusion, we identify and explore, through the synthesis of a variety of structural analogues, a novel class of SARS-CoV-2 inhibitors. We further provide a detailed characterization of their antiviral activity and mechanism of action. The results indicate a direct role in the inhibition of viral entry by binding to the S protein mainly in the RBD domain, and in preventing the adoption by trimeric S of a conformation that enables efficient binding to the host receptor, ACE2. This new family of compounds opens a new avenue for the potential prophylactic (e.g., nasal spray) or combinatorial therapy of SARS-CoV-2 infection that requires further exploration. Compound **65** retained some antiviral activity against the omicron BA.1 variant despite the many amino acid changes in the S protein (15 changes in the RBD alone, relative to Wuhan RBD), supporting future attempts to develop more active compounds of this family for omicron viral variants. Their multivalent character might endow these compounds with particular resistance to viral escape. In this respect, it is also remarkable that different members of this family are active against a diverse group of viruses, such as HIV and picornaviruses, supporting their interest as useful prototypes for future medicinal chemistry studies.

## Experimental Section

### Chemistry Procedures

Melting points were measured on a M170 apparatus (Mettler Toledo, Columbus, Ohio, USA) and are uncorrected.

^1^H and ^13^C NMR spectra were recorded on a Varian INNOVA (now Agilent, Santa Clara, CA, USA) 300 operating at 299 MHz (^1^H) and 75 MHz (^13^C), respectively, a Varian INNOVA-400 operating at 399 MHZ (^1^H) and 99 MHz (^13^C), respectively, and a VARIAN SYSTEM-500 operating at 499 MHz (^1^H) and 125 MHz (^13^C), respectively. Monodimensional ^1^H and ^13^C spectra were obtained using standard conditions. 2D-inverse proton detected heteronuclear one-bond shift correlation spectra were obtained using the pulsed field gradient HSQC pulse sequence. Data were collected in a 2048 × 512 matrix with a spectral width of 3460 Hz in the proton domain and 22,500 Hz in the carbon domain and processed in a 2048 × 1024 matrix. The experiment was optimized for one bond heteronuclear coupling constant of 150 Hz. 2D inverse proton-detected heteronuclear long-range-shift correlation spectra were obtained using the pulsed field gradient HMBC pulse sequence. The HMBC experiment was acquired in the same conditions that HSQC experiment and optimized for long range coupling constants of 7 Hz.

Compounds were also analyzed by HPLC/MS with a Waters e2695 LC (Waters, Milford, Massachusetts, USA), coupled to a Waters 2996 photodiode array detector and a Waters Micromass ZQ. The column used was a Waters SunFire C18 (2.1 × 50 mm, 3.5 μm), and the mobile phases were A: acetonitrile; B: H_2_O, together with a constant 5% of C (H_2_O with 2% formic acid) to assure 0.1% formic acid along the run. For high-resolution mass spectrometry (HRMS), an Agilent 6520 accurate mass quadrupole time-of-flight (QTOF) platform coupled with LC/MS and equipped with an electrospray interface (ESI) working in the positive-ion (ESI+) and negative-ion (ESI−) modes was used.

Analytical TLC was performed on silica gel 60 F_254_ (Merck, Dramstand, Germany)-precoated plates (0.2 mm). Spots were detected under UV light (254 nm) and/or charring with ninhydrin or phosphomolibdic acid.

Separations on silica gel were performed by preparative centrifugal circular thin-layer chromatography (CCTLC) on a Chromatotron^R^ (Kiesegel 60 PF_254_ gipshaltig (Merck)), with layer thicknesses of 1 and 2 mm and flow rates of 4 or 8 mL/min, respectively.

For HPLC analysis, an Agilent Technologies 1120 Compact LC with a reverse-phase column ACE 5 C18-300 (4.6 mm × 150 mm, 3.5 μm) equipped with a PDA (photo diode array) detector was used. Acetonitrile 0.05%TFA was used as mobile phase A, and water 0.05% of TFA was used as mobile phase B with a flow rate of 1 mL·min^–1^, for 10 min, moving from 10 to 100% phase A. Final compounds had purities >95% based on HPLC.

### General Procedure for the Simultaneous Deprotection of Fmoc and Methyl Ester Groups (General Procedure A)

To a solution containing the corresponding methyl ester derivative (1.0 mmol) in THF (20 mL) at 0 °C (ice bath), a solution of LiOH·H_2_O (2 equiv for each methyl ester group) in water (4 mL) was added, and the mixture was stirred at room temperature overnight. Then, 1 N hydrochloric acid aqueous solution was added to reach pH = 2, and volatiles were evaporated to dryness. The residue was dissolved in ethyl acetate (15 mL) and washed with H_2_O (3 × 10 mL). The organic layer was dried over anhydrous Na_2_SO_4_, filtered, and evaporated to dryness. The purification procedures are described individually.

### General Procedure for the Coupling Reaction between the Carboxylic Acids and NH_2_ Group of the Amino Acids (General Procedure B)

To a solution containing the tripodal polyacid **6**(27) (1.0 mmol), HATU (1.3 equiv of each carboxylic acid group), and the appropriate NH_2_ free amino acid (1.1–1.2 equiv of each carboxylic acid group) in DMF (10 mL), DIPEA (2.3 equiv of each carboxylic acid group) was added. The resulting mixture was heated to 30 °C for 24–48 h. Then, it was quenched with a saturated solution of NH_4_Cl (5 mL) and volatiles were removed. The residue was dissolved in ethyl acetate (20 mL) and washed with water (10 mL). The organic layer was dried over Na_2_SO_4_, filtered, and evaporated to dryness, and the residue was purified as indicated for each compound.

### General Procedure for the Sulfenylation Reaction (General Procedure C)

Fmoc-Trp-OMe (**10**)^29^ (1.0 mmol), the corresponding aromatic disulfide (0.7–1.2 mmol) and I_2_ (0.6 mmol) were placed in a sealed tube and dissolved in acetonitrile (4 mL). The resulting mixture was heated at 60 °C for 3–6 h. Then, it was diluted with ethyl acetate (20 mL) and washed with an aqueous solution of NaHSO_3_ (10 mL). The organic layer was dried over Na_2_SO_4_, filtered, and evaporated to dryness, and the residue was purified by flash chromatography.

### General Procedure for the Synthesis of Disulfides (General Procedure D)

To a solution containing the corresponding benzenesulfonyl chloride (1.0 mmol) in anhydrous DMF (3 mL), a solution of TBAI (3.0 mmol) in anhydrous DMF (3 mL) was added dropwise. The resulting solution was stirred at rt for 24 h. Then, it was diluted with DCM (20 mL) and quenched with an aqueous solution of Na_2_S_2_O_3_ (10 mL). The organic layer was washed with a saturated solution of NaHCO_3_ (10 mL), dried over Na_2_SO_4_, filtered, and evaporated to dryness, and the residue was purified by flash chromatography.

### General Procedure for Selective Fmoc Deprotection (General Procedure E)

The appropriate Fmoc-protected compound (1 mmol) was dissolved in DCM (10 mL), and then piperidine (10 mmol) was added dropwise. The reaction was stirred at rt for 2–3 h. Volatiles were removed, and the residue was purified as indicated for each compound.

#### Tetramer **5**

To a cold solution of tetramer **4**(21) (50 mg, 0.24 mmol) in THF (8 mL), the commercially available *p*-nitrophenylsulfenyl chloride (46 mg, 1.41 mmol) and TFA (2 mL) were added. The solution was allowed to reach room temperature and then stirred for 2 h. Then, a solution of aqueous NaOH was added to reach pH = 7. Elimination of the solvent left a residue, which was dissolved in ethyl acetate (30 mL) and washed successively with saturated solutions of NaHCO_3_ (3 × 20 mL) and brine (1 × 20 mL). The organic phase was dried over anhydrous Na_2_SO_4_, filtered, and evaporated to dryness. The residue was concentrated and purified by CCTLC (hexane:ethyl acetate, 7:3) to afford 53 mg (71%) of **5** as an amorphous yellow solid. ^1^H NMR (300 MHz, CDCl_3_) δ: 2.20 (t, *J* = 5.4 Hz, 8H, CH_2_CH_2_CO), 2.39 (d, *J* = 9.2 Hz, 4H, OCH_2_), 2.75 (d, *J* = 9.1 Hz, 4H, OCH_2_), 3.11–3.23 (m, 16H, β-CH_2_Trp, CH_2_O), 3.41 (dd, *J* = 14.1, 7.0 Hz, 4H, β-CH_2_Trp), 3.61 (d, *J* = 1.9 Hz, 12H, COOCH_3_), 4.91 (m, 4H, α-CHTrp), 6.64 (d, *J* = 8.2 Hz, 4H, NH-Trp), 6.98 (m, 8H, Ar), 7.08–7.21 (m, 12H, Ar), 7.57 (m, 4H, Ar), 7.98 (m, 8H, Ar), 9.64 (br s, 4H, NH-1^*i*^Trp).

#### Tetramer **1**

Following the general procedure A, tetramer **5** (24 mg, 0.01 mmol) in THF (8 mL) was treated with LiOH·H_2_O (4.38 mg, 0.10 mmol) in water (2 mL) and the solution was stirred at room temperature overnight. After workup, the residue was precipitated with cool diethyl ether to afford 20.7 mg (92%) of **1** as an amorphous yellow solid. ^1^H NMR (400 MHz, DMSO-*d*_6_) δ: 2.19 (m, 8H, CH_2_CH_2_CO), 3.11 (m, 4H, β-CH_2_Trp), 3.21–3.37 (m, 20H, OCH_2_, β-CH_2_Trp), 4.53 (m, 4H, α-CHTrp), 7.05 (t, *J* = 7.5 Hz, 4H, Ar), 7.12–7.22 (dd, *J* = 8.2, 6.0 Hz, 12H, Ar), 7.33 (d, *J* = 8.2 Hz, 4H, Ar), 7.71 (d, *J* = 8.0 Hz, 4H, Ar), 8.08 (m, 8H, Ar), 8.17 (d, *J* = 8.2 Hz, 4H, NH-Trp), 11.59 (br s, 4H, NH-1^*i*^Trp). ^13^C NMR (101 MHz, DMSO-*d*_6_) δ: 27.9 (β-CH_2_Trp), 34.8 (CH_2_CH_2_CO), 45.2 (C(CH_2_))_4_), 53.5 (α-CHTrp), 67.4, 69.3 (OCH_2_), 112.0, 119.6, 119.7, 120.1, 120.1, 123.7, 124.7, 125.4, 126.3, 127.8, 128.5, 137.9, 139.6, 145.4, 148.0, 151.9 (Ar), 170.4 (CH_2_CH_2_CO), 173.4 (COOH). HRMS (ESI^+^) *m*/*z*: calcd for C_85_H_80_N_12_O_24_S_4_ 1781.8707; found 1781.6318.

#### Trimer **7**

Following the general procedure B, a solution containing **6**(27) (125 mg, 0.24 mmol), H-Trp-OMe·HCl (200 mg, 0.79 mmol), HATU (360 mg, 0.95 mmol), and DIPEA (454 μL, 2.61 mmol) in anhydrous DMF (2.4 mL) reacted for 24 h. After workup, the residue was subjected to column chromatography (DCM/methanol, 18:1) to yield 250 mg (93%) of **7** as an amorphous white solid. ^1^H NMR (400 MHz, CDCl_3_) δ: 1.41 (m, 3H, CH_2_CH_2_CO), 1.62 (m, 3H, CH_2_CH_2_CO), 1.82 (m, 6H, CH_2_CH_2_CO), 3.00 (dd, *J* = 14.9, 8.4 Hz, 3H, β-CH_2_Trp), 3.21 (dd, *J* = 14.9, 4.5 Hz, 3H, β-CH_2_Trp), 3.57 (s, 9H, COOCH_3_), 4.08 (m, 2H, COOCH_2_CH), 4.22 (dd, *J* = 10.2, 7.1 Hz, 1H, COOCH_2_CH), 4.72 (td, *J* = 8.1, 4.5 Hz, 3H, α-CHTrp), 6.88 (s, 3H, Ar), 6.93 (m, 3H, NH-Trp), 6.99 (t, *J* = 7.5 Hz, 3H, Ar), 7.11–7.17 (m, 8H, Ar), 7.28 (t, *J* = 7.4 Hz, 2H, Ar), 7.39 (d, *J* = 7.7 Hz, 3H, Ar), 7.45 (dd, *J* = 16.5, 7.5 Hz, 2H, Ar), 7.65 (d, *J* = 7.6 Hz, 2H, Ar), 8.80 (s, 3H, NH-1^*i*^Trp).

#### Trimer **8**

To a cold solution of trimer **7** (50 mg, 0.04 mmol) in formic acid (8 mL), the commercially available *p*-nitrophenylsulfenyl chloride (25.2 mg, 0.13 mmol) was added. The solution was allowed to reach room temperature and then stirred for 2 h. Then, a solution of aqueous NaOH was added to reach pH = 7. Elimination of the solvent left a residue, which was dissolved in ethyl acetate (30 mL) and washed successively with saturated solutions of NaHCO_3_ (3 × 20 mL) and brine (1 × 20 mL). The organic phase was dried over anhydrous Na_2_SO_4_, filtered, and evaporated to dryness. The residue was concentrated and purified with a Biotage HPFC (high-performance flash chromatography) purification system on a reverse phase using water/acetonitrile (100:0 to 0:100) as an eluent, frozen, and lyophilized, yielding 35.2 mg (50%) of **8** as a yellow solid. Mp (decomp at 135 °C). ^1^H NMR (400 MHz, CDCl_3_) δ: 1.51 (m, 3H, CH_2_CH_2_CO), 1.72 (m, 3H, CH_2_CH_2_CO), 1.95 (m, 6H, CH_2_CH_2_CO), 2.83 (s, 2H, α-CH_2_Gly), 3.19 (dd, *J* = 14.3, 7.4 Hz, 3H, β-CH_2_Trp), 3.38 (dd, *J* = 14.4, 5.3 Hz, 3H, β-CH_2_Trp), 3.64 (s, 9H, COOCH_3_), 4.15 (t, *J* = 7.1 Hz, 1H, COOCH_2_CH), 4.30 (m, 2H, COOCH_2_CH), 4.87 (m, 3H, α-CHTrp), 5.39 (br s, 1H, NHCOCH_2_NH), 6.61 (br s, 3H, NH-Trp), 6.97 (d, *J* = 8.5 Hz, 6H, Ar), 7.11 (t, *J* = 7.4 Hz, 3H, Ar), 7.19 (t, *J* = 7.5 Hz, 3H, Ar), 7.22–7.25 (m, 6H, Ar, NHCOCH_2_NH), 7.36 (q, *J* = 6.9 Hz, 2H, Ar), 7.55 (m, 5H, Ar), 7.72 (dd, *J* = 7.6, 3.2 Hz, 2H, Ar), 7.93 (d, *J* = 8.2 Hz, 6H, Ar), 9.09 (br s, 3H, NH-1^*i*^Trp). ^13^C NMR (101 MHz, CDCl_3_) δ: 26.5 (β-CH_2_Trp), 29.3 (CH_2_CH_2_CO), 29.9 (CH_2_CH_2_CO), 37.8 (α-CH_2_Gly), 46.0 (COOCH_2_CH), 51.6 (COOCH_3_), 51.7 (α-CHTrp), 56.7 (C(NHCOCH_2_NH)), 66.2 (COOCH_2_CH), 110.7, 117.9, 118.3, 119.0, 119.5, 119.6, 123.2, 123.3, 124.0, 124.1, 124.7, 126.1, 126.5, 126.8, 136.3, 140.2, 142.6, 142.8, 144.6, 145.4 (Ar), 155.9 (COOCH_2_CH), 171.1 (COOCH_3_), 172.1 (CH_2_CH_2_CO). HRMS (ESI^+^) *m*/*z*: calcd for C_81_H_75_N_11_O_18_S_3_ 1585.4454; found 1585.4458. An alternative synthesis of this compound will be later described following the strategy described in Scheme 3.

#### Trimer **2**

Following the general procedure A, a mixture containing **8** (46 mg, 0.03 mmol) in THF (0.6 mL) and LiOH·H_2_O (8 mg, 0.17 mmol) in water (0.2 mL) was stirred at room temperature overnight. After workup, the residue was precipitated with cool diethyl ether to afford 38 mg (quantitative yield) of trimer **2** as a yellow solid. Mp (decomp at 195 °C). ^1^H NMR (400 MHz, DMSO-*d*_6_) δ: 1.70 (m, 6H, CH_2_CH_2_CO), 1.97 (m, 6H, CH_2_CH_2_CO), 3.12 (dd, *J* = 14.0, 7.3 Hz, 3H, β-CH_2_Trp), 3.25 (dd, *J* = 14.1, 6.9 Hz, 3H, β-CH_2_Trp), 3.45 (s, 2H, α-CH_2_Gly), 4.47 (m, 3H, α-CHTrp), 7.06 (t, *J* = 7.5 Hz, 3H, Ar), 7.12–7.23 (m, 9H, Ar), 7.33 (d, *J* = 8.2 Hz, 3H, Ar), 7.66 (br s, 1H, NHCOCH_2_NH_2_), 7.72 (d, *J* = 8.0 Hz, 3H, Ar), 8.09 (d, *J* = 9.0 Hz, 6H, Ar), 8.13 (d, *J* = 7.9 Hz, 3H, NH-Trp), 11.64 (br s, 3H, NH-1^*i*^Trp). ^13^C NMR (101 MHz, DMSO-*d*_6_) δ: 27.9 (β-CH_2_Trp), 29.8 (CH_2_CH_2_CO), 30.6 (CH_2_CH_2_CO), 40.9 (α-CH_2_Gly), 53.8 (α-CHTrp), 57.9 (C(NHCOCH_2_NH_2_)), 112.1, 119.8, 119.9, 120.1, 120.2, 123.8, 124.8, 126.3, 127.9, 138.0, 145.5, 148.1 (Ar), 165.6 (NHCOCH_2_NH_2_), 172.2 (CH_2_CH_2_CO), 173.6 (COOH). HRMS (ESI^+^) *m*/*z*: calcd for C_63_H_59_N_11_O_16_S_3_ 1321.3303; found 1321.3298.

#### Trimer **3**

Following the general procedure A, a mixture of compound **9**(22) (42 mg, 0.03 mmol) in THF (0.6 mL) and LiOH·H_2_O (8 mg, 0.19 mmol) in water (0.2 mL) was stirred at room temperature overnight. After workup, the residue was precipitated with cool diethyl ether to afford 39 mg (95%) of **3** as an orange solid. Mp 160–163 °C. ^1^H NMR (400 MHz, DMSO-*d*_6_) δ: 1.89 (m, 12H, CH_2_CH_2_CO), 3.10 (dd, *J* = 14.1, 7.6 Hz, 3H, β-CH_2_Trp), 3.23 (dd, *J* = 14.1, 6.4 Hz, 3H, β-CH_2_Trp), 4.46 (m, 3H, α-CHTrp), 7.04 (t, *J* = 7.5, 3H, Ar), 7.17 (m, 9H, Ar), 7.31 (d, *J* = 8.1 Hz, 3H, Ar), 7.68 (d, *J* = 8.0 Hz, 3H, Ar), 8.07 (d, *J* = 9.0 Hz, 6H, Ar), 8.29 (d, *J* = 8.1 Hz, 3H, NH-Trp), 11.62 (s, 3H, NH-1^*i*^Trp), 12.63 (br s, 3H, COOH). ^13^C NMR (101 MHz, DMSO-*d*_6_) δ: 27.2 (β-CH_2_Trp), 29.3 (CH_2_CH_2_CO), 30.5 (CH_2_CH_2_CO), 53.0 (α-CHTrp), 92.9, 111.6, 119.1, 119.4, 119.6, 119.7, 123.3, 124.3, 125.8, 127.3, 137.5, 145.0, 147.5 (Ar), 170.4 (CH_2_CH_2_CO), 172.8 (COOH). HRMS (ESI^+^) *m*/*z:* calcd for C_61_H_54_N_10_O_17_S_3_ 1294, 2831; found 1294.2830.

#### Methyl 2-((((9*H*-Fluoren-9-yl)methoxy)carbonyl)amino)-3-(2-((4-nitrophenyl)thio)-1*H*-indol-3-yl)propanoate (**11**)

Following the general procedure C, compound **10**(29) (800 mg, 1.82 mmol), 4-nitrophenyldisulfide (467 mg, 1.51 mmol), and I_2_ (230 mg, 0.91 mmol) in acetonitrile (6.0 mL) reacted for 3 h. After workup, the crude product was subjected to column chromatography (DCM/ethyl acetate, 60:1) to yield 672 mg (75%) of **11** as a yellow solid. Mp 100–102 °C. MS (ES, positive mode): *m*/*z* 594 (M + H)^+^. ^1^H NMR (400 MHz, DMSO-*d*_6_) δ: 3.19 (dd, *J* = 14.2, 8.3 Hz, 1H, β-CH_2_Trp), 3.28 (m, 1H, β-CH_2_Trp), 3.49 (s, 3H, COOCH_3_), 4.09–4.20 (m, 3H, COOCH_2_CH), 4.27 (m, 1H, α-CHTrp), 7.08 (td, *J* = 8.0, 7.0, 1.0 Hz, 1H, Ar), 7.19 (d, *J* = 8.9 Hz, 2H, Ar), 7.23 (m, 1H, Ar), 7.28 (dd, *J* = 7.4, 1.2 Hz, 1H, Ar), 7.31 (dt, *J* = 7.5, 1.2 Hz, 1H, Ar), 7.39 (m, 3H, Ar), 7.62 (dd, 2H, Ar), 7.70 (d, *J* = 8.0 Hz, 1H, Ar), 7.88 (dd, *J* = 7.6, 1.1 Hz, 2H, Ar), 7.92 (d, *J* = 8.2 Hz, 1H, NH-Trp), 8.10 (d, *J* = 8.9 Hz, 2H, Ar), 11.70 (br s, 1H, NH-1^*i*^Trp).

#### Methyl 2-((((9*H*-Fluoren-9-yl)methoxy)carbonyl)amino)-3-(2-((2-nitrophenyl)thio)-1*H*-indol-3-yl)propanoate (**12**)

Following the general procedure C, compound **10**(29) (800 mg, 1.82 mmol), 2-nitrophenyldisulfide (671 mg, 2.18 mmol), and I_2_ (276 mg, 1.09 mmol) in acetonitrile (7.3 mL) reacted for 5 h. After workup, the crude product was subjected to column chromatography (DCM/ethyl acetate, 95:5) to yield 378 mg (35%) of **12** as a yellow solid. Mp 113–115 °C. MS (ES, positive mode): *m*/*z* 594 (M + H)^+^. ^1^H NMR (400 MHz, DMSO-*d*_6_) δ: 3.20 (dd, *J* = 14.2, 8.4 Hz, 1H, β-CH_2_Trp), 3.29 (d, *J* = 6.5 Hz, 1H, β-CH_2_Trp), 3.50 (s, 3H, COOCH_3_), 4.09–4.20 (m, 3H, COOCH_2_CH), 4.28 (m, 1H, α-CHTrp), 6.70 (dd, *J* = 8.2, 1.3 Hz, 1H, Ar), 7.08 (t, *J* = 7.7 Hz, 1H, Ar), 7.21 (td, *J* = 8.2, 7.0, 1.1 Hz, 1H, Ar), 7.25–7.33 (m, 2H, Ar), 7.33–7.43 (m, 4H, Ar), 7.52 (t, 1H, Ar), 7.63 (dd, *J* = 11.8, 7.5 Hz, 2H, Ar), 7.71 (d, *J* = 8.0 Hz, 1H, Ar), 7.88 (d, *J* = 7.6 Hz, 2H, Ar), 7.91 (d, *J* = 8.2 Hz, 1H, NH-Trp), 8.27 (dd, *J* = 8.2, 1.5 Hz, 1H, Ar), 11.63 (br s, 1H, NH-1^*i*^Trp).

#### Methyl 2-((((9*H*-Fluoren-9-yl)methoxy)carbonyl)amino)-3-(2-((3-nitrophenyl)thio)-1*H*-indol-3-yl)propanoate (**13**)

Following the general procedure C, compound **10**(29) (430 mg, 0.97 mmol), 3-nitrophenyldisulfide (200 mg, 0.65 mmol), and I_2_ (168 mg, 0.66 mmol) in acetonitrile (2.6 mL) reacted for 4 h. After workup, the crude product was subjected to column chromatography (DCM/ethyl acetate, 40:1) to yield 310 mg (80%) of **13** as a yellow solid. Mp 121–123 °C. MS (ES, positive mode): *m*/*z* 594 (M + H)^+^. ^1^H NMR (400 MHz, DMSO-*d*_6_) δ: 3.20 (dd, *J* = 14.2, 8.3 Hz, 1H, β-CH_2_Trp), 3.28 (m, 1H, β-CH_2_Trp), 3.48 (s, 3H, COOCH_3_), 4.09–4.19 (m, 3H, COOCH_2_CH), 4.24 (m, 1H, α-CHTrp), 7.07 (t, *J* = 7.5 Hz, 1H, Ar), 7.21 (t, *J* = 7.3 Hz, 1H, Ar), 7.25–7.32 (m, 2H, Ar), 7.36 (d, *J* = 8.2 Hz, 1H, Ar), 7.38–7.44 (m, 3H, Ar), 7.48 (dt, *J* = 8.0, 1.3 Hz, 1H, Ar), 7.56 (t, *J* = 8.0 Hz, 1H, Ar), 7.63 (t, *J* = 6.9 Hz, 2H, Ar), 7.70 (d, *J* = 8.0 Hz, 1H, Ar), 7.79 (t, *J* = 2.1 Hz, 1H, Ar), 7.88 (d, *J* = 7.5 Hz, 2H, Ar), 7.91 (d, *J* = 8.2 Hz, 1H, Ar), 7.99 (dd, *J* = 8.1, 1.5 Hz, 1H, Ar), 11.67 (br s, 1H, NH-1^*i*^Trp).

#### Methyl 2-((((9*H*-Fluoren-9-yl)methoxy)carbonyl)amino)-3-(2-((4-(trifluoromethyl)phenyl)thio)-1*H*-indol-3-yl)propanoate (**14**)

Following the general procedure C, compound **10**(29) (282 mg, 0.64 mmol), 4-trifluoromethylphenyldisulfide (170 mg, 0.48 mmol), and I_2_ (73 mg, 0.29 mmol) in acetonitrile (1.9 mL) reacted for 3 h. After workup, the crude product was purified by CCTLC (hexane/ethyl acetate, 3:1) to yield 245 mg (83%) of **14** as a pale yellow solid. Mp 85–87 °C. MS (ES, positive mode): *m*/*z* 617 (M + H)^+^. ^1^H NMR (400 MHz, DMSO-*d*_6_) δ: 3.19 (dd, *J* = 14.2, 8.2 Hz, 1H, β-CH_2_Trp), 3.28 (m, 1H, β-CH_2_Trp), 3.49 (s, 3H, COOCH_3_), 4.10–4.21 (m, 3H, COOCH_2_CH), 4.27 (m, 1H, α-CHTrp), 7.06 (ddd, *J* = 8.0, 6.9, 1.0 Hz, 1H, Ar), 7.15–7.22 (m, 3H, Ar), 7.24–7.44 (m, 5H, Ar), 7.60 (d, *J* = 8.3 Hz, 2H, Ar), 7.62–7.71 (m, 3H, Ar), 7.88 (d, *J* = 7.7 Hz, 2H, Ar), 7.93 (d, *J* = 8.1 Hz, 1H, NH-Trp), 11.62 (br s, 1H, NH-1^*i*^Trp).

#### Methyl 2-((((9*H*-Fluoren-9-yl)methoxy)carbonyl)amino)-3-(2-((4-cyanophenyl)thio)-1*H*-indol-3-yl)propanoate (**15**)

Following the general procedure C, compound **10**(29) (431 mg, 0.98 mmol), 4-cyanophenyldisulfide (175 mg, 0.65 mmol), and I_2_ (99 mg, 0.39 mmol) in acetonitrile (2.6 mL) reacted for 4 h. After workup, the crude product was subjected to column chromatography (DCM/ethyl acetate, 70:1) to yield 206 mg (55%) of **15** as a white solid. Mp 102–104 °C. MS (ES, positive mode): *m*/*z* 574 (M + H)^+^. ^1^H NMR (400 MHz, DMSO-*d*_6_) δ: 3.18 (dd, *J* = 14.1, 8.2 Hz, 1H, β-CH_2_Trp), 3.28 (dd, *J* = 14.1, 6.8 Hz, 1H, β-CH_2_Trp), 3.48 (s, 3H, COOCH_3_), 4.10–4.21 (m, 3H, COOCH_2_CH), 4.26 (m, 1H, α-CHTrp), 7.07 (t, *J* = 7.5 Hz, 1H, Ar), 7.12 (d, *J* = 8.6 Hz, 2H, Ar), 7.20 (ddd, *J* = 8.2, 7.0, 1.1 Hz, 1H, Ar), 7.29 (m, 2H, Ar), 7.35 (d, *J* = 8.2 Hz, 1H, Ar), 7.41 (td, *J* = 7.5, 3.4 Hz, 2H, Ar), 7.64 (t, *J* = 8.8 Hz, 2H, Ar), 7.66–7.73 (m, 3H, Ar), 7.88 (d, *J* = 7.6 Hz, 2H, Ar), 7.92 (d, *J* = 8.1 Hz, 1H, NH-Trp), 11.65 (br s, 1H, NH-1^*i*^Trp).

#### Methyl 2-((((9*H*-Fluoren-9-yl)methoxy)carbonyl)amino)-3-(2-((4-fluorophenyl)thio)-1*H-*indol-3-yl)propanoate (**16**)

Following the general procedure C, compound **10**(29) (538 mg, 1.22 mmol), disulfide **20a** (207 mg, 0.81 mmol), and I_2_ (124 mg, 0.49 mmol) in acetonitrile (3.2 mL) reacted for 6 h. After workup, the crude product was subjected to column chromatography (DCM/ethyl acetate, 70:1) to yield 311 mg (72%) of **16** as a white solid. Mp 76–78 °C. MS (ES, positive mode): *m*/*z* 567 (M + H)^+^. ^1^H NMR (400 MHz, DMSO-*d*_6_) δ: 3.18 (dd, *J* = 14.1, 8.1 Hz, 1H, β-CH_2_Trp), 3.29 (m, 1H, β-CH_2_Trp), 3.49 (s, 3H, COOCH_3_), 4.12–4.22 (m, 3H, COOCH_2_CH), 4.25 (m, 1H, α-CHTrp), 7.03 (t, *J* = 7.5 Hz, 1H, Ar), 7.13 (d, *J* = 6.9 Hz, 4H, Ar), 7.16 (m, 1H, Ar), 7.25–7.35 (m, 3H, Ar), 7.41 (m, 2H, Ar), 7.65 (m, 3H, Ar), 7.88 (d, *J* = 7.8 Hz, 2H, Ar), 7.92 (d, *J* = 8.1 Hz, 1H, NH-Trp), 11.51 (br s, 1H, NH-1^*i*^Trp).

#### Methyl 2-((((9*H*-Fluoren-9-yl)methoxy)carbonyl)amino)-3-(2-((4-acetylphenyl)thio)-1*H*-indol-3-yl)propanoate (**17**)

Following the general procedure C, compound **10**(29) (486 mg, 1.10 mmol), disulfide **20b** (371 mg, 1.23 mmol), and I_2_ (187 mg, 0.74 mmol) in acetonitrile (4.4 mL) reacted for 2.5 h. After workup, the crude product was subjected to column chromatography (hexane/ethyl acetate, 2:1) to yield 382 mg (59%) of **17** as a pale yellow solid. Mp 100–102 °C. MS (ES, positive mode): *m*/*z* 591 (M + H)^+^. ^1^H NMR (400 MHz, DMSO-*d*_6_) δ: 2.47 (s, 3H, COCH_3_), 3.18 (dd, *J* = 14.2, 8.2 Hz, 1H, β-CH_2_Trp), 3.28 (dd, *J* = 14.3, 7.0 Hz, 1H, β-CH_2_Trp), 3.48 (s, 3H, COOCH_3_), 4.10–4.20 (m, 3H, COOCH_2_CH), 4.25 (m, 1H, α-CHTrp), 7.05 (t, *J* = 7.5 Hz, 1H, Ar), 7.09 (d, *J* = 8.5 Hz, 2H, Ar), 7.19 (t, *J* = 7.7 Hz, 1H, Ar), 7.25–7.36 (m, 3H, Ar), 7.39 (m, 2H, Ar), 7.63 (t, *J* = 8.5 Hz, 2H, Ar), 7.68 (d, *J* = 8.0 Hz, 1H, Ar), 7.82 (d, *J* = 8.3 Hz, 2H, Ar), 7.88 (d, *J* = 7.6 Hz, 2H, Ar), 7.96 (d, *J* = 8.1 Hz, 1H, NH-Trp), 11.64 (br s, 1H, NH-1^*i*^Trp).

#### Methyl 2-((((9*H*-Fluoren-9-yl)methoxy)carbonyl)amino)-3-(2-((4-(methylsulfonyl)phenyl)thio)-1*H*-indol-3-yl)propanoate (**18**)

Following the general procedure C, compound **10**(29) (329 mg, 0.75 mmol), disulfide **20c** (281 mg, 0.75 mmol), and I_2_ (114 mg, 0.45 mmol) in acetonitrile (3.0 mL) reacted for 4 h. After workup, the crude product was subjected to column chromatography (hexane/ethyl acetate, 2:1) to yield 336 mg (71%) of **18** as a white solid. Mp 119–121 °C. MS (ES, positive mode): *m*/*z* 627 (M + H)^+^. ^1^H NMR (400 MHz, DMSO-*d*_6_) δ: 3.14 (s, 3H, SO_2_CH_3_), 3.18 (dd, *J* = 14.2, 9.0 Hz, 1H, β-CH_2_Trp), 3.29 (dd, *J* = 14.1, 6.8 Hz, 1H, β-CH_2_Trp), 3.49 (s, 3H, COOCH_3_), 4.11–4.21 (m, 3H, COOCH_2_CH), 4.26 (m, 1H, α-CHTrp), 7.06 (t, *J* = 7.6 Hz, 1H, Ar), 7.17–7.24 (m, 3H, Ar), 7.29 (m, 2H, Ar), 7.35 (d, *J* = 8.2 Hz, 1H, Ar), 7.41 (td, *J* = 7.5, 3.7 Hz, 2H, Ar), 7.64 (dd, *J* = 10.5, 7.5 Hz, 2H, Ar), 7.69 (d, *J* = 8.0 Hz, 1H, Ar), 7.78 (d, *J* = 8.6 Hz, 2H, Ar), 7.88 (d, *J* = 7.6 Hz, 2H, Ar), 7.97 (d, *J* = 8.1 Hz, 1H, NH-Trp), 11.66 (br s, 1H, NH-1^*i*^Trp).

#### Methyl 2-Amino-3-(2-((4-nitrophenyl)thio)-1*H*-indol-3-yl)propanoate (**21**)

Following the general procedure E, monomer **11** (460 mg, 0.77 mmol) and piperidine (765 μL, 7.75 mmol) in DCM (8.0 mL) reacted for 2 h. After workup, the residue was subjected to column chromatography (DCM/hexane/methanol, 10:10:0.6) to yield 244 mg (85%) of **21** as a yellow solid. Mp 73–75 °C. MS (ES, positive mode): *m*/*z* 372 (M + H)^+^. ^1^H NMR (400 MHz, DMSO-*d*_6_) δ: 2.98 (dd, *J* = 13.9, 7.0 Hz, 1H, β-CH_2_Trp), 3.12 (dd, *J* = 13.8, 6.9 Hz, 1H, β-CH_2_Trp), 3.47 (s, 3H, COOCH_3_), 3.57 (t, *J* = 6.9 Hz, 1H, α-CHTrp), 7.09 (t, *J* = 5.0 Hz1H, Ar), 7.17–7.23 (m, 3H, Ar), 7.36 (d, *J* = 8.3 Hz, 1H, Ar), 7.63 (d, *J* = 8.0 Hz, 1H, Ar), 8.13 (d, *J* = 9.0 Hz, 2H, Ar), 11.65 (br s, 1H, NH-1^*i*^Trp).

#### Methyl 2-Amino-3-(2-((2-nitrophenyl)thio)-1*H*-indol-3-yl)propanoate (**22**)

Following the general procedure E, monomer **12** (370 mg, 0.62 mmol) and piperidine (616 μL, 6.23 mmol) in DCM (6.2 mL) reacted for 2 h. After workup, the residue was subjected to column chromatography (DCM/hexane/methanol, 25:25:1) to yield 166 mg (72%) of **22** as a yellow solid. Mp 77–79 °C. MS (ES, positive mode): *m*/*z* 372 (M + H)^+^. ^1^H NMR (400 MHz, DMSO-*d*_6_) δ: 3.00 (dd, *J* = 13.9, 6.9 Hz, 1H, β-CH_2_Trp), 3.14 (dd, *J* = 13.9, 7.0 Hz, 1H, β-CH_2_Trp), 3.47 (s, 3H, COOCH_3_), 3.58 (t, *J* = 6.9 Hz, 1H, α-CHTrp), 6.71 (dd, *J* = 8.2, 1.3 Hz, 1H, Ar), 7.08 (t, *J* = 7.5 Hz, 1H, Ar), 7.21 (t, *J* = 7.6 Hz, 1H, Ar), 7.34 (d, *J* = 8.1 Hz, 1H, Ar), 7.40 (td, *J* = 8.4, 7.1, 1.3 Hz, 1H, Ar), 7.57 (td, *J* = 8.4, 7.2, 1.5 Hz, 1H, Ar), 7.64 (d, *J* = 8.0 Hz, 1H, Ar), 8.28 (dd, *J* = 8.2, 1.5 Hz, 1H, Ar), 11.56 (br s, 1H, NH-1^*i*^Trp).

#### Methyl 2-Amino-3-(2-((3-nitrophenyl)thio)-1*H-*indol-3-yl)propanoate (**23**)

Following the general procedure E, monomer **13** (277 mg, 0.47 mmol) and piperidine (461 μL, 4.67 mmol) in DCM (4.7 mL) reacted for 1.5 h. After workup, the residue was purified by CCTLC (DCM/hexane/methanol, 10:10:0.7) to yield 133 mg (89%) of **23** as a yellow solid. Mp 80–82 °C. MS (ES, positive mode): *m*/*z* 372 (M + H)^+^. ^1^H NMR (400 MHz, DMSO-*d*_6_) δ: 3.01 (dd, *J* = 13.8, 7.0 Hz, 1H, β-CH_2_Trp), 3.14 (dd, *J* = 13.8, 7.0 Hz, 1H, β-CH_2_Trp), 3.44 (s, 3H, COOCH_3_), 3.56 (t, *J* = 7.0 Hz, 1H, α-CHTrp), 7.08 (t, 1H, Ar), 7.20 (ddd, *J* = 8.2, 6.9, 1.1 Hz, 1H, Ar), 7.35 (d, *J* = 8.2 Hz, 1H, Ar), 7.49 (dt, *J* = 8.0, 1.4 Hz, 1H, Ar), 7.58 (t, *J* = 8.1 Hz, 1H, Ar), 7.62 (d, *J* = 8.2 Hz, 1H, Ar), 7.81 (t, *J* = 2.0 Hz, 1H, Ar), 8.00 (dd, *J* = 7.8, 2.1 Hz, 1H, Ar), 11.61 (br s, 1H, NH-1^*i*^Trp).

#### Methyl 2-Amino-3-(2-((4-(trifluoromethyl)phenyl)thio)-1*H-*indol-3-yl)propanoate (**24**)

Following the general procedure E, monomer **14** (237 mg, 0.38 mmol) and piperidine (379 μL, 3.84 mmol) in DCM (3.8 mL) reacted for 2 h. After workup, the residue was purified by CCTLC (DCM/hexane/methanol, 10:10:0.5) to yield 103 mg (68%) of **24** as an amorphous pale yellow solid. MS (ES, positive mode): *m*/*z* 395 (M + H)^+^. ^1^H NMR (400 MHz, DMSO-*d*_6_) δ: 2.99 (dd, *J* = 13.8, 7.0 Hz, 1H, β-CH_2_Trp), 3.13 (dd, *J* = 13.8, 6.9 Hz, 1H, β-CH_2_Trp), 3.46 (s, 3H, COOCH_3_), 3.57 (t, *J* = 6.9 Hz, 1H, α-CHTrp), 7.07 (ddd, *J* = 8.1, 6.9, 1.1 Hz, 1H, Ar), 7.16–7.22 (m, 3H, Ar), 7.34 (d, *J* = 8.2 Hz, 1H, Ar), 7.59–7.66 (m, 3H, Ar), 11.56 (br s, 1H, NH-1^*i*^Trp).

#### Methyl 2-Amino-3-(2-((4-cyanophenyl)thio)-1*H*-indol-3-yl)propanoate (**25**)

Following the general procedure E, monomer **15** (196 mg, 0.34 mmol) and piperidine (337 μL, 3.42 mmol) in DCM (3.4 mL) reacted for 3 h. After workup, the residue was purified by CCTLC (DCM/hexane/methanol, 10:10:1.4) to yield 98 mg (82%) of **25** as a pale yellow solid. Mp 72–74 °C. MS (ES, positive mode): *m*/*z* 352 (M + H)^+^. ^1^H NMR (500 MHz, DMSO-*d*_6_) δ: 2.97 (dd, *J* = 13.9, 7.0 Hz, 1H, β-CH_2_Trp), 3.11 (dd, *J* = 13.9, 7.0 Hz, 1H, β-CH_2_Trp), 3.46 (s, 3H, COOCH_3_), 3.56 (t, *J* = 6.9 Hz, 1H, α-CHTrp), 7.07 (ddd, *J* = 8.1, 7.0, 1.1 Hz, 1H, Ar), 7.13 (m, 2H, Ar), 7.20 (ddd, *J* = 8.2, 7.0, 1.2 Hz, 1H, Ar), 7.34 (dd, *J* = 8.2, 1.0 Hz, 1H, Ar), 7.62 (dd, *J* = 8.1, 1.1 Hz, 1H, Ar), 7.72 (m, 2H, Ar), 11.60 (br s, 1H, NH-1^*i*^Trp).

#### Methyl 2-Amino-3-(2-((4-fluorophenyl)thio)-1*H*-indol-3-yl)propanoate (**26**)

Following the general procedure E, monomer **16** (303 mg, 0.53 mmol) and piperidine (528 μL, 5.35 mmol) in DCM (5.3 mL) reacted for 2 h. After workup, the residue was purified by CCTLC (DCM/hexane/methanol, 10:10:0.6) to yield 140 mg (76%) of **26** as an amorphous white solid. MS (ES, positive mode): *m*/*z* 345 (M + H)^+^. ^1^H NMR (400 MHz, DMSO-*d*_6_) δ: 3.00 (dd, *J* = 13.8, 7.0 Hz, 1H, β-CH_2_Trp), 3.13 (dd, *J* = 13.8, 6.9 Hz, 1H, β-CH_2_Trp), 3.48 (s, 3H, COOCH_3_), 3.57 (t, *J* = 6.9 Hz, 1H, α-CHTrp), 7.03 (ddd, *J* = 8.1, 7.0, 1.1 Hz, 1H, Ar), 7.07–7.20 (m, 5H, Ar), 7.31 (dd, *J* = 8.1, 1.0 Hz, 1H, Ar), 7.57 (d, *J* = 7.9 Hz, 1H, Ar), 11.45 (br s, 1H, NH-1^*i*^Trp).

#### Methyl 3-(2-((4-Acetylphenyl)thio)-1*H*-indol-3-yl)-2-aminopropanoate (**27**)

Following the general procedure E, monomer **17** (451 mg, 0.76 mmol) and piperidine (787 μL, 7.63 mmol) in DCM (7.6 mL) reacted for 3 h. After workup, the residue was purified by CCTLC (DCM/methanol, 30:1) to yield 203 mg (83%) of **27** as a pale yellow solid. Mp 79–81 °C. MS (ES, positive mode): *m*/*z* 369 (M + H)^+^. ^1^H NMR (400 MHz, DMSO-*d*_6_) δ: 2.99 (dd, *J* = 13.8, 7.0 Hz, 1H, β-CH_2_Trp), 3.12 (dd, *J* = 13.8, 6.9 Hz, 1H, β-CH_2_Trp), 3.46 (s, 3H, COOCH_3_), 3.57 (t, *J* = 6.9 Hz, 1H, α-CHTrp), 7.01–7.13 (m, 3H, Ar), 7.19 (ddd, *J* = 8.2, 7.0, 1.2 Hz, 1H, Ar), 7.34 (d, 1H, Ar), 7.61 (d, *J* = 8.0 Hz, 1H, Ar), 7.84 (dd, *J* = 9.3, 2.7 Hz, 2H, Ar).

#### Methyl 2-Amino-3-(2-((4-(methylsulfonyl)phenyl)thio)-1*H*-indol-3-yl)propanoate (**28**)

Following the general procedure E, monomer **18** (297 mg, 0.47 mmol) and piperidine (488 μL, 4.74 mmol) in DCM (4.7 mL) reacted for 3 h. After workup, the residue was purified by CCTLC (DCM/methanol, 40:1) to yield 170 mg (89%) of **28** as a white solid. Mp 64–66 °C. MS (ES, positive mode): *m*/*z* 405 (M + H)^+^. ^1^H NMR (400 MHz, DMSO-*d*_6_) δ: 2.99 (dd, *J* = 13.8, 7.0 Hz, 1H, β-CH_2_Trp), 3.12 (m, 1H, β-CH_2_Trp), 3.16 (s, 3H, SO_2_CH_3_), 3.47 (s, 3H, COOCH_3_), 3.58 (t, *J* = 6.9 Hz, 1H, α-CHTrp), 7.07 (ddd, *J* = 8.1, 6.9, 1.1 Hz, 1H, Ar), 7.17–7.24 (m, 3H), 7.34 (d, *J* = 8.2 Hz, 1H, Ar), 7.62 (d, *J* = 8.0 Hz, 1H, Ar), 7.79 (d, *J* = 8.6 Hz, 2H, Ar), 11.58 (br s, 1H, NH-1^*i*^Trp).

#### Trimer **8**

(Obtained by coupling the tripodal acid **6**^27^ and the monomer **21**). Following the general procedure B, a solution containing the tripodal acid **6**(27) (105 mg, 0.20 mmol), monomer **21** (244 mg, 0.66 mmol), HATU (303 mg, 0.80 mmol), and DIPEA (242 μL, 1.39 mmol) in anhydrous DMF (2.0 mL) reacted for 24 h. After workup, the residue was purified by CCTLC (DCM/methanol, 25:1) to yield 282 mg (89%) of **8** as a yellow solid. The analytical and spectroscopic data have been described under the heading trimer **8**.

#### Trimer **29**

Following the general procedure B, a solution containing the tripodal acid **6**(27) (40 mg, 0.08 mmol), monomer **22** (102 mg, 0.27 mmol), HATU (116 mg, 0.30 mmol), and DIPEA (93 μL, 0.53 mmol) in anhydrous DMF (0.8 mL) reacted for 24 h. After workup, the residue was purified by CCTLC (DCM/methanol, 10:1) to yield 92 mg (76%) of **29** as a yellow solid. Mp (decomp at 116 °C). ^1^H NMR (400 MHz, CDCl_3_) δ: 1.71 (m, 6H, CH_2_CH_2_CO), 1.99 (m, 6H, CH_2_CH_2_CO), 2.79 (s, 2H, α-CH_2_Gly), 3.22 (dd, *J* = 14.3, 7.4 Hz, 3H, β-CH_2_Trp), 3.39 (dd, *J* = 14.5, 5.1 Hz, 3H, β-CH_2_Trp), 3.64 (s, 9H, COOCH_3_), 4.13 (t, *J* = 7.4 Hz, 1H, COOCH_2_CH), 4.25 (d, *J* = 7.3 Hz, 2H, COOCH_2_CH), 4.89 (m, 3H, α-CHTrp), 5.54 (br s, 1H, NHCOCH_2_NH), 6.63 (m, 6H, NH-Trp, Ar), 7.12 (t, *J* = 7.5 Hz, 3H, Ar), 7.19 (m, 16H, Ar, NHCOCH_2_NH), 7.34 (t, *J* = 7.5 Hz, 2H, Ar), 7.55 (d, *J* = 7.4 Hz, 2H, Ar), 7.60 (d, *J* = 8.0 Hz, 3H, Ar), 7.70 (d, *J* = 7.5 Hz, 2H, Ar), 8.12 (d, *J* = 8.2 Hz, 3H, Ar), 9.04 (br s, 3H, NH-1^*i*^Trp). ^13^C NMR (101 MHz, CDCl_3_) δ: 26.2 (β-CH_2_Trp), 29.3 (CH_2_CH_2_CO), 29.9 (CH_2_CH_2_CO), 37.7 (α-CH_2_Gly), 46.0 (COOCH_2_CH), 51.6 (COOCH_3_), 51.7 (α-CHTrp), 57.1 (C(NHCOCH_2_NH)), 66.1 (COOCH_2_CH), 110.8, 118.4, 118.8, 119.3, 120.8, 123.2, 124.2, 124.6, 124.9, 126.1, 126.6, 126.7, 126.9, 133.2, 136.6, 136.8, 140.2, 142.8, 142.9, 143.4 (Ar), 155.8 (COOCH_2_CH), 170.9 (COOCH_3_), 172.4 (CH_2_CH_2_CO). HRMS (ESI^+^) *m*/*z*: calcd for C_81_H_75_N_11_O_18_S_3_ 1585.4454; found 1585.4455.

#### Trimer **30**

Following the general procedure B, a solution containing the tripodal acid **6**(27) (55 mg, 0.11 mmol), monomer **23** (129 mg, 0.35 mmol), HATU (160 mg, 0.42 mmol), and DIPEA (128 μL, 0.74 mmol) in anhydrous DMF (1.0 mL) reacted for 24 h. After workup, the residue was purified by CCTLC (DCM/methanol, 44:1) to yield 87 mg (52%) of **30** as a yellow solid. Mp (decomp at 124 °C). ^1^H NMR (400 MHz, CDCl_3_) δ: 1.50 (m, 3H, CH_2_CH_2_CO), 1.69 (s, 3H, CH_2_CH_2_CO), 1.95 (s, 6H, CH_2_CH_2_CO), 3.22 (dd, *J* = 14.3, 8.0 Hz, 3H, β-CH_2_Trp), 3.42 (dd, *J* = 14.3, 5.4 Hz, 3H, β-CH_2_Trp), 3.55 (m, 2H, α-CH_2_Gly), 3.65 (s, 9H, COOCH_3_), 4.15 (t, *J* = 7.2 Hz, 1H, COOCH_2_CH), 4.29 (m, 2H, COOCH_2_CH), 4.88 (m, 3H, α-CHTrp), 5.47 (br s, 1H, NHCOCH_2_NH), 6.71 (br s, 3H, NH-Trp), 7.09 (t, *J* = 7.4 Hz, 3H, Ar), 7.15–7.25 (m, 14H, Ar), 7.36 (t, *J* = 7.3 Hz, 2H, Ar), 7.52–7.61 (m, 5H, Ar), 7.72 (d, *J* = 7.5 Hz, 2H, Ar), 7.84–7.90 (m, 6H), 9.06 (br s, 3H, NH-1^*i*^Trp). HRMS (ESI^+^) *m*/*z*: calcd for C_81_H_75_N_11_O_18_S_3_ 1585.4454; found 1585.4466.

#### Trimer **31**

Following the general procedure B, a solution containing the tripodal acid **6**(27) (34 mg, 0.06 mmol), monomer **24** (92 mg, 0.23 mmol), HATU (99 mg, 0.26 mmol), and DIPEA (80 μL, 0.46 mmol) in anhydrous DMF (0.7 mL) reacted for 24 h. After workup, the residue was purified by CCTLC (DCM/methanol, 25:1) to yield 73 mg (68%) of **31** as a white solid. Mp (decomp at 126 °C). ^1^H NMR (400 MHz, CDCl_3_) δ: 1.54 (m, 3H, CH_2_CH_2_CO), 1.75 (m, 3H, CH_2_CH_2_CO), 1.93 (m, 6H, CH_2_CH_2_CO), 2.80 (s, 2H, α-CH_2_Gly), 3.21 (dd, *J* = 14.3, 7.6 Hz, 3H, β-CH_2_Trp), 3.39 (dd, *J* = 14.4, 5.5 Hz, 3H, β-CH_2_Trp), 3.64 (s, 9H, COOCH_3_), 4.18 (t, *J* = 7.0 Hz, 1H, COOCH_2_CH), 4.32 (m, 2H, COOCH_2_CH), 4.90 (td, *J* = 7.9, 5.4 Hz, 3H, α-CHTrp), 5.29 (br s, 1H, NHCOCH_2_NH), 6.43 (d, *J* = 8.1 Hz, 3H, NH-Trp), 7.01 (d, *J* = 8.2 Hz, 6H, Ar), 7.10 (ddd, *J* = 8.0, 6.2, 1.8 Hz, 2H, Ar), 7.14–7.22 (m, 6H, Ar), 7.23–7.25 (m, 3H, Ar, NHCOCH_2_NH), 7.37 (m, 8H, Ar), 7.52–7.62 (m, 5H, Ar), 7.74 (dd, *J* = 7.5, 2.2 Hz, 2H, Ar), 8.93 (br s, 3H, NH-1^*i*^Trp). ^13^C NMR (101 MHz, CDCl_3_) δ: 27.6 (β-CH_2_Trp), 30.5 (CH_2_CH_2_CO), 31.0 (CH_2_CH_2_CO), 38.7 (α-CH_2_Gly), 47.2 (COOCH_2_CH), 52.6 (COOCH_3_), 52.7 (α-CHTrp), 57.6 (C(NHCOCH_2_NH)), 67.2 (COOCH_2_CH), 111.6, 118.5, 119.3, 120.1, 120.4, 121.8, 124.0 (q, *J* = 271.9 Hz), 124.1, 125.2, 126.0 (q, *J* = 3.7 Hz), 126.2, 127.2, 127.7, 127.8, 128.0 (q, *J* = 31.5 Hz), 137.3, 141.4, 141.8, 143.9, 144.0 (Ar, CF_3_), 156.8 (COOCH_2_CH), 168.5 (NHCOCH_2_NH), 172.4 (COOCH_3_), 172.9 (CH_2_CH_2_CO).

#### Trimer **32**

Following the general procedure B, a solution containing the tripodal acid **6**(27) (39 mg, 0.07 mmol), monomer **25** (86 mg, 0.25 mmol), HATU (112 mg, 0.30 mmol), and DIPEA (90 μL, 0.52 mmol) in anhydrous DMF (0.8 mL) reacted for 24 h. After workup, the residue was purified by CCTLC (DCM/methanol, 22:1) to yield 74 mg (65%) of **32** as a pale yellow solid. Mp (decomp at 115 °C). ^1^H NMR (400 MHz, CDCl_3_) δ: 1.81 (m, 6H, CH_2_CH_2_CO), 1.93 (m, 6H, CH_2_CH_2_CO), 2.87 (s, 2H, α-CH_2_Gly), 3.18 (dd, *J* = 14.3, 7.8 Hz, 3H, β-CH_2_Trp), 3.37 (dd, *J* = 14.3, 5.4 Hz, 3H, β-CH_2_Trp), 3.64 (s, 9H, COOCH_3_), 4.16 (t, *J* = 7.0 Hz, 1H, COOCH_2_CH), 4.30 (m, 2H, COOCH_2_CH), 4.86 (m, 3H, α-CHTrp), 5.49 (br s, 1H, NHCOCH_2_NH), 6.47 (d, *J* = 8.2 Hz, 3H, NH-Trp), 6.96 (d, *J* = 8.2 Hz, 6H, Ar), 7.10 (t, *J* = 7.4 Hz, 3H, Ar), 7.19 (t, *J* = 7.4 Hz, 3H), 7.36 (m, 8H, Ar), 7.52–7.60 (m, 5H, Ar), 7.23–7.25 (m, 6H, Ar, NHCOCH_2_NH), 7.74 (d, *J* = 7.6 Hz, 2H, Ar), 9.18 (br s, 3H, NH-1^*i*^Trp). HRMS (ESI^+^) *m*/*z*: calcd for C_84_H_75_N_11_O_12_S_3_ 1525.4759; found 1525.4769.

#### Trimer **33**

Following the general procedure B, a solution containing the tripodal acid **6**(27) (63 mg, 0.12 mmol), monomer **26** (135 mg, 0.39 mmol), HATU (181 mg, 0.48 mmol), and DIPEA (145 μL, 0.83 mmol) in anhydrous DMF (1.2 mL) reacted for 24 h. After workup, the residue was purified by CCTLC (DCM/methanol, 28:1) to yield 133 mg (74%) of **33** as a pale yellow solid. Mp (decomp at 107 °C). ^1^H NMR (400 MHz, DMSO-*d*_6_) δ: 1.75 (m, 6H, CH_2_CH_2_CO), 1.99 (m, 6H, CH_2_CH_2_CO), 3.14 (dd, *J* = 13.9, 6.7 Hz, 3H, β-CH_2_Trp), 3.25 (dd, *J* = 14.0, 8.0 Hz, 3H, β-CH_2_Trp), 3.41 (s, 9H, COOCH_3_), 3.58 (d, *J* = 5.3 Hz, 2H, α-CH_2_Gly), 4.18 (m, 1H, COOCH_2_CH), 4.26 (d, *J* = 7.0 Hz, 2H, COOCH_2_CH), 4.46 (m, 3H, α-CHTrp), 7.03 (t, *J* = 7.3 Hz, 3H, Ar), 7.08–7.22 (m, 16H), 7.29 (dd, *J* = 7.9, 2.3 Hz, 5H), 7.38 (q, *J* = 6.8, 6.0 Hz, 3H), 7.56 (d, *J* = 8.0 Hz, 3H), 7.72–7.66 (m, 2H), 7.87 (d, *J* = 7.6 Hz, 2H), 8.29 (d, *J* = 7.5 Hz, 3H), 11.48 (s, 3H). HRMS (ESI^+^) *m*/*z*: calcd for C_81_H_75_F_3_N_8_O_12_S_3_ 1504.4619; found 1504.4647.

#### Trimer **34**

Following the general procedure B, a solution containing the tripodal acid **6**(27) (56 mg, 0.11 mmol), monomer **27** (150 mg, 0.41 mmol), HATU (161 mg, 0.42 mmol), and DIPEA (129 μL, 0.74 mmol) in anhydrous DMF (1.1 mL) reacted for 24 h. After workup, the residue was purified by CCTLC (DCM/methanol, 28:1) to yield 95 mg (57%) of **34** as a pale yellow solid. Mp (decomp at 137 °C). ^1^H NMR (400 MHz, CDCl_3_) δ: 1.54 (m, 3H, CH_2_CH_2_CO), 1.72 (m, 3H, CH_2_CH_2_CO), 1.90 (m, 6H, CH_2_CH_2_CO), 2.45 (s, 9H, COCH_3_), 3.21 (dd, *J* = 14.2, 7.6 Hz, 3H, β-CH_2_Trp), 3.38 (dd, *J* = 14.3, 5.4 Hz, 3H, β-CH_2_Trp), 3.51 (m, 2H, α-CH_2_Gly), 3.63 (s, 9H, COOCH_3_), 4.14 (t, *J* = 7.0 Hz, 1H, COOCH_2_CH), 4.30 (d, *J* = 7.0 Hz, 2H, COOCH_2_CH), 4.87 (td, *J* = 7.9, 5.5 Hz, 3H, α-CHTrp), 5.41 (br s, 1H, NHCOCH_2_NH), 6.43 (d, *J* = 8.1 Hz, 3H, NH-Trp), 6.97 (d, *J* = 8.4 Hz, 6H, Ar), 7.10 (td, *J* = 7.4, 6.8, 1.2 Hz, 3H, Ar), 7.18 (d, 3H, *J* = 7.6 Hz, Ar), 7.21–7.25 (m, 4H, Ar), 7.36 (td, *J* = 7.5, 2.3 Hz, 3H, Ar), 7.51–7.61 (m, 5H, Ar, NHCOCH_2_NH), 7.69 (d, *J* = 8.4 Hz, 6H), 7.73 (d, *J* = 7.7 Hz, 3H), 9.01 br (s, 3H, NH-1^*i*^Trp). ^13^C NMR (101 MHz, CDCl_3_) δ: 25.4 (COCH_3_), 26.4 (β-CH_2_Trp), 29.4 (CH_2_CH_2_CO), 29.8 (CH_2_CH_2_CO), 43.5 (α-CH_2_Gly), 46.1 (COOCH_2_CH), 51.5 (COOCH_3_), 51.7 (α-CHTrp), 56.5 (C(NHCOCH_2_NH)), 66.1 (COOCH_2_CH), 117.4, 118.2, 110.5, 118.9, 119.3, 120.5, 123.0, 124.1, 124.6, 126.1, 126.7, 128.0, 133.5, 136.2, 140.2, 142.6, 142.8, 142.9 (Ar), 155.7 (COOCH_2_CH), 167.4 (NHCOCH_2_NH), 171.3 (COOCH_3_), 171.7 (CH_2_CH_2_CO), 196.2 (COCH_3_). HRMS (ESI^+^) *m*/*z*: calcd for C_87_H_84_N_8_O_15_S_3_ 1576.5218; found 1576.5230.

#### Trimer **35**

Following the general procedure B, a solution containing the tripodal acid **6**(27) (64 mg, 0.12 mmol), monomer **28** (162 mg, 0.40 mmol), HATU (185 mg, 0.48 mmol), and DIPEA (147 μL, 0.85 mmol) in anhydrous DMF (1.2 mL) reacted for 24 h. After workup, the residue was purified by CCTLC (DCM/methanol, 25:1) to yield 110 mg (54%) of **35** as a white solid. Mp (decomp at 108 °C). ^1^H NMR (400 MHz, DMSO-*d*_6_) δ: 1.72 (s, 6H, CH_2_CH_2_CO), 2.00 (t, *J* = 8.6 Hz, 6H, CH_2_CH_2_CO), 3.14 (s, 12H, SO_2_CH_3_, β-CH_2_Trp), 3.24 (m, 3H, β-CH_2_Trp), 3.41 (s, 9H, COOCH_3_), 3.58 (d, *J* = 5.9 Hz, 2H, α-CH_2_Gly), 4.20 (d, *J* = 6.5 Hz, 1H, COOCH_2_CH), 4.26 (d, *J* = 7.1 Hz, 2H, COOCH_2_CH), 4.47 (m, 3H, α-CHTrp), 7.07 (t, *J* = 7.5 Hz, 3H, Ar), 7.16 (m, 3H, Ar), 7.20 (d, *J* = 8.6 Hz, 6H, Ar), 7.29 (t, *J* = 7.5 Hz, 2H, Ar), 7.33 (d, *J* = 8.2 Hz, 3H, Ar), 7.35–7.42 (m, 3H, Ar, NHCOCH_2_NH), 7.61 (d, *J* = 8.0 Hz, 3H, Ar), 7.68 (dd, *J* = 7.6, 3.8 Hz, 2H, Ar), 7.79 (d, *J* = 8.6 Hz, 6H, Ar), 7.87 (d, *J* = 7.5 Hz, 2H, Ar), 8.31 (d, *J* = 7.5 Hz, 3H, NH-Trp), 11.62 (br s, 3H, NH-1^*i*^Trp). ^13^C NMR (101 MHz, DMSO-*d*_6_) δ: 25.4 (β-CH_2_Trp), 27.5 (CH_2_CH_2_CO), 28.4 (CH_2_CH_2_CO), 41.9 (SO_2_CH_3_), 45.0 (COOCH_2_CH), 50.1 (COOCH_3_), 51.6 (α-CHTrp), 55.1 (C(NHCOCH_2_NH)), 64.0 (COOCH_2_CH), 109.9, 116.6, 117.6, 117.7, 118.4, 118.5, 121.6, 123.6, 124.2, 125.4, 125.5, 125.9, 126.2, 135.7, 136.1, 139.0, 142.2, 143.1 (Ar), 154.7 (COOCH_2_CH), 166.4 (NHCOCH_2_NH), 170.3 (COOCH_3_), 170.4 (CH_2_CH_2_CO). HRMS (ESI^+^) *m*/*z*: calcd for C_81_H_75_N_11_O_18_S_3_ 1684.4228; found 1684.4223.

#### Trimer **36**

Following the general procedure A, a mixture containing trimer **29** (45 mg, 0.03 mmol) in THF (0.6 mL) and LiOH·H_2_O (8 mg, 0.17 mmol) in water (0.2 mL) was stirred at room temperature overnight. After workup, the residue was precipitated with cool diethyl ether to afford 35 mg (94%) of **36** as a yellow solid. Mp (decomp at 166 °C). ^1^H NMR (500 MHz, DMSO-*d*_6_) δ: 1.66 (m, 6H, CH_2_CH_2_CO), 1.87 (m, 6H, CH_2_CH_2_CO), 3.13 (dd, *J* = 14.1, 7.6 Hz, 3H, β-CH_2_Trp), 3.26 (dd, *J* = 14.1, 6.9 Hz, 3H, β-CH_2_Trp), 3.44 (s, 2H, α-CH_2_Gly), 4.49 (m, 3H, α-CHTrp), 6.71 (d, *J* = 8.2 Hz, 3H, Ar), 7.06 (t, *J* = 7.5 Hz, 3H, Ar), 7.16 (t, *J* = 7.6 Hz, 3H, Ar), 7.31 (d, *J* = 8.2 Hz, 3H, Ar), 7.37 (t, *J* = 7.7 Hz, 3H, Ar), 7.51 (t, *J* = 7.5 Hz, 3H, Ar), 7.62 (br s, 1H, NHCOCH_2_NH_2_), 7.72 (d, *J* = 8.0 Hz, 3H, Ar), 8.09 (d, *J* = 8.1 Hz, 3H, NH-Trp), 8.25 (d, *J* = 8.2 Hz, 3H, Ar), 11.55 (br s, 3H, NH-1^*i*^Trp). ^13^C NMR (126 MHz, DMSO-*d*_6_) δ: 27.8 (β-CH_2_Trp), 29.6 (CH_2_CH_2_CO), 30.4 (CH_2_CH_2_CO), 40.8 (α-CH_2_Gly), 53.6 (α-CHTrp), 57.7 (C(NHCOCH_2_NH_2_)), 112.0, 119.8, 120.1, 120.4, 121.3, 123.8, 126.7, 126.6, 127.8, 128.5, 135.1, 137.7, 138.0, 144.9 (Ar), 165.4 (NHCOCH_2_NH_2_), 172.2 (CH_2_CH_2_CO), 173.6 (COOH). HRMS (ESI^+^) *m*/*z*: calcd for C_63_H_59_N_11_O_16_S_3_ 1321.3303; found 1321.3308.

#### Trimer **37**

Following the general procedure A, a mixture containing trimer **30** (75 mg, 0.05 mmol) in THF (0.9 mL) and LiOH·H_2_O (12 mg, 0.28 mmol) in water (0.2 mL) was stirred at room temperature overnight. After workup, the residue was precipitated with cool diethyl ether to afford 58 mg (93%) of **37** as a yellow solid. Mp (decomp at 182 °C). ^1^H NMR (400 MHz, DMSO-*d*_6_) δ: 1.65 (m, 6H, CH_2_CH_2_CO), 1.96 (m, 6H, CH_2_CH_2_CO), 3.13 (dd, *J* = 13.9, 7.3 Hz, 3H, β-CH_2_Trp), 3.28 (dd, *J* = 14.0, 6.8 Hz, 3H, β-CH_2_Trp), 3.43 (s, 2H, α-CH_2_Gly), 4.46 (m, 3H, α-CHTrp), 7.06 (t, *J* = 7.5 Hz, 3H, Ar), 7.16 (ddd, *J* = 8.1, 6.9, 1.2 Hz, 3H, Ar), 7.32 (d, *J* = 8.1 Hz, 3H, Ar), 7.45 (dt, *J* = 7.9, 1.4 Hz, 3H, Ar), 7.53 (t, *J* = 8.0 Hz, 3H, Ar), 7.62 (s, 1H, NHCOCH_2_NH_2_), 7.72 (d, *J* = 8.0 Hz, 3H, Ar), 7.80 (t, *J* = 2.0 Hz, 3H, Ar), 7.97 (ddd, *J* = 8.1, 2.4, 1.1 Hz, 3H, Ar), 8.05 (d, *J* = 8.1 Hz, 3H, NH-Trp), 11.58 (br s, 3H, NH-1^*i*^Trp). ^13^C NMR (101 MHz, DMSO-*d*_6_) δ: 27.4 (β-CH_2_Trp), 29.3 (CH_2_CH_2_CO), 30.0 (CH_2_CH_2_CO), 53.4 (α-CHTrp), 57.4 (C(NHCOCH_2_NH_2_)), 111.4, 119.0, 119.3, 119.6, 120.2, 120.5, 120.6, 123.1, 127.3, 130.5, 132.3, 137.4, 140.1, 148.3 (Ar), 165.1 (NHCOCH_2_NH_2_), 171.7 (CH_2_CH_2_CO), 173.1 (COOH). HRMS (ESI^+^) *m*/*z*: calcd for C_63_H_59_N_11_O_16_S_3_ 1321.3303; found 1321.3299.

#### Trimer **38**

Following the general procedure A, a mixture containing trimer **31** (60 mg, 0.04 mmol) in THF (0.7 mL) and LiOH·H_2_O (9 mg, 0.22 mmol) in water (0.1 mL) was stirred at room temperature overnight. After workup, the residue was purified by CCTLC (DCM/methanol/acetic acid, 4:1:0.05) to yield 32 mg (64%) of **38** as a white solid. Mp (decomp at 243 °C). ^1^H NMR (500 MHz, DMSO-*d*_6_) δ: 1.68–1.77 (m, 12H, CH_2_CH_2_CO), 3.00 (br s, 3H, β-CH_2_Trp), 3.29 (br s, 3H, β-CH_2_Trp), 3.41 (s, 2H, α-CH_2_Gly), 4.28 (m, 3H, α-CHTrp), 6.97 (br s, 3H, Ar), 7.08 (t, *J* = 7.5 Hz, 3H, Ar), 7.17 (d, *J* = 8.0 Hz, 6H, Ar), 7.21–7.33 (m, 6H, Ar), 7.43–7.61 (m, 7H, Ar, NHCOCH_2_NH_2_), 7.81 (d, *J* = 8.3 Hz, 2H, NH-Trp), 11.28 (br s, 3H, NH-1^*i*^Trp). ^13^C NMR (126 MHz, DMSO-*d*_6_) δ: 24.6 (β-CH_2_Trp), 29.4 (CH_2_CH_2_CO), 30.4 (CH_2_CH_2_CO), 55.6 (α-CHTrp), 111.5, 119.1, 120.3, 120.8, 121.7, 123.0, 123.6, 125.8, 125.9, 126.2, 126.4, 127.9, 128.3, 137.8, 144.4, 171.4 (Ar, CO). HRMS (ESI^+^) *m*/*z*: calcd for C_66_H_59_F_9_N_8_O_10_S_3_ 1390.3373; found 1390.3369.

#### Trimer **39**

Following the general procedure A, a mixture containing trimer **32** (63 mg, 0.04 mmol) in THF (0.8 mL) and LiOH·H_2_O (10 mg, 0.25 mmol) in water (0.1 mL) was stirred at room temperature overnight. After workup, the residue was purified by CCTLC (DCM/methanol/acetic acid, 4:1:0.05) to yield 25 mg (50%) of **39** as a white solid. Mp (decomp at 205 °C). ^1^H NMR (500 MHz, DMSO-*d*_6_) δ: 1.70 (m, 6H, CH_2_CH_2_CO), 1.97 (m, 6H, CH_2_CH_2_CO), 3.09 (dd, *J* = 14.1, 7.5 Hz, 3H, β-CH_2_Trp), 3.24 (dd, *J* = 14.0, 6.7 Hz, 3H, β-CH_2_Trp), 3.42 (s, 2H, α-CH_2_Gly), 4.43 (m, 3H, α-CHTrp), 7.05 (t, *J* = 7.5 Hz, 3H, Ar), 7.11 (d, *J* = 8.3 Hz, 6H, Ar), 7.15 (t, *J* = 7.8 Hz, 3H, Ar), 7.31 (d, *J* = 8.2 Hz, 3H, Ar), 7.62 (br s, 1H, NHCOCH_2_NH_2_), 7.67 (d, *J* = 8.2 Hz, 6H, Ar), 7.72 (d, *J* = 8.1 Hz, 3H, Ar), 8.02 (d, *J* = 8.2 Hz, 3H, NH-Trp), 11.56 (br s, 3H, NH-1^*i*^Trp). ^13^C NMR (126 MHz, DMSO-*d*_6_) δ: 27.8 (β-CH_2_Trp), 29.8 (CH_2_CH_2_CO), 30.4 (CH_2_CH_2_CO), 40.6 (α-CH_2_Gly), 54.0 (α-CHTrp), 57.9 (C(NHCOCH_2_NH_2_)), 108.1, 111.9, 119.2, 119.7, 119.9, 120.0, 120.1, 123.6, 126.4, 127.8, 133.2, 137.8, 145.4 (Ar), 165.8 (NHCOCH_2_NH_2_), 172.1 (CH_2_CH_2_CO), 173.6 (COOH). HRMS (ESI^+^) *m*/*z*: calcd for C_66_H_59_N_11_O_10_S_3_ 1261.3608; found 1261.3585.

#### Trimer **40**

Following the general procedure A, a mixture containing trimer **33** (70 mg, 0.05 mmol) in THF (1.1 mL) and LiOH·H_2_O (14 mg, 0.33 mmol) in water (0.1 mL) was stirred at room temperature overnight. After workup, the residue was purified by CCTLC (DCM/methanol/acetic acid, 3:1:0.05) to yield 27 mg (40%) of **40** as a white solid. Mp (decomp at 191 °C). ^1^H NMR (500 MHz, DMSO-*d*_6_) δ: 1.67 (m, 6H, CH_2_CH_2_CO), 1.90 (m, 6H, CH_2_CH_2_CO), 2.89 (m, 3H, β-CH_2_Trp), 3.07 (dd, *J* = 13.8, 7.8 Hz, 3H, β-CH_2_Trp), 3.29 (s, 2H, α-CH_2_Gly), 4.37 (m, 3H, α-CHTrp), 6.98 (t, *J* = 7.6 Hz, 3H, Ar), 7.04–7.18 (m, 15H), 7.24 (d, *J* = 8.1 Hz, 3H, Ar), 7.53 (br s, 1H, NHCOCH_2_NH_2_), 7.69 (m, 3H, Ar), 7.73 (d, *J* = 8.1 Hz, 3H, Ar), 11.33 (br s, 3H, NH-1^*i*^Trp). ^13^C NMR (126 MHz, DMSO-*d*_6_) δ: 28.3 (β-CH_2_Trp), 30.2 (CH_2_CH_2_CO), 55.2 (α-CHTrp), 58.0 (C(NHCOCH_2_NH_2_)), 111.5, 116.5, 116.7, 119.3, 119.5, 120.3, 123.0, 123.1, 128.0, 129.6 (d, *J* = 8.0 Hz), 133.0 (d, *J* = 3.0 Hz), 137.6, 161.1 (d, *J* = 242.9 Hz) (Ar), 171.9 (NHCOCH_2_NH_2_), 172.6 (CH_2_CH_2_CO), 174.5 (COOH). HRMS (ESI^+^) *m*/*z*: calcd for C_63_H_59_F_3_N_8_O_10_S_3_ 1240.3468; found 1240.3485.

#### Trimer **41**

Following the general procedure A, a mixture containing trimer **34** (83 mg, 0.05 mmol) in THF (1.0 mL) and LiOH·H_2_O (13 mg, 0.32 mmol) in water (0.2 mL) was stirred at room temperature overnight. After workup, the residue was precipitated with cool diethyl ether to afford 45 mg (65%) of **41** as a white solid. Mp (decomp at 222 °C). ^1^H NMR (400 MHz, DMSO-*d*_6_) δ: 1.70 (m, 6H, CH_2_CH_2_CO), 1.98 (t, *J* = 8.7 Hz, 6H, CH_2_CH_2_CO), 2.48 (s, 9H, COCH_3_), 3.12 (dd, *J* = 14.0, 7.2 Hz, 3H, β-CH_2_Trp), 3.26 (dd, *J* = 14.0, 7.1 Hz, 3H, β-CH_2_Trp), 3.45 (s, 2H, α-CH_2_Gly), 4.47 (m, 3H, α-CHTrp), 7.01–7.12 (m, 9H, Ar), 7.15 (t, *J* = 7.6 Hz, 3H, Ar), 7.31 (d, *J* = 8.2 Hz, 3H, Ar), 7.64–7.73 (m, 4H, Ar, NHCOCH_2_NH_2_), 7.82 (d, *J* = 8.2 Hz, 6H, Ar), 8.15 (d, *J* = 7.9 Hz, 3H, NH-Trp), 11.56 (br s, 3H, NH-1^*i*^Trp), 12.45 (br s, 3H, COOH). ^13^C NMR (101 MHz, DMSO-*d*_6_) δ: 27.0 (COCH_3_), 27.8 (β-CH_2_Trp), 29.7 (CH_2_CH_2_CO), 30.6 (CH_2_CH_2_CO), 36.2 (α-CH_2_Gly), 53.6 (α-CHTrp), 57.7 (C(NHCOCH_2_NH_2_)), 111.9, 119.2, 119.7, 119.9, 121.0, 123.5, 125.8, 127.8, 129.5, 134.4, 137.8, 144.5 (Ar), 165.4 (NHCOCH_2_NH_2_), 172.2 (CH_2_CH_2_CO), 173.5 (COOH), 197.4 (COCH_3_). HRMS (ESI^+^) *m*/*z*: calcd for C_69_H_68_N_8_O_13_S_3_ 1312.4068; found 1312.4065.

#### Trimer **42**

Following the general procedure A, a mixture containing trimer **35** (96 mg, 0.06 mmol) in THF (1.1 mL) and LiOH·H_2_O (14 mg, 0.34 mmol) in water (0.2 mL) was stirred at room temperature overnight. After workup, the residue was precipitated with cool diethyl ether to afford 71 mg (88%) of **42** as a white solid. Mp (decomp at 213 °C). ^1^H NMR (400 MHz, DMSO-*d*_6_) δ: 1.70 (m, 6H, CH_2_CH_2_CO), 1.99 (m, 6H, CH_2_CH_2_CO), 3.10 (m, 3H, β-CH_2_Trp), 3.14 (s, 9H, SO_2_CH_3_), 3.26 (dd, *J* = 14.4, 6.9 Hz, 3H, β-CH_2_Trp), 3.45 (s, 2H, α-CH_2_Gly), 4.47 (m, 3H, α-CHTrp), 7.06 (t, *J* = 7.6 Hz, 3H, Ar), 7.16 (t, *J* = 7.6 Hz, 3H, Ar), 7.21 (d, *J* = 8.2 Hz, 6H, Ar), 7.31 (d, *J* = 8.2 Hz, 3H, Ar), 7.66 (s, 1H, NHCOCH_2_NH_2_), 7.71 (d, *J* = 8.0 Hz, 3H, Ar), 7.78 (d, *J* = 8.2 Hz, 6H, Ar), 8.16 (d, *J* = 8.0 Hz, 3H, NH-Trp), 11.58 (s, 3H, NH-1^*i*^Trp). ^13^C NMR (101 MHz, DMSO-*d*_6_) δ: 27.9 (β-CH_2_Trp), 29.8 (CH_2_CH_2_CO), 30.6 (CH_2_CH_2_CO), 44.1 (SO_2_CH_3_), 53.8 (α-CHTrp), 57.9 (C(NHCOCH_2_NH_2_)), 112.0, 119.6, 119.8, 120.1, 120.6, 123.7, 126.4, 127.9, 128.4, 137.9, 138.1, 145.5 (Ar), 165.6 (NHCOCH_2_NH_2_), 172.3 (CH_2_CH_2_CO), 173.6 (COOH). HRMS (ESI^+^) *m*/*z*: calcd for C_66_H_68_N_8_O_16_S_6_ 1420.3078; found 1420.3078.

#### Methyl 2-Amino-3-(2-(4-nitrophenyl)-1*H*-indol-3-yl)propanoate (**44**)

Under an argon atmosphere, compound **43** (120 mg, 0.47 mmol), 1-iodo-4-nitrobenzene (176 mg, 0.70 mmol), Pd(OAc)_2_ (5 mg, 0.02 mmol), AgBF_4_ (183 mg, 0.94 mmol), and TFA (36 μL, 0.47 mmol) were dissolved in anhydrous DMF (2 mL) in a MW reactor vessel. The mixture was heated to 120 °C for 30 min under MW irradiation (250 W). The resulting suspension was filtered through Whatman filter paper 42, and the volatiles were removed under vacuum. The residue was dissolved in ethyl acetate (20 mL) and washed successively with saturated NaHCO_3_ (3 × 20 mL) and brine (3 × 20 mL). The organic layer was dried over anhydrous Na_2_SO_4_, filtered, and evaporated to dryness. The residue was purified by flash column chromatography (DCM/methanol, 100:3) to afford 73 mg (46%) of **44** as an amorphous orange solid. MS (ES, positive mode): *m*/*z* 340 (M + H)^+^. ^1^H NMR (400 MHz, MeOD) δ: 3.49 (s, 3H, COOCH_3_), 3.57 (dd, *J* = 14.9, 7.3 Hz, 1H. β-CH_2_Trp), 3.70 (dd, *J* = 14.9, 7.3 Hz, 1H, β-CH_2_Trp), 4.19 (m, 1H, α-CHTrp), 7.14 (dt, *J* = 7.1, 1.1 Hz, 1H, Ar), 7.24 (dt, *J* = 7.0, 1.1 Hz, 1H, Ar), 7.46 (d, *J* = 8.2 Hz, 1H, Ar), 7.63 (d, *J* = 8.0 Hz, 1H, Ar), 7.88 (d, *J* = 8.9 Hz, 2H, Ar), 8.37 (d, *J* = 9.0 Hz, 2H, Ar). ^13^C NMR (101 MHz, MeOD) δ: 27.3 (β-CH_2_Trp), 53.5 (COOCH_3_), 54.2 (α-CHTrp), 107.4, 112.8, 112.8, 112.8, 119.5, 121.1, 124.4, 125.1, 125.2, 129.7, 130.0, 130.1, 135.4, 138.4, 140.5, 148.3 (Ar), 170.6 (CO).

#### Trimer **45**

Following the general procedure B, a solution containing the tripodal acid **6**(27) (65 mg, 0.12 mmol), monomer **44** (200 mg, 0.59 mmol), HATU (224 mg, 0.53 mmol), and DIPEA (200 μL, 1.23 mmol) in anhydrous DMF (5 mL) reacted for 24 h. After workup, the residue was purified by CCTLC (DCM/methanol, 30:1) to yield 180 mg (99%) of **45** as an amorphous orange solid. ^1^H NMR (500 MHz, DMSO-*d*_6_) δ: 1.66 (m, 6H, CH_2_CH_2_CO), 1.95 (m, 6H, CH_2_CH_2_CO), 3.27 (m, 3H, β-CH_2_Trp), 3.30 (s, 9H, COOCH_3_), 3.39 (m, 3H, β-CH_2_Trp), 3.57 (d, *J* = 6.1 Hz, 2H, α-CH_2_Gly), 4.19 (m, 1H, COOCH_2_CH), 4.26 (d, *J* = 7.1 Hz, 2H, COOCH_2_CH), 4.55 (m, 3H, α-CHTrp), 7.04 (t, *J* = 7.5 Hz, 3H, Ar), 7.15 (t, *J* = 7.5 Hz, 3H, Ar), 7.29 (tt, *J* = 7.4, 1.4 Hz, 2H, Ar), 7.34–7.41 (m, 6H, Ar, NHCOCH_2_NH), 7.59–7.64 (m, 3H, Ar), 7.68 (dd, *J* = 7.7, 3.3 Hz, 2H, Ar), 7.86 (d, *J* = 7.6 Hz, 2H, Ar), 7.88–7.92 (d, *J* = 8.9 Hz, 6H, Ar), 8.31–8.35 (m, 9H, Ar, NH-Trp), 11.51 (s, 3H, NH-1^*i*^Trp). ^13^C NMR (126 MHz, DMSO-*d*_6_) δ: 27.2 (β-CH_2_Trp), 29.1 (CH_2_CH_2_CO), 29.9 (CH_2_CH_2_CO), 43.5 (α-CH_2_Gly), 46.6 (COOCH_2_CH), 51.7 (COOCH_3_), 53.0 (α-CHTrp), 56.8 (C(NHCOCH_2_NH)), 65.7 (COOCH_2_CH), 109.9, 111.5, 119.2, 119.4, 120.1, 121.3, 122.8, 124.0, 125.2, 127.0, 127.6, 128.5, 128.6, 129.5, 132.8, 136.5, 139.2, 140.7, 143.9, 146.0, 151.9 (Ar), 156.4 (COOCH_2_CH), 168.1 (NHCOCH_2_NH), 172.0 (COOCH_3_), 172.2 (CH_2_CH_2_CO).

#### Trimer **46**

Following the general procedure A, a mixture containing trimer **45** (100 mg, 0.08 mmol) in THF (3.0 mL) and LiOH·H_2_O (20 mg, 0.47 mmol) in water (1.0 mL) was stirred at room temperature overnight. After workup, the residue was precipitated with cool diethyl ether to afford 95 mg (96%) of **46** as an amorphous orange solid. ^1^H NMR (400 MHz, DMSO-*d*_6_) δ: 1.72 (m, 6H, CH_2_CH_2_CO), 1.98 (m, 6H, CH_2_CH_2_CO), 3.20 (dd, *J* = 14.1, 7.3 Hz, 3H, β-CH_2_Trp), 3.36–3.47 (m, 5H, β-CH_2_Trp, α-CH_2_Gly), 4.57 (m, 3H, α-CHTrp), 6.99 (t, *J* = 7.5 Hz, 3H, Ar), 7.11 (t, *J* = 7.5 Hz, 3H, Ar), 7.36 (d, *J* = 8.1 Hz, 3H, Ar), 7.73 (d, *J* = 8.5 Hz, 3H, NH-Trp), 7.79 (d, *J* = 8.0 Hz, 3H, Ar), 8.02 (d, *J* = 8.4 Hz, 6H, Ar), 8.26 (d, *J* = 8.5 Hz, 6H, Ar), 11.24 (s, 3H, NH-1^*i*^Trp). ^13^C NMR (101 MHz, DMSO-*d*_6_) δ: 27.9 (β-CH_2_Trp), 29.7 (CH_2_CH_2_CO), 29.9 (CH_2_CH_2_CO), 42.0 (α-CH_2_Gly), 54.2 (α-CHTrp), 57.4 (C(NHCOCH_2_NH_2_)), 111.0, 112.0, 118.8, 119.6, 122.3, 123.4, 128.2, 129.0, 132.2, 136.4, 139.3, 145.6 (Ar), 171.5 (CO). HRMS (ESI^–^) *m*/*z*: calcd for C_63_H_59_N_11_O_16_ 1225.4139; found 1225.4141.

#### (9*H*-Fluoren-9-yl)methyl (1-Methoxy-3-(2-(4-nitrobenzoyl)-1*H*-indol-3-yl)-1-oxopropan-2-yl)-λ^2^-azanecarboxylate (**47**)

To a cold solution of **10**(29) (400 mg, 0.91 mmol) and 4-nitrobenzoyl chloride (253 mg, 1.36 mmol) in anhydrous DCM (1.8 mL), SnCl_4_ (319 μL, 2.72 mmol) was added dropwise. The resulting mixture was stirred at 0 °C for 3 h. Then, it was quenched with 1 N HCl (10 mL) and diluted with DCM (20 mL). The organic layer was washed with brine, dried over Na_2_SO_4_, filtered, and evaporated to dryness. The residue was subjected to column chromatography (hexane/ethyl acetate, 2:1) to yield 198 mg (37%) of **47** as an amorphous white solid. MS (ES, positive mode): *m*/*z* 590 (M + H)^+^. ^1^H NMR (400 MHz, DMSO-*d*_6_) δ: 3.28 (m, 1H, β-CH_2_Trp), 3.41–3.51 (m, 4H, β-CH_2_Trp, COOCH_3_), 4.05–4.21 (m, 3H, COOCH_2_CH), 4.36 (m, 1H, α-CHTrp), 7.12 (t, *J* = 7.5 Hz, 1H, Ar), 7.21–7.34 (m, 3H, Ar), 7.36–7.46 (m, 3H, Ar), 7.57 (dd, *J* = 11.7, 7.5 Hz, 2H, Ar), 7.79 (d, *J* = 8.2 Hz, 1H, Ar), 7.83–7.90 (m, 3H, Ar, NH-Trp), 7.98 (d, *J* = 8.7 Hz, 2H, Ar), 8.40 (d, *J* = 8.7 Hz, 2H, Ar), 11.59 (br s, 1H, NH-1^*i*^Trp).

#### Methyl 1-(4-Nitrophenyl)-9*H*-pyrido[3,4-*b*]indole-3-carboxylate (**48**)

To a clear solution of the monomer **47** (190 mg, 0.32 mmol) in DCM (3.2 mL), piperidine (318 μL, 3.22 mmol) was added dropwise and a yellow solid precipitated. After 2 h, the solid was filtrated and washed with DCM to afford 59 mg (53%) of **48** as an amorphous yellow solid. MS (ES, positive mode): *m*/*z* 348 (M + H)^+^. ^1^H NMR (400 MHz, DMSO-*d*_6_) δ: 3.95 (s, 3H, COOCH_3_), 7.36 (ddd, *J* = 8.0, 7.0, 1.1 Hz, 1H, Ar), 7.64 (ddd, *J* = 8.2, 7.0, 1.2 Hz, 1H, Ar), 7.71 (dt, *J* = 8.3, 1.0 Hz, 1H, Ar), 8.32 (m, 2H, Ar), 8.45–8.51 (m, 3H, Ar), 9.01 (s, 1H, Ar). ^1^H NMR data are in agreement with those previously described for this compound.^37^

#### Trimer **49**

To a cold solution of trimer **7** (50 mg, 0.04 mmol) and 4-nitrobenzoyl chloride (37 mg, 0.20 mmol) in anhydrous DCM (0.5 mL), SnCl_4_ (46 μL, 0.40 mmol) was added dropwise. The resulting mixture was allowed to reach rt and for 8.5 h. Then, it was quenched with 1 N HCl (5 mL) and diluted with ethyl acetate (10 mL). The organic layer was washed with brine, dried over Na_2_SO_4_, filtered, and evaporated to dryness. The residue was purified by CCTLC (DCM/methanol, 40:1) to yield 24 mg (21%) of **49** as an amorphous yellow solid. ^1^H NMR (400 MHz, CDCl_3_) δ: 1.63–1.85 (m, 12H, CH_2_CH_2_CO), 3.27 (dd, *J* = 13.9, 10.4 Hz, 3H, β-CH_2_Trp), 3.39 (dd, *J* = 13.7, 4.8 Hz, 3H, β-CH_2_Trp), 3.60 (s, 9H, COOCH_3_), 4.05 (m, 2H, COOCH_2_CH), 4.19 (m, 1H, COOCH_2_CH), 4.52 (m, 3H, α-CHTrp), 7.04 (t, *J* = 7.4 Hz, 3H, Ar), 7.15–7.22 (m, 4H, Ar), 7.30 (t, *J* = 7.5 Hz, 3H, Ar), 7.44–7.55 (m, 5H, Ar), 7.60–7.74 (m, 5H, Ar, NH-Trp), 7.86 (d, *J* = 8.3 Hz, 6H, Ar), 8.05–8.24 (m, 9H, Ar), 9.88 (br s, 3H, NH-1^*i*^Trp).

#### Trimer **50**

Following the general procedure A, a mixture containing trimer **49** (24 mg, 0.02 mmol) in THF (0.2 mL) and LiOH·H_2_O (4 mg, 0.09 mmol) in water (0.1 mL) was stirred at room temperature overnight. After workup, the residue was precipitated with cool diethyl ether to afford 10 mg (51%) of **50** as a yellow solid. Mp (decomp at 202 °C). ^1^H NMR (500 MHz, DMSO-*d*_6_) δ: 1.45 (m, 3H, CH_2_CH_2_CO), 1.62 (m, 3H, CH_2_CH_2_CO), 1.86 (m, 6H, CH_2_CH_2_CO), 3.23 (dd, *J* = 13.6, 7.9 Hz, 3H, β-CH_2_Trp), 3.39–3.44 (m, 5H, β-CH_2_Trp, α-CH_2_Gly), 4.46 (m, 3H, α-CHTrp), 7.09 (ddd, *J* = 8.0, 6.8, 1.0 Hz, 3H, Ar), 7.27 (ddd, *J* = 8.2, 6.8, 1.1 Hz, 3H, Ar), 7.40 (d, *J* = 8.2 Hz, 3H, Ar), 7.56 (br s, 1H, NHCOCH_2_NH_2_), 7.79 (d, *J* = 8.2 Hz, 3H, Ar), 7.96 (m, 6H, Ar), 8.07 (d, *J* = 7.8 Hz, 3H, NH-Trp), 8.39 (m, 6H, Ar), 11.52 (s, 3H, NH-1^*i*^Trp). ^13^C NMR (126 MHz, DMSO-*d*_6_) δ: 27.6 (β-CH_2_Trp), 29.6 (CH_2_CH_2_CO), 30.4 (CH_2_CH_2_CO), 53.6 (α-CHTrp), 57.6 (C(NHCOCH_2_NH_2_)), 113.3, 120.7, 121.0, 121.5, 124.2, 126.3, 128.1, 130.7, 131.6, 137.4, 144.7, 149.7 (Ar), 165.2 (NHCOCH_2_NH_2_), 172.0 (CH_2_CH_2_CO), 173.4 (COOH), 187.5 (COAr). HRMS (ESI^+^) *m*/*z*: calcd for C_66_H_59_N_11_O_19_ 1309.3989; found 1309.3970.

#### Trimer **51**

Following the general procedure E, trimer **8** (282 mg, 0.18 mmol) and piperidine (176 μL, 1.77 mmol) in DCM (4.0 mL) reacted for 2.5 h. After workup, the residue was subjected to column chromatography (DCM/methanol, 10:1) to yield 177 mg (73%) of **51** as a yellow oil. ^1^H NMR (400 MHz, DMSO-*d*_6_) δ: 1.70 (m, 6H, CH_2_CH_2_CO), 1.97 (t, *J* = 8.5 Hz, 6H, CH_2_CH_2_CO), 3.10–3.18 (m, 5H, β-CH_2_Trp, α-CH_2_Gly), 3.23 (dd, *J* = 14.0, 7.8 Hz, 3H, β-CH_2_Trp), 3.42 (s, 9H, COOCH_3_), 4.47 (m, 3H, α-CHTrp), 7.08 (t, *J* = 7.5 Hz, 3H, Ar), 7.14–7.23 (m, 9H, Ar), 7.29 (br s, 1H, NHCOCH_2_NH_2_), 7.35 (d, *J* = 8.2 Hz, 3H, Ar), 7.62 (d, *J* = 8.0 Hz, 3H, Ar), 8.11 (d, *J* = 9.0 Hz, 6H, Ar), 8.33 (d, *J* = 7.6 Hz, 3H, NH-Trp), 11.68 (br s, 3H, NH-1^*i*^Trp). HRMS (ESI^+^) *m*/*z*: calcd for C_66_H_65_N_11_O_16_S_3_ 1363.3773; found 1363.3756.

#### Trimer **52**

To a cooled solution of **51** (79 mg, 0.06 mmol) in anhydrous DCM (0.6 mL), propylene oxide (81 μL, 1.16 mmol) and hexanoyl chloride (12 μL, 0.09 mmol) were added dropwise. The reaction was stirred at rt for 2 h, and then volatiles were removed. The residue was purified by CCTLC (DCM/methanol, 20:0.8) to yield 35 mg (41%) of **52** as a yellow oil. ^1^H NMR (400 MHz, CDCl_3_) δ: 0.89 (t, *J* = 3.5 Hz, 3H, (CH_2_)_4_CH_3_), 1.23–1.34 (m, 8H, (CH_2_)_4_CH_3_), 1.59 (m, 6H, CH_2_CH_2_CO), 1.92 (m, 6H, CH_2_CH_2_CO), 3.20 (dd, *J* = 14.3, 8.1 Hz, 3H, β-CH_2_Trp), 3.39 (dd, *J* = 14.2, 5.5 Hz, 3H, β-CH_2_Trp), 3.63 (m, 2H, α-CH_2_Gly), 3.67 (s, 9H, COOCH_3_), 4.85 (m, 3H, α-CHTrp), 6.39 (br s, 1H, NHCOCH_2_NH), 6.72 (br s, 3H, NH-Trp), 7.04 (d, *J* = 8.6 Hz, 6H, Ar), 7.13 (t, *J* = 7.5 Hz, 3H, Ar), 7.22 (t, *J* = 7.5 Hz, 3H, Ar), 7.31 (m, 3H, Ar), 7.60 (d, *J* = 8.0 Hz, 3H, Ar), 7.98 (d, *J* = 8.6 Hz, 6H, Ar), 9.12 (br s, 3H, NH-1^*i*^Trp).

#### Trimer **53**

To a cooled solution of **51** (60 mg, 0.04 mmol) in anhydrous DCM (0.5 mL), propylene oxide (62 μL, 0.88 mmol) and octanoyl chloride (11 μL, 0.07 mmol) were added dropwise. The reaction was stirred at rt for 3.5 h, and then volatiles were removed. The residue was purified by CCTLC (DCM/methanol, 33:1) to yield 29 mg (44%) of **53** as a yellow oil. ^1^H NMR (400 MHz, CDCl_3_) δ: 0.82–0.88 (m, 5H, (CH_2_)_6_CH_3_), 1.16–1.32 (m, 10H, (CH_2_)_6_CH_3_), 1.50 (m, 3H, CH_2_CH_2_CO), 1.69 (m, 3H, CH_2_CH_2_CO), 1.91 (m, 6H, CH_2_CH_2_CO), 3.20 (dd, *J* = 14.8, 7.4 Hz, 3H, β-CH_2_Trp), 3.37 (dd, *J* = 14.6, 5.4 Hz, 3H, β-CH_2_Trp), 3.57 (m, 2H, α-CH_2_Gly), 3.65 (s, 9H, COOCH_3_), 4.86 (m, 3H, α-CHTrp), 6.34 (br s, 1H, NHCOCH_2_NH), 6.76 (br s, 3H, NH-Trp), 7.00 (d, *J* = 8.5 Hz, 6H, Ar), 7.11 (t, *J* = 7.5 Hz, 3H, Ar), 7.20 (t, *J* = 7.6 Hz, 3H, Ar), 7.28 (m, 3H, Ar), 7.58 (d, *J* = 8.0 Hz, 3H, Ar), 7.95 (d, *J* = 8.4 Hz, 6H, Ar), 9.38 (br s, 3H, NH-1^*i*^Trp).

#### Trimer **54**

To a cooled solution of **51** (77 mg, 0.06 mmol) in anhydrous DCM (0.5 mL), propylene oxide (78 μL, 1.12 mmol) and decanoyl chloride (18 μL, 0.09 mmol) were added dropwise. The reaction was stirred at rt for 4 h, and then volatiles were removed. The residue was purified by CCTLC (DCM/methanol, 30:1) to yield 25 mg (30%) of **54** as a yellow oil. ^1^H NMR (400 MHz, CDCl_3_) δ: 0.83–0.90 (m, 6H, (CH_2_)_8_CH_3_), 1.19–1.27 (m, 13H, (CH_2_)_8_CH_3_), 1.51 (m, 3H, CH_2_CH_2_CO), 1.70 (m, 3H, CH_2_CH_2_CO), 1.92 (m, 6H, CH_2_CH_2_CO), 3.20 (dd, *J* = 14.3, 7.8 Hz, 3H, β-CH_2_Trp), 3.38 (dd, *J* = 14.3, 5.6 Hz, 3H, β-CH_2_Trp), 3.62 (m, 2H, α-CH_2_Gly), 3.66 (s, 9H, COOCH_3_), 4.85 (td, *J* = 7.9, 5.6 Hz, 3H, α-CHTrp), 6.25 (br s, 1H, NHCOCH_2_NH), 6.63 (br s, 3H, NH-Trp), 7.03 (d, *J* = 8.9 Hz, 6H, Ar), 7.12 (t, *J* = 7.5 Hz, 3H, Ar), 7.22 (t, *J* = 7.5 Hz, 3H, Ar), 7.28 (m, 3H, Ar), 7.59 (d, *J* = 8.0 Hz, 3H, Ar), 7.96 (d, *J* = 8.9 Hz, 6H, Ar), 9.22 (br s, 3H, NH-1^*i*^Trp).

#### Trimer **55**

To a cooled solution of **51** (50 mg, 0.04 mmol) in anhydrous DCM (0.5 mL), propylene oxide (52 μL, 0.74 mmol) and lauroyl chloride (13 μL, 0.05 mmol) were added dropwise. The reaction was stirred at rt for 5 h, and then volatiles were removed. The residue was purified by CCTLC (DCM/methanol, 28:1) to yield 34 mg (59%) of **55** as a yellow oil. ^1^H NMR (400 MHz, CDCl_3_) δ: 0.83–0.89 (m, 7H, (CH_2_)_10_CH_3_), 1.15–1.30 (m, 16H, (CH_2_)_10_CH_3_), 1.51 (m, 3H, CH_2_CH_2_CO), 1.71 (m, 3H, CH_2_CH_2_CO), 1.92 (m, 6H, CH_2_CH_2_CO), 3.20 (dd, *J* = 14.3, 7.6 Hz, 3H, β-CH_2_Trp), 3.38 (dd, *J* = 14.3, 5.4 Hz, 3H, β-CH_2_Trp), 3.59 (s, 2H, α-CH_2_Gly), 3.66 (s, 9H, COOCH_3_), 4.86 (m, 3H, α-CHTrp), 6.30 (br s, 1H, NHCOCH_2_NH), 6.71 (br s, 3H, NH-Trp), 7.01 (d, *J* = 8.6 Hz, 6H, Ar), 7.12 (t, *J* = 7.4 Hz, 3H, Ar, 7.21 (t, *J* = 7.5 Hz, 3H, Ar), 7.29 (m, 3H, Ar), 7.59 (d, *J* = 8.0 Hz, 3H, Ar), 7.96 (d, *J* = 8.5 Hz, 6H, Ar), 9.33 (br s, 3H, NH-1^*i*^Trp).

#### Trimer **56**

Following the general procedure A, a mixture containing trimer **52** (35 mg, 0.02 mmol) in THF (0.5 mL) and LiOH·H_2_O (6 mg, 0.14 mmol) in water (0.1 mL) was stirred at room temperature overnight. After workup, the residue was precipitated with cool diethyl ether to afford 14 mg (41%) of **56** as an amorphous yellow solid. ^1^H NMR (500 MHz, DMSO-*d*_6_) δ: 0.80 (m, 3H, (CH_2_)_4_CH_3_), 1.16–1.27 (m, 8H, (CH_2_)_4_CH_3_)), 1.67 (m, 6H, CH_2_CH_2_CO), 1.96 (m, 6H, CH_2_CH_2_CO), 3.11 (dd, *J* = 14.1, 7.2 Hz, 3H, β-CH_2_Trp), 3.24 (dd, *J* = 14.0, 6.9 Hz, 3H, β-CH_2_Trp), 3.62 (d, *J* = 5.3 Hz, 2H, α-CH_2_Gly), 4.47 (m, 3H, α-CHTrp), 7.06 (ddd, *J* = 8.1, 5.5, 1.0 Hz, 3H, Ar), 7.12–7.21 (m, 9H, Ar), 7.32 (dt, *J* = 8.1, 0.9 Hz, 3H, Ar), 7.71 (d, *J* = 8.1 Hz, 3H, Ar), 7.85 (t, *J* = 5.6 Hz, 1H, NHCOCH_2_NH), 8.09 (d, *J* = 9.1 Hz, 6H), 8.12 (m, 3H, NH-Trp), 11.62 (br s, 3H, NH-1^*i*^Trp), 12.48 (br s, 3H, COOH). ^13^C NMR (126 MHz, DMSO-*d*_6_) δ: 14.3 ((CH_2_)_4_CH_3_), 22.3, 25.3 ((CH_2_)_4_CH_3_), 27.7 (β-CH_2_Trp), 29.5, 29.8, 30.5, 31.4, 35.6, 53.5, 57.1 (CH_2_CH_2_CO, CH_2_CH_2_CO, (CH_2_)_4_CH_3_), 112.0, 119.6, 119.81, 120.1, 123.7, 124.7, 126.2, 127.8, 137.9, 145.4, 148.0 (Ar), 168.5, 172.4, 173.0, 173.4 (NHCOCH_2_NH, CH_2_CH_2_CO, NHCOCH_2_NHCO, COOH). HRMS (ESI^+^) *m*/*z*: calcd for C_69_H_69_N_11_O_17_S_3_ 1419.4035; found 1419.3996.

#### Trimer **57**

Following the general procedure A, a mixture containing trimer **53** (29 mg, 0.02 mmol) in THF (0.4 mL) and LiOH·H_2_O (5 mg, 0.12 mmol) in water (0.1 mL) was stirred at room temperature overnight. After workup, the residue was precipitated with cool diethyl ether to afford 28 mg (quantitative yield) of **57** as a yellow solid. Mp (decomp at 154 °C). ^1^H NMR (400 MHz, DMSO-*d*_6_) δ: 0.81 (t, *J* = 6.8 Hz, 3H, (CH_2_)_6_CH_3_), 1.15–1.27 (m, 8H, (CH_2_)_6_CH_3_), 1.41–1.49 (m, 2H, (CH_2_)_6_CH_3_), 1.67 (m, 6H, CH_2_CH_2_CO), 1.97 (t, *J* = 8.6 Hz, 6H, CH_2_CH_2_CO), 2.09 (t, *J* = 7.6 Hz, 2H, (CH_2_)_6_CH_3_), 3.12 (dd, *J* = 14.0, 7.2 Hz, 3H, β-CH_2_Trp), 3.25 (dd, *J* = 14.1, 6.9 Hz, 3H, β-CH_2_Trp), 3.63 (d, *J* = 5.4 Hz, 2H, α-CH_2_Gly), 4.47 (m, 3H, α-CHTrp), 7.07 (t, *J* = 7.5 Hz, 3H, Ar), 7.13–7.22 (m, 10H, Ar, NHCOCH_2_NH), 7.32 (d, *J* = 8.2 Hz, 3H, Ar), 7.71 (d, *J* = 8.0 Hz, 3H, Ar), 7.85 (m, 1H, NHCOCH_2_NH), 8.07–8.15 (m, 9H, Ar, NH-Trp), 11.62 (br s, 3H, NH-1^*i*^Trp), 12.49 (br s, 3H, COOH). ^13^C NMR (101 MHz, DMSO-*d*_6_) δ: 14.4 ((CH_2_)_6_CH_3_), 22.5, 25.7 ((CH_2_)_6_CH_3_), 27.7 (β-CH_2_Trp), 28.9, 29.2, 29.8, 30.6, 31.6, 35.6, 36.2 (CH_2_CH_2_CO, CH_2_CH_2_CO, (CH_2_)_6_CH_3_), 53.5 (α-CHTrp), 57.2 (C(NHCOCH_2_NH_2_)), 112.0, 119.6, 119.8, 120.1, 123.7, 124.7, 126.2, 127.8, 137.9, 145.4, 148.0 (Ar), 162.8 (NHCOCH_2_NH), 172.4, 173.1 (CH_2_CH_2_CO, NHCOCH_2_NHCO), 173.4 (COOH). HRMS (ESI^+^) *m*/*z*: calcd for C_71_H_73_N_11_O_17_S_3_ 1447.4348; found 1447.4320.

#### Trimer **58**

Following the general procedure A, a mixture containing trimer **54** (25 mg, 0.02 mmol) in THF (0.3 mL) and LiOH·H_2_O (4 mg, 0.10 mmol) in water (0.1 mL) was stirred at room temperature overnight. After workup, the residue was precipitated with cool diethyl ether to afford 24 mg (quantitative yield) of **58** as an amorphous yellow solid. ^1^H NMR (500 MHz, DMSO-*d*_6_) δ: 0.82 (t, *J* = 6.9 Hz, 3H, (CH_2_)_8_CH_3_), 1.16–1.20 (m, 10H, (CH_2_)_8_CH_3_), 1.22–1.26 (m, 6H, (CH_2_)_8_CH_3_), 1.67 (m, 6H, CH_2_CH_2_CO), 1.96 (m, 6H, CH_2_CH_2_CO), 3.11 (dd, *J* = 14.0, 7.2 Hz, 3H, β-CH_2_Trp), 3.24 (dd, *J* = 14.1, 6.9 Hz, 3H, β-CH_2_Trp), 3.62 (d, *J* = 5.3 Hz, 2H, α-CH_2_Gly), 4.47 (m, 3H, α-CHTrp), 7.06 (t, *J* = 8.0 Hz, 3H. Ar), 7.14–7.21 (m, 9H, Ar), 7.32 (d, *J* = 8.1 Hz, 3H, Ar), 7.70 (d, *J* = 8.0 Hz, 3H, Ar), 7.86 (t, *J* = 5.6 Hz, 1H, NHCOCH_2_NH), 8.09 (d, *J* = 9.0 Hz, 6H, Ar), 8.13 (d, *J* = 8.0 Hz, 3H, NH-Trp), 11.62 (br s, 3H, NH-1^*i*^Trp), 12.43 (br s, 3H, COOH). ^13^C NMR (126 MHz, DMSO-*d*_6_) δ: 14.4 ((CH_2_)_8_CH_3_), 22.5, 25.7 ((CH_2_)_8_CH_3_), 27.7 (β-CH_2_Trp), 29.1, 29.2, 29.3, 29.4, 29.8, 30.5, 31.7, 34.1, 35.6, 53.4, 57.1 (CH_2_CH_2_CO, CH_2_CH_2_CO (CH_2_)_8_CH_3_), 112.0, 119.6, 119.8, 120.1, 123.7, 124.7, 126.2, 127.8, 137.9, 145.4, 148.0 (Ar), 168.5 (NHCOCH_2_NH), 172.4, 173.1, 173.4 (CH_2_CH_2_CO, NHCOCH_2_NHCO, COOH). HRMS (ESI^+^) *m*/*z*: calcd for C_73_H_77_N_11_O_17_S_3_ 1475.4661; found 1475.4647.

#### Trimer **59**

Following the general procedure A, a mixture containing trimer **55** (34 mg, 0.02 mmol) in THF (0.5 mL) and LiOH·H_2_O (6 mg, 0.13 mmol) in water (0.1 mL) was stirred at room temperature overnight. After workup, the residue was precipitated with cool diethyl ether to afford 27 mg (82%) of **59** as a yellow solid. Mp (decomp at 118 °C). ^1^H NMR (400 MHz, DMSO-*d*_6_) δ: 0.82 (t, *J* = 7.1 Hz, 3H, (CH_2_)_10_CH_3_), 1.16–1.26 (m, 16H, (CH_2_)_10_CH_3_), 1.67 (m, 6H, CH_2_CH_2_CO), 1.97 (m, 6H, CH_2_CH_2_CO), 2.09 (t, *J* = 7.6 Hz, 2H, (CH_2_)_10_CH_3_), 2.18 (t, *J* = 7.4 Hz, 2H, (CH_2_)_10_CH_3_), 3.12 (dd, *J* = 14.0, 7.2 Hz, 3H, β-CH_2_Trp), 3.25 (dd, *J* = 14.1, 6.9 Hz, 3H, β-CH_2_Trp), 3.62 (d, *J* = 5.4 Hz, 2H, α-CH_2_Gly), 4.47 (m, 3H, α-CHTrp), 7.06 (t, *J* = 7.5 Hz, 3H, Ar), 7.13–7.23 (m, 10H, Ar, NHCOCH_2_NH), 7.32 (d, *J* = 8.1 Hz, 3H, Ar), 7.71 (d, *J* = 8.0 Hz, 3H, Ar), 7.85 (t, *J* = 5.4 Hz, 1H, NHCOCH_2_NH), 8.10 (d, *J* = 8.2 Hz, 6H, Ar), 8.12 (m, 3H, NH-Trp), 11.62 (br s, 3H, NH-1^*i*^Trp), 12.45 (br s, 3H, COOH). ^13^C NMR (101 MHz, DMSO-*d*_6_) δ: 14.4 ((CH_2_)_10_CH_3_), 22.6, 25.0, 25.7 ((CH_2_)_10_CH_3_), 27.7 (β-CH_2_Trp), 29.0, 29.1, 29.2, 29.3, 29.4, 29.5, 29.6 (CH_2_CH_2_CO, CH_2_CH_2_CO, (CH_2_)_10_CH_3_), 31.8, 34.1, 35.6 ((CH_2_)_10_CH_3_), 40.7 (α-CH_2_Gly), 53.4 (α-CHTrp), 57.2 (C(NHCOCH_2_NH_2_)), 112.0, 119.6, 119.8, 120.1, 120.2, 123.7, 124.7, 126.2, 127.8, 137.9, 145.4, 148.0 (Ar), 168.5 (NHCOCH_2_NH), 172.4, 173.4 (CH_2_CH_2_CO, NHCOCH_2_NHCO), 175.0 (COOH). HRMS (ESI^+^) *m*/*z*: calcd for C_75_H_81_N_11_O_17_S_3_ 1503.4974; found 1503.4958.

#### Trimer **60**

To a solution containing trimer **51** (41 mg, 0.03 mmol), monomethyl adipate (6 μL, 0.04 mmol), and HATU (17 mg, 0.04 mmol) in anhydrous DMF (0.5 mL), DIPEA (10 μL, 0.06 mmol) was added. The resulting mixture was heated to 30 °C for 24 h. Then, it was quenched with a saturated solution of NH_4_Cl (5 mL) and volatiles were removed. The residue was dissolved in ethyl acetate (20 mL) and washed with water (10 mL). The organic layer was dried over Na_2_SO_4_, filtered, and evaporated to dryness, and the residue was purified by CCTLC (DCM/methanol, 10:1) to yield 24 mg (53%) of **60** as an amorphous yellow solid. ^1^H NMR (400 MHz, MeOD) δ: 1.47–1.53 (m, 4H, NHCOCH_2_CH_2_CH_2_CH_2_COOH), 1.62 (m, 6H, CH_2_CH_2_CO), 1.88 (m, 6H, CH_2_CH_2_CO), 2.16–2.21 (m, 4H, NHCOCH_2_CH_2_CH_2_CH_2_COOH), 3.15 (dd, *J* = 14.2, 7.6 Hz, 3H, β-CH_2_Trp), 3.29 (dd, *J* = 14.2, 6.6 Hz, 3H, β-CH_2_Trp), 3.47 (s, 9H, NHCHCOOCH_3_), 3.50 (s, 3H, (CH_2_)_4_COOCH_3_), 3.57 (s, 2H, α-CH_2_Gly), 4.60 (t, *J* = 7.0 Hz, 3H, α-CHTrp), 6.97 (t, *J* = 7.5 Hz, 3H, Ar), 7.03–7.11 (m, 9H, Ar), 7.24 (d, *J* = 8.2 Hz, 3H, Ar), 7.52 (d, *J* = 8.0 Hz, 3H, Ar), 7.95 (d, *J* = 8.6 Hz, 6H, Ar).

#### Trimer **61**

Following the general procedure A, a mixture containing trimer **60** (24 mg, 0.02 mmol) in THF (0.3 mL) and LiOH·H_2_O (5 mg, 0.13 mmol) in water (0.1 mL) was stirred at room temperature overnight. After workup, the residue was precipitated with cool diethyl ether to afford 14 mg (60%) of **61** as an amorphous yellow solid. ^1^H NMR (500 MHz, DMSO-*d*_6_) δ: 1.40–1.52 (m, 4H, NHCOCH_2_CH_2_CH_2_CH_2_COOH), 1.68 (m, 6H, CH_2_CH_2_CO), 1.90 (m, 6H, CH_2_CH_2_CO), 2.11 (t, *J* = 7.0 Hz, 2H, NHCOCH_2_CH_2_CH_2_CH_2_COOH), 2.15 (t, *J* = 6.9 Hz, 2H, NHCOCH_2_CH_2_CH_2_CH_2_COOH), 3.10 (dd, *J* = 14.0, 7.4 Hz, 3H, β-CH_2_Trp), 3.24 (dd, *J* = 14.0, 6.8 Hz, 3H, β-CH_2_Trp), 3.62 (d, *J* = 5.5 Hz, 2H, α-CH_2_Gly), 4.45 (m, 3H, α-CHTrp), 7.06 (t, *J* = 7.3 Hz, 3H, Ar), 7.12–7.22 (m, 9H, Ar), 7.31 (d, *J* = 8.2 Hz, 3H, Ar), 7.71 (d, *J* = 7.9 Hz, 3H, Ar), 7.87 (br s, 1H, NHCOCH_2_NH), 8.09–8.14 (m, 9H, Ar), 11.60 (br s, 3H, NH-1^*i*^Trp). ^13^C NMR (126 MHz, DMSO-*d*_6_) δ: 24.6, 25.2 (NHCOCH_2_CH_2_CH_2_CH_2_COOH), 27.7 (β-CH_2_Trp), 29.8 (CH_2_CH_2_CO), 30.5 (CH_2_CH_2_CO), 33.8, 35.3 (NHCOCH_2_CH_2_CH_2_CH_2_COOH), 38.5 (α-CH_2_Gly), 53.4 (α-CHTrp), 57.1 (C(NHCOCH_2_NHCO)), 112.0, 119.6, 119.8, 120.0, 120.1, 123.7, 124.7, 126.2, 127.8, 137.9, 145.4, 148.0 (Ar), 168.5 (NHCOCH_2_NHCO), 172.4 (CH_2_CH_2_CO), 172.7 (NHCOCH_2_NHCO), 173.4, 174.8 (COOH). HRMS (ESI^+^) *m*/*z*: calcd for C_69_H_67_N_11_O_19_S_3_ 1449.3777; found 1449.3788.

#### Trimer **62**

To a cold solution containing trimer **51** (50 mg, 0.04 mmol), Fmoc-9-amino-4,7-dioxanonanoic acid (18 mg, 0.04 mmol), and HATU (21 mg, 0.06 mmol) in anhydrous DMF (0.4 mL), DIPEA (13 μL, 0.07 mmol) was added dropwise. The resulting mixture was heated to 30 °C for 24 h. Then, it was quenched with a saturated solution of NH_4_Cl (5 mL) and volatiles were removed. The residue was dissolved in ethyl acetate (20 mL) and washed with water (10 mL). The organic layer was dried over Na_2_SO_4_, filtered, and evaporated to dryness, and the residue was purified by CCTLC (DCM/methanol, 14:1) to yield 58 mg (90%) of **62** as an amorphous yellow solid. ^1^H NMR (500 MHz, CDCl_3_) δ: 1.43 (m, 3H, CH_2_CH_2_CO), 1.59 (s, 3H, CH_2_CH_2_CO), 1.87 (s, 6H, CH_2_CH_2_CO), 2.32 (m, 2H, CH_2_), 2.42–2.72 (m, 4H, CH_2_), 3.13 (dd, *J* = 14.4, 7.7 Hz, 3H, β-CH_2_Trp), 3.21 (m, 2H, CH_2_), 3.31 (dd, *J* = 14.4, 5.5 Hz, 3H, β-CH_2_Trp), 3.40 (m, 2H, CH_2_), 3.47 (s, 2H, α-CH_2_Gly), 3.54 (m, 4H, CH_2_), 3.58 (s, 9H, COOCH_3_), 4.06 (t, *J* = 6.8 Hz, 1H, COOCH_2_CH), 4.24 (d, *J* = 6.8 Hz, 2H, COOCH_2_CH), 4.78 (m, 3H, α-CHTrp), 6.73 (br s, 3H, NH-Trp), 6.86 (br s, 1H, NH), 6.92 (d, *J* = 8.6 Hz, 6H, Ar), 7.04 (t, *J* = 7.5 Hz, 3H, Ar), 7.12 (t, *J* = 7.6 Hz, 3H, Ar), 7.15–7.22 (m, 5H, Ar), 7.29 (dd, *J* = 8.2, 6.7 Hz, 2H, Ar), 7.46 (m, 2H, Ar), 7.51 (d, *J* = 8.0 Hz, 3H, Ar), 7.66 (d, *J* = 7.6 Hz, 2H, Ar), 7.87 (d, *J* = 8.6 Hz, 6H, Ar), 9.22 (s, 3H, NH-1^*i*^Trp).

#### Trimer **63**

Following the general procedure A, a mixture containing trimer **62** (18 mg, 0.01 mmol) in THF (0.4 mL) and LiOH·H_2_O (3 mg, 0.07 mmol) in water (0.1 mL) was stirred at room temperature overnight. After workup, the residue was precipitated with cool diethyl ether to afford 16 mg (91%) of **63** as a yellow solid. Mp (decomp at 121 °C). ^1^H NMR (500 MHz, DMSO-*d*_6_) δ: 1.63 (m, 3H, CH_2_CH_2_CO), 1.72 (m, 3H, CH_2_CH_2_CO), 1.95 (t, *J* = 8.6 Hz, 6H, CH_2_CH_2_CO), 2.37 (m, 2H, CH_2_), 2.92 (t, *J* = 5.3 Hz, 2H, CH_2_), 3.11 (dd, *J* = 14.0, 7.3 Hz, 3H, β-CH_2_Trp), 3.25 (dd, *J* = 14.0, 6.9 Hz, 3H, β-CH_2_Trp), 3.48 (dd, *J* = 6.4, 3.6 Hz, 2H, CH_2_), 3.52 (m, 2H, CH_2_), 3.55 (t, *J* = 5.2 Hz, 2H, CH_2_), 3.59 (td, *J* = 6.4, 2.5 Hz, 2H, CH_2_), 3.65 (d, *J* = 5.3 Hz, 2H, α-CH_2_Gly), 4.46 (m, 3H, α-CHTrp), 7.06 (t, *J* = 7.5 Hz, 3H, Ar), 7.14–7.23 (m, 9H, Ar), 7.32 (d, *J* = 8.2 Hz, 3H, Ar), 7.71 (d, *J* = 8.1 Hz, 3H, Ar), 7.98 (t, *J* = 5.5 Hz, 1H, NHCOCH_2_NH), 8.06–8.14 (m, 9H, Ar, NH-Trp), 11.62 (s, 3H, NH-1^*i*^Trp). ^13^C NMR (126 MHz, DMSO-*d*_6_) δ: 27.8 (β-CH_2_Trp), 29.8 (CH_2_CH_2_CO), 30.5 (CH_2_CH_2_CO), 36.2, 39.1, 53.7 (CH_2_), 53.8 (α-CHTrp), 57.3 (C(NHCOCH_2_NH_2_)), 67.1, 69.8, 69.9 (CH_2_), 112.0, 119.7, 119.8, 120.0, 120.1, 123.7, 124.7, 126.2, 127.8, 137.9, 145.4, 148.0 (Ar), 168.4, 171.0 (NHCOCH_2_NHCO), 172.4 (CH_2_CH_2_CO), 173.5 (COOH). HRMS (ESI^+^) *m*/*z*: calcd for C_70_H_72_N_12_O_19_S_3_ 1480.4199; found 1480.4180.

#### Compound **64**

To a solution containing **51** (50 mg, 0.04 mmol), 4,7,10,13-tetraoxohexadecane-1,16-dioic acid (5 mg, 0.02 mmol), and HATU (17 mg, 0.04 mmol) in anhydrous DMF (0.2 mL), DIPEA (15 μL, 0.08 mmol) was added. The resulting mixture was heated to 30 °C for 48 h. Then, it was quenched with a saturated solution of NH_4_Cl (5 mL) and volatiles were removed. The residue was dissolved in ethyl acetate (20 mL) and washed with water (10 mL). The organic layer was dried over Na_2_SO_4_, filtered, and evaporated to dryness, and the residue was purified by CCTLC (DCM/methanol, 25:1). The fractions containing the compound were evaporated, and the residue obtained was purified by CCTLC (ethyl acetate/methanol, 2:1) to yield 33 mg (65%) of **64** as an amorphous yellow solid. ^1^H NMR (500 MHz, DMSO-*d*_6_) δ: 1.59–1.78 (m, 16H, CH_2_CH_2_CO, CH_2_), 1.98 (m, 12H, CH_2_CH_2_CO), 2.36 (t, *J* = 6.6 Hz, 4H, CH_2_), 2.99 (t, *J* = 5.7 Hz, 4H, CH_2_), 3.14 (dd, *J* = 13.8, 6.7 Hz, 6H, β-CH_2_Trp), 3.23 (dd, *J* = 14.1, 7.8 Hz, 6H, β-CH_2_Trp), 3.42 (s, 18H, COOCH_3_), 3.56 (t, *J* = 6.6 Hz, 4H, CH_2_), 3.65 (s, 4H, α-CH_2_Gly), 4.05 (m, 4H, CH_2_), 4.46 (m, 6H, α-CHTrp), 7.07 (t, *J* = 7.6 Hz, 6H, Ar), 7.14–7.23 (m, 18H, Ar), 7.34 (d, *J* = 8.2 Hz, 6H, Ar), 7.62 (d, *J* = 8.0 Hz, 6H, Ar), 8.10 (d, *J* = 8.6 Hz, 12H, Ar), 8.32 (d, *J* = 7.5 Hz, 3H), 11.68 (s, 1H).

#### Compound **65**

Following the general procedure A, a mixture containing compound 64 (33 mg, 0.01 mmol) in THF (0.2 mL) and LiOH·H_2_O (6 mg, 0.13 mmol) in water (0.1 mL) was stirred at room temperature overnight. After workup, the residue was precipitated with cool petroleum ether to afford 26 mg (81%) of 65 as an amorphous yellow solid. ^1^H NMR (500 MHz, DMSO-*d*_6_) δ: 1.67 (m, 12H, CH_2_CH_2_CO), 1.95 (m, 12H, CH_2_CH_2_CO), 2.36 (t, *J* = 6.9 Hz, 4H, CH_2_), 3.12 (dd, *J* = 13.9, 7.1 Hz, 6H, β-CH_2_Trp), 3.25 (dd, *J* = 14.0, 6.9 Hz, 6H, β-CH_2_Trp), 3.37–3.48 (m, 12H, CH_2_), 3.55 (t, *J* = 6.6 Hz, 4H, CH_2_), 3.64 (d, *J* = 4.7 Hz, 4H, α-CH_2_Gly), 4.47 (m, 6H, α-CHTrp), 7.06 (t, *J* = 7.6 Hz, 6H, Ar), 7.14–7.21 (m, 18H, Ar), 7.32 (d, *J* = 8.2 Hz, 6H, Ar), 7.70 (d, *J* = 8.0 Hz, 6H, Ar), 7.95 (t, *J* = 5.5 Hz, 2H, NHCOCH_2_NH), 8.08 (m, 12H, Ar), 8.13 (d, *J* = 8.0 Hz, 6H, NH-Trp), 11.61 (s, 6H, NH-1^*i*^Trp), 12.53 (s, 6H, COOH). ^13^C NMR (126 MHz, DMSO-*d*_6_) δ: 27.7 (β-CH_2_Trp), 29.8 (CH_2_CH_2_CO), 30.5 (CH_2_CH_2_CO), 36.2 (NHCOCH_2_CH_2_O), 53.5 (α-CHTrp), 57.1 (C(NHCOCH_2_NH_2_)), 67.2, 69.9, 70.0, 70.1 (OCH_2_), 112.0, 119.6, 119.8, 120.1, 123.7, 124.7, 126.2, 127.8, 137.9, 145.4, 148.0 (Ar), 168.3 (NHCOCH_2_NH_2_), 170.9 (NHCOCH_2_CH_2_O), 172.4 (CH_2_CH_2_CO), 173.4 (COOH). HRMS (ESI^–^) *m*/*z*: calcd for C_138_H_136_N_22_O_38_S_6_ 2900.7710; found 1450.3800 (M/2).

### Antiviral Screening Using a Pseudotyped-VSV Assay

Pseudotyped vesicular stomatitis virus (VSV) carrying a codon-optimized gene for the S protein of the Wuhan-Hu-1 or the Omicron BA.1 variant SARS-CoV-2 strain and encoding both GFP and firefly luciferase was produced and tittered on Vero cells or A549-Ace2-TMPRSS2 cells (InVivoGen, a549-hace2tpsa) as previously described.^45^ The VSV pseudotyped with the G protein was produced in BHK-G43 cells (Kind gift of Dr. Gert Zimmer).^46^ For antiviral assays ∼1000 focus forming units were premixed with the compounds for 1 h at 37 °C before the addition of the mixture to cells previously plated in a 96-well plate. In all conditions, a mock treatment with the dilutant was included (DMSO). After 16 h, virus infection was quantified by examining GFP fluorescence on a live-cell microscope (Incucyte S3, Sartorius). In the case of drug treatment following infection, cells were infected in the absence of the drug for 1 h, after which the viral inoculum was removed to stop new infections and the drug or control dilutant added. Finally, toxicity was assessed by adding resazurin (Sigma-Aldrich, R7017) to a final concentration of 44 μM in the same wells where virus infection was assessed, incubation for 1 h at 37 °C, and reading fluorescence on a Tecan Spark microplate reader with an excitation of 535 nm and emission of 595 nm. All compounds were tested in triplicate and toxicity or antiviral activity derived by comparison to control treated cells. The dose resulting in a 50% reduction of virus-produced GFP signal (IC_50_) was calculated using the drc package in R using a three-parameter log-logistic function (LL3).

### Antiviral Activity Using SARS-CoV-2

A SARS-CoV-2 isolate was kindly provided by Sonia Zúñiga, Isabel Sola and Luis Enjuanes from the Spanish National Centre for Biotechnology (CNB-CSIC). The isolate harbored the following mutations relative to the Wuhan-Hu-1 reference strain (GenBank MN908947): C3037>T, resulting in a silent mutation, C14408>T in nsp12, and A23403>G (D614G in the S protein). All the infections were carried out at the Biosafety Level 3 (BSL-3) Facility of the Fundación para Fomento de Investigación Sanitaria y Biomédica (FISABIO) in Valencia, Spain. The virus was amplified at a multiplicity of infection of 0.001 for 60 h on Vero-E6 cells.

For the SARS-CoV-2 virus production assay, 10^5^ Median Tissue Culture Infectious Dose (TCID50) of the virus were incubated with 10 μM of the compounds or dilutant for 1 h, after which the mix was used to infect VeroE6-TMPRSS2 cells (Japanese Collection of Research Bioresources; JCRB1819) or A549-ACE2 cells^47^ for 1 h. Subsequently, the inoculum was removed and fresh media with compound or dilutant was added. After 24 h, the supernatant was collected and tittered via limited dilution on VeroE6-TMPRSS2 cells. The data are presented as the relative amount of virus-produced versus mock-treated controls.

### Production of Proteins

RBD_Wuhan_ was produced in a system of baculovirus/insect cells and purified as reported^48^ (we thank these authors for providing the plasmid). The protein (residues Arg319-Phe541 of S_Wuhan_) hosts a C-terminal 6×His-tag and is exported to the culture medium thanks to a gp67 signal peptide that is cleaved upon exportation.^49^

For use in MST experiments, we produced the S_Wuhan_ Hexapro as reported^50^ from the same plasmid used by these authors (supplied by Addgene, https://addgene.org, catalog #154754). The protein is the ectodomain (amino acids 1-1208) of S_Wuhan_ (GenBank: NC_045512.2 reference sequence), modified to eliminate the furin cleavage site (^682^RRAR^685^>^682^GSAS^685^) and to include six amino acid replacements by proline (F817, A892, A899, A942, K986, and V987) to stabilize the protein, increasing much production efficiency.^50^ In addition, to restore the trimeric nature of the viral protein, the sequence is C-terminally linked to a T4 fibritin trimerization motif (foldon) followed by an HRV3C protease cleavage site preceding 8×His and TwinStrep tags in sequence. Expression from the plasmid and purification were carried out as reported^50^ except for the following changes: (1) production in Expi293F cells (from Thermo Fisher Scientific); (2) transfection with poly-ethylenimine (PEI MAX Polyethylenimine Hydrochloride MW 40000; Polysciences Europe GmbH); 3) replacement of the Strep-tag-based affinity chromatography by 8×His-tag-based affinity chromatography (5-mL HisTrapTM Excell column; Cytiva, Madrid, Spain) in the first purification step; and (4) utilization in the final size-exclusion chromatography of a solution of 10 mM sodium-Hepes pH 7.2, 150 mM NaCl.

For cryoEM studies, the S protein was produced in baculovirus/insect cells exactly as described in our prior structural work on this protein, utilizing the form that incorporates the presently universal D614G mutation.^42^ In this approach, allowance for intrinsic S protein flexibility and movements of its RBD domains is prioritized over yield of pure protein.^50^ Thus, only two residues (K986 and V987) are replaced by proline while the furin cleavage site is also eliminated (change ^682^RRAR^685^>A). The protein also includes C-terminal trimerization foldon and 9×His and Mic tags.

The N-terminal peptidase domain of human ACE2 (residues Ser19-Asp615) was produced as previously reported by our laboratory.^42^

Purified proteins were concentrated by centrifugal ultrafiltration (Amicon Ultra Millipore devices holding membranes of 10, 30, or 100 kDa nominal cutoff, for, respectively, RBD, ACE2, and S proteins) and were stored at −80 °C. They were quantified spectrophotometrically at 280 nm, using sequence-deduced (EXPASY Protparam tool, https://www.expasy.org/) mass extinction coefficients of 13.7 for RBDs, 10.2 for S, and 21.8 for ACE2.

### Thermal Shift Assays

Thermofluor assays^38^ were performed in 20 μL of a solution of 17 μg/mL SARS-CoV-2 RBD in 10 mM Na Hepes pH 7.3, 150 mM NaCl, a 1:1000 dilution of the commercial preparation of SYPRO Orange from Invitrogen (Carlsbad, CA) and 5% dimethyl sulfoxide alone or as the solvent carrying into the mixture the compound of interest to a final concentration of 0.1 mM. The SYPRO Orange was the last component added, following a 10-min incubation at 21 °C of the mixture without this fluorofore. Then, the mixtures were transferred to wells in a microwell plate, sealed with tape, and placed in a real-time PCR instrument (CFX Opus 96 Real-Time PCR System, Biorad, Hercules, CA, USA), which was used to monitor the increase in SYPRO Orange fluorescence (excitation at 470 nm; emission at 570 nm) with a temperature increase at a ramp of 1 °C/min. Each assay with three replicate wells for each point was repeated at least two times on different days. Plots, curve fittings, and numerical calculations were performed with the program Graphpad Prism 7 (GraphPad Software, San Diego, CA, USA).

### Microscale Thermophoresis (MST)

Poly-His-tagged SARS-CoV-2 RBD or S proteins (see above) were labeled using His-Tag Labeling Kit RED-tris-NTA 2nd Generation (NanoTemper Technologies, München, Germany) according to the manufacturer’s instructions. Briefly, equal volumes of 200 nM (as protein chains) target protein and of 100 nM dye in phosphate buffered saline pH 7.1 (PBS; provided in the kit) were mixed and incubated 30 min at 21 °C prior to 10-min centrifugation at 15000×*g*. The supernatant was used in the MST assays, by mixing with an equal volume of the other components. The final mixture contained 50 nM nominal concentration of labeled protein chains, PBS pH 7.1, 0.05% Tween-20, 5% dimethyl sulfoxide (DMSO), and the indicated concentrations of each compound tested (decreasing in 16 2-fold dilution steps from 5 mM). The 16 mixtures were incubated 15 min at 21 °C before being used to fill the 16 corresponding Monolith Capillaries (catalog number MO-K022) composing a full run of MST measurements, carried out in a Monolith NT.115 apparatus (NanoTemper Technologies). In these measurements, the fluorescence profile is registered for several seconds before turning on the infrared laser, then for 21 s from the moment the infrared laser turns on, and finally for 4 s after the laser turns off (to corroborate the return of the fluorescence toward the initial values). The results were analyzed using a dedicated software (MO.Control software for affinity analysis; NanoTemper Technologies).

The binding of the catalytic domain of ACE2 to the labeled RBD and S proteins was tested in the same way, except for the use of decreasing concentrations of ACE2 (tag-less), in 2-fold dilution steps, from an initial concentration of 5 μM (as ACE2 chains). The titrations were done in the absence and in the presence of 0.5 mM compound **2** or **65**.

### Cryo-Electron Microscopy Sample Preparation

#### Cryo-EM Data Acquisition

The complex of S (see the section on Production of Proteins) with compound **2** (Na salt) was prepared by incubating 5 min at 25 °C 0.5 mg/mL S and 0.3 mM compound **2** in 0.5 mM, Hepes pH 7.2, 150 mM NaCl. Then, the mixture was placed at 4 °C, and 3 μL aliquots were placed on cryo-EM grids (QUANTIFOIL R 1.2/1.3 Au:300-mesh grids) that had been made hydrophilic by glow discharge (30 s using a Leica EM ACE600 device). The grids were then placed in the blotting chamber of a Leica EM GP2 at 10 °C and 95% ambient humidity and immediately vitrified in liquid-N_2_-cooled liquid ethane.

Transmission cryoEM images were collected automatically (EPU Automated Data Acquisition Software for Single Particle Analysis; Thermo Fisher Scientific) on a FEI Talos electron microscope operated at 200 kV under low-dose conditions and images recorded on a FEI Falcon III detector operating in electron counting mode, at −1 to −2.5 μm defocus. A total of 3500 movies were recorded at a calibrated magnification of 120,000×, yielding a pixel size of 0.85 Å on the specimen. Each movie comprises 60 frames with an exposure rate of 0.55 e^–^/Å^2^ per frame, with a total exposure time of 28 s and an accumulated exposure of 30 e^–^/Å^2^.

#### Image Processing

All image processing steps were performed using programs within Scipion.^51^ Movies were motion-corrected and dose weighted with RELION-Motion Correction.^52^ Aligned, nondose-weighted micrographs were then used to estimate the contrast transfer function (CTF) with GCTF.^53^ 662,994 particles were then automatically picked using Gautomatch^53^ (https://www2.mrc-lmb.cam.ac.uk/download/gautomatch-056/) and were 2D-classified with cryoSPARC,^54^ leading to the selection of 375,927 particles. The cryoSPARC initial model protocol was then used with a subset of the total particles to classify the particles into 4 classes. The best generated class (of around 50% of the particles) was uniformly refined in cryoSPARC using 3-fold-symmetry and then used as initial model for 3D heterogeneous refinement in cryoSPARC without applying symmetry to isolate two distinct particle classes, one (278,833 particles) for the spike trimer with 1 RBD domain up and 2 RBDs down, and another one (97,094 particles) with all three RBD domains down. We subsequently refined the maps obtained from these two groups of particles to 3.4 and 4.3 Å resolutions, respectively, based on the gold-standard criterion (FSC = 0.143). The resulting maps were sharpened with DeepEMhancer.^55^

#### Model Building and Refinement

For model building of the spike in either the map in the 1-up or the 3-down conformation, we started with a deposited PDB file for the S protein in the 1-up conformation of the D614G variant (PDB: 7QDG). After fitting of 7QDG to the map using Chimera,^56^ followed by rigid-body docking with Coot^57^ of different regions of each monomer for keeping the secondary structure of the spike (residues 14-293; 294-319 plus 592-699; 320-330; 331-529 plus 530-591; 734-775; 944-962; 963-989; 990-1027), further model was built manually in Coot, followed by several rounds of refinement using REFMAC,^58^ paying special attention to the contact region between different RBDs. In one of the subunits of the 3-down map, an extra density was found having a size compatible with that of compound **2** (see Results section). This process was repeated until acceptable refinement metrics were obtained.

## Data Availability

Electron microscopy maps have been deposited in the Electron Microscopy Data Bank (EMDB, https://www.ebi.ac.uk/emdb/) with accession codes EMD-17576 and EMD-17578, for the 1-up and 3-down forms, respectively of S:D614G. The corresponding atomic coordinates for these two forms are deposited in the PDB with accession codes 8P99 and 8P9Y, respectively.

## Supporting Information Available

Synthesis and spectroscopic data of disulfides **20a**–**c**; (Figure S1) antiviral dose–response profiles measured using the VSV-S assay in both Vero E6 and A549-Ace2-TMPRSS2; (Figure S2) correlation between the IC_50_ values for each compound observed in Vero E6 and A549-ACE2-TMPRSS2 cells obtained with the VSV-S antiviral assay; and ^1^H, ^13^C NMR spectra and HPLC chromatograms of selected compounds (PDF)
Molecular formula strings of the novel synthesized compounds (CSV)

## Abbreviations Used

MST: microscale thermophoresis
VSV-S: pseudotyped vesicular stomatitis virus expressing the S protein of SARS-CoV-2

## References

[ref1] Zhu N.; Zhang D.; Wang W.; Li X.; Yang B.; Song J.; Zhao X.; Huang B.; Shi W.; Lu R.; Niu P.; Zhan F.; Ma X.; Wang D.; Xu W.; Wu G.; Gao G. F.; Tan W. A Novel Coronavirus from Patients with Pneumonia in China, 2019. N. Engl. J. Med. 2020, 382, 727–733. 10.1056/NEJMoa2001017. 31978945 PMC7092803

[ref2] Matheson N. J.; Lehner P. J. How Does SARS-CoV-2 Cause COVID-19?. Science 2020, 369, 510–511. 10.1126/science.abc6156. 32732413

[ref3] Fung T. S.; Liu D. X. Human Coronavirus: Host-Pathogen Interaction. Annu. Rev. Microbiol. 2019, 73, 529–557. 10.1146/annurev-micro-020518-115759. 31226023

[ref4] Petrosillo N.; Viceconte G.; Ergonul O.; Ippolito G.; Petersen E. COVID-19, SARS and MERS: Are They Closely Related?. Clin. Microbiol. Infect. 2020, 26, 729–734. 10.1016/j.cmi.2020.03.026. 32234451 PMC7176926

[ref5] Siegel D.; Hui H. C.; Doerffler E.; Clarke M. O.; Chun K.; Zhang L.; Neville S.; Carra E.; Lew W.; Ross B.; Wang Q.; Wolfe L.; Jordan R.; Soloveva V.; Knox J.; Perry J.; Perron M.; Stray K. M.; Barauskas O.; Feng J. Y.; Xu Y.; Lee G.; Rheingold A. L.; Ray A. S.; Bannister R.; Strickley R.; Swaminathan S.; Lee W. A.; Bavari S.; Cihlar T.; Lo M. K.; Warren T. K.; Mackman R. L. Discovery and Synthesis of a Phosphoramidate Prodrug of a Pyrrolo[2,1-f][Triazin-4-Amino] Adenine C-Nucleoside (GS-5734) for the Treatment of Ebola and Emerging Viruses. J. Med. Chem. 2017, 60, 1648–1661. 10.1021/acs.jmedchem.6b01594. 28124907

[ref6] Beigel J. H.; Tomashek K. M.; Dodd L. E.; Mehta A. K.; Zingman B. S.; Kalil A. C.; Hohmann E.; Chu H. Y.; Luetkemeyer A.; Kline S.; de Castilla D. L.; Finberg R. W.; Dierberg K.; Tapson V.; Hsieh L.; Patterson T. F.; Paredes R.; Sweeney D. A.; Short W. R.; Touloumi G.; Lye D. C.; Ohmagari N.; Oh M.-D.; Ruiz-Palacios G. M.; Benfield T.; Fätkenheuer G.; Kortepeter M. G.; Atmar R. L.; Creech C. B.; Lundgren J.; Babiker A. G.; Pett S.; Neaton J. D.; Burgess T. H.; Bonnett T.; Green M.; Makowski M.; Osinusi A.; Nayak S.; Lane H. C.; Remdesivir for the Treatment of COVID-19 — Final Report. N. Engl. J. Med. 2020, 383, 1813–1826. 10.1056/NEJMoa2007764. 32445440 PMC7262788

[ref7] Painter G. R.; Natchus M. G.; Cohen O.; Holman W.; Painter W. P. Developing a Direct Acting, Orally Available Antiviral Agent in a Pandemic: The Evolution of Molnupiravir as a Potential Treatment for COVID-19. Curr. Opin. Virol. 2021, 50, 17–22. 10.1016/j.coviro.2021.06.003. 34271264 PMC8277160

[ref8] Owen D. R.; Allerton C. M. N.; Anderson A. S.; Aschenbrenner L.; Avery M.; Berritt S.; Boras B.; Cardin R. D.; Carlo A.; Coffman K. J.; Dantonio A.; Di L.; Eng H.; Ferre R.; Gajiwala K. S.; Gibson S. A.; Greasley S. E.; Hurst B. L.; Kadar E. P.; Kalgutkar A. S.; Lee J. C.; Lee J.; Liu W.; Mason S. W.; Noell S.; Novak J. J.; Obach R. S.; Ogilvie K.; Patel N. C.; Pettersson M.; Rai D. K.; Reese M. R.; Sammons M. F.; Sathish J. G.; Singh R. S. P.; Steppan C. M.; Stewart A. E.; Tuttle J. B.; Updyke L.; Verhoest P. R.; Wei L.; Yang Q.; Zhu Y. An Oral SARS-CoV-2 M pro Inhibitor Clinical Candidate for the Treatment of COVID-19. Science 2021, 374, 1586–1593. 10.1126/science.abl4784. 34726479

[ref9] Zhou Y.; Simmons G. Development of Novel Entry Inhibitors Targeting Emerging Viruses. Expert Rev. Anti-infect. Ther. 2012, 10, 1129–1138. 10.1586/eri.12.104. 23199399 PMC3587779

[ref10] Yang H.; Rao Z. Structural Biology of SARS-CoV-2 and Implications for Therapeutic Development. Nat. Rev. Microbiol. 2021, 19, 685–700. 10.1038/s41579-021-00630-8. 34535791 PMC8447893

[ref11] Walls A. C.; Park Y.-J.; Tortorici M. A.; Wall A.; McGuire A. T.; Veesler D. Structure, Function, and Antigenicity of the SARS-CoV-2 Spike Glycoprotein. Cell 2020, 181, 281–292.e6. 10.1016/j.cell.2020.02.058. 32155444 PMC7102599

[ref12] Jackson C. B.; Farzan M.; Chen B.; Choe H. Mechanisms of SARS-CoV-2 Entry into Cells. Nat. Rev. Mol. Cell Biol. 2022, 23, 3–20. 10.1038/s41580-021-00418-x. 34611326 PMC8491763

[ref13] Weinreich D. M.; Sivapalasingam S.; Norton T.; Ali S.; Gao H.; Bhore R.; Musser B. J.; Soo Y.; Rofail D.; Im J.; Perry C.; Pan C.; Hosain R.; Mahmood A.; Davis J. D.; Turner K. C.; Hooper A. T.; Hamilton J. D.; Baum A.; Kyratsous C. A.; Kim Y.; Cook A.; Kampman W.; Kohli A.; Sachdeva Y.; Graber X.; Kowal B.; DiCioccio T.; Stahl N.; Lipsich L.; Braunstein N.; Herman G.; Yancopoulos G. D. REGN-COV2, a Neutralizing Antibody Cocktail, in Outpatients with Covid-19. N. Engl. J. Med. 2021, 384, 238–251. 10.1056/NEJMoa2035002. 33332778 PMC7781102

[ref14] Cox M.; Peacock T. P.; Harvey W. T.; Hughes J.; Wright D. W.; Willett B. J.; Thomson E.; Gupta R. K.; Peacock S. J.; Robertson D. L.; Carabelli A. M. SARS-CoV-2 Variant Evasion of Monoclonal Antibodies Based on in Vitro Studies. Nat. Rev. Microbiol. 2023, 21, 112–124. 10.1038/s41579-022-00809-7. 36307535 PMC9616429

[ref15] Flaxman A. D.; Issema R.; Barnabas R. V.; Ross J. M. Estimated Health Outcomes and Costs of COVID-19 Prophylaxis With Monoclonal Antibodies Among Unvaccinated Household Contacts in the US. JAMA Network Open 2022, 5, e228632 10.1001/jamanetworkopen.2022.8632. 35452104 PMC9034404

[ref16] Rivero-Buceta E.; Doyagüez E. G.; Colomer I.; Quesada E.; Mathys L.; Noppen S.; Liekens S.; Camarasa M.-J.; Pérez-Pérez M.-J.; Balzarini J.; San-Félix A. Tryptophan Dendrimers That Inhibit HIV Replication, Prevent Virus Entry and Bind to the HIV Envelope Glycoproteins Gp120 and Gp41. Eur. J. Med. Chem. 2015, 106, 34–43. 10.1016/j.ejmech.2015.10.031. 26513643

[ref17] Rivero-Buceta E.; Sun L.; Martínez-Gualda B.; Doyagüez E. G.; Donckers K.; Quesada E.; Camarasa M.-J.; Delang L.; San-Félix A.; Neyts J.; Leyssen P. Optimization of a Class of Tryptophan Dendrimers That Inhibit HIV Replication Leads to a Selective, Specific, and Low-Nanomolar Inhibitor of Clinical Isolates of Enterovirus A71. Antimicrob. Agents Chemother. 2016, 60, 5064–5067. 10.1128/AAC.00626-16. 27246775 PMC4958208

[ref18] Martínez-Gualda B.; Sun L.; Rivero-Buceta E.; Flores A.; Quesada E.; Balzarini J.; Noppen S.; Liekens S.; Schols D.; Neyts J.; Leyssen P.; Mirabelli C.; Camarasa M.-J.; San-Félix A. Structure-Activity Relationship Studies on a Trp Dendrimer with Dual Activities against HIV and Enterovirus A71. Modifications on the Amino Acid. Antiviral Res. 2017, 139, 32–40. 10.1016/j.antiviral.2016.12.010. 28017762

[ref19] Martí-Marí O.; Martínez-Gualda B.; de la Puente-Secades S.; Mills A.; Quesada E.; Abdelnabi R.; Sun L.; Boonen A.; Noppen S.; Neyts J.; Schols D.; Camarasa M.-J.; Gago F.; San-Félix A. Double Arylation of the Indole Side Chain of Tri- and Tetrapodal Tryptophan Derivatives Renders Highly Potent HIV-1 and EV-A71 Entry Inhibitors. J. Med. Chem. 2021, 64, 10027–10046. 10.1021/acs.jmedchem.1c00315. 34229438 PMC8389807

[ref20] Martínez-Gualda B.; Sun L.; Martí-Marí O.; Mirabelli C.; Delang L.; Neyts J.; Schols D.; Camarasa M.-J.; San-Félix A. Modifications in the Branched Arms of a Class of Dual Inhibitors of HIV and EV71 Replication Expand Their Antiviral Spectrum. Antiviral Res. 2019, 168, 210–214. 10.1016/j.antiviral.2019.06.006. 31228490 PMC7114229

[ref21] Martínez-Gualda B.; Sun L.; Martí-Marí O.; Noppen S.; Abdelnabi R.; Bator C. M.; Quesada E.; Delang L.; Mirabelli C.; Lee H.; Schols D.; Neyts J.; Hafenstein S.; Camarasa M.-J.; Gago F.; San-Félix A. Scaffold Simplification Strategy Leads to a Novel Generation of Dual Human Immunodeficiency Virus and Enterovirus-A71 Entry Inhibitors. J. Med. Chem. 2020, 63, 349–368. 10.1021/acs.jmedchem.9b01737. 31809045

[ref22] Fikatas A.; Vervaeke P.; Martínez-Gualda B.; Martí-Marí O.; Noppen S.; Meyen E.; Camarasa M.-J.; San-Félix A.; Pannecouque C.; Schols D. Tryptophan Trimers and Tetramers Inhibit Dengue and Zika Virus Replication by Interfering with Viral Attachment Processes. Antimicrob. Agents Chemother. 2020, 64, 10–128. 10.1128/AAC.02130-19. PMC7038274 31932383

[ref23] Sun L.; Lee H.; Thibaut H. J.; Lanko K.; Rivero-Buceta E.; Bator C.; Martinez-Gualda B.; Dallmeier K.; Delang L.; Leyssen P.; Gago F.; San-Félix A.; Hafenstein S.; Mirabelli C.; Neyts J.; San-Félix A.; Hafenstein S.; Mirabelli C.; Neyts J. Viral Engagement with Host Receptors Blocked by a Novel Class of Tryptophan Dendrimers That Targets the 5-Fold-Axis of the Enterovirus - A 71 Capsid. PLoS Pathog. 2019, 15, e1007760 10.1371/journal.ppat.1007760. 31071193 PMC6590834

[ref24] Shao L.; Yang F.; Su Y.; Li W.; Zhang J.; Xu H.; Huang B.; Sun M.; Mu Y.; Zhang Y.; Yu F. Design and Synthesis of Oleanolic Acid Trimers to Enhance Inhibition of Influenza Virus Entry. ACS Med. Chem. Lett. 2021, 12, 1759–1765. 10.1021/acsmedchemlett.1c00374. 34795865 PMC8591716

[ref25] Schubertová V.; Martinez-Veracoechea F. J.; Vácha R. Design of Multivalent Inhibitors for Preventing Cellular Uptake. Sci. Rep. 2017, 7, 11689 10.1038/s41598-017-11735-7. 28916832 PMC5601900

[ref26] Giménez E.; Albert E.; Zulaica J.; Torres I.; Rusu L.; Moreno A. R.; Burgos J. S.; Peiró S.; Salas D.; Vanaclocha H.; Limón R.; Alcaraz M. J.; Sánchez-Payá J.; Díez-Domingo J.; Comas I.; Gonzáles-Candelas F.; Geller R.; Navarro D.; Burgos J. S.; Meneu de Guillerna R.; Vanaclocha L. H.; Burks D. J.; Cervantes A.; Comas I.; Díez-Domingo J.; Peiro S.; González-Candelas F.; Ferrer A. C.; Hernández-Aguado I.; Oliver R. N.; Sánchez-Payá J.; Vento T. M.; Zapater L. E.; Navarro D. Severe Acute Respiratory Syndrome Coronavirus 2 Adaptive Immunity in Nursing Home Residents Following a Third Dose of the Comirnaty Coronavirus Disease 2019 Vaccine. Clin. Infect. Dis. 2022, 75, e865–e868. 10.1093/cid/ciac223. 35314856 PMC9129117

[ref27] Weissenborn M. J.; Castangia R.; Wehner J. W.; Šardzík R.; Lindhorst T. K.; Flitsch S. L. Oxo-Ester Mediated Native Chemical Ligation on Microarrays: An Efficient and Chemoselective Coupling Methodology. Chem. Commun. 2012, 48, 4444–4446. 10.1039/c2cc30844d. 22456682

[ref28] He Y.; Jiang J.; Bao W.; Deng W.; Xiang J. TBAI-Mediated Regioselective Sulfenylation of Indoles with Sulfonyl Chlorides in One Pot. Tetrahedron Lett. 2017, 58, 4583–4586. 10.1016/j.tetlet.2017.10.040.

[ref29] Richard D. J.; Schiavi B.; Joullié M. M. Synthetic Studies of Roquefortine C: Synthesis of Isoroquefortine C and a Heterocycle. Proc. Natl. Acad. Sci. 2004, 101, 11971–11976. 10.1073/PNAS.0401407101. 15141083 PMC514418

[ref30] Chen S. Q.; Wang Q. M.; Xu P. C.; Ge S. P.; Zhong P.; Zhang X. H. Iodine-Promoted Selective 3-Selanylation and 3-Sulfenylation of Indoles with Dichalcogenides under Mild Conditions. Phosphorus, Sulfur Silicon Relat. Elem. 2016, 191, 100–103. 10.1080/10426507.2014.999068.

[ref31] Rahaman R.; Devi N.; Bhagawati J. R.; Barman P. Microwave-Assisted Regioselective Sulfenylation of Indoles under Solvent- and Metal-Free Conditions. RSC Adv. 2016, 6, 18929–18935. 10.1039/c5ra26425a.

[ref32] Zheng Y.; Qing F.-L.; Huang Y.; Xu X.-H. Tunable and Practical Synthesis of Thiosulfonates and Disulfides from Sulfonyl Chlorides in the Presence of Tetrabutylammonium Iodide. Adv. Synth. Catal. 2016, 358, 3477–3481. 10.1002/adsc.201600633.

[ref33] Yang Z.; Shi Y.; Zhan Z.; Zhang H.; Xing H.; Lu R.; Zhang Y.; Guan M.; Wu Y. Sustainable Electrocatalytic Oxidant-Free Syntheses of Thiosulfonates from Thiols. ChemElectroChem 2018, 5, 3619–3623. 10.1002/celc.201801058.

[ref34] Habibi A.; Baghersad M. H.; Bilabary M.; Valizadeh Y. Dithioates of Meldrum’s Acid, Dimedone, and Barbituric Acid, Novel Sulfur Transfer Reagents for the One-Pot Copper-Catalyzed Conversion of Aryl Iodides into Diaryl Disulfides. Tetrahedron Lett. 2016, 57, 559–562. 10.1016/j.tetlet.2015.12.085.

[ref35] Preciado S.; Mendive-Tapia L.; Albericio F.; Lavilla R. Synthesis of C-2 Arylated Tryptophan Amino Acids and Related Compounds through Palladium-Catalyzed C–H Activation. J. Org. Chem. 2013, 78, 8129–8135. 10.1021/jo400961x. 23865986

[ref36] Muratov A. V.; Eresko A. B.; Tolkunov V. S.; Tolkunov S. V. 1-Substituted 5,10-Dihydro[1,2]Diazepino[4,5-b]Indol-4(3H)-Ones. Synthesis and Functionalization. Russ. J. Org. Chem. 2019, 55, 345–350. 10.1134/S1070428019030126.

[ref37] Panarese J. D.; Waters S. P. Room-Temperature Aromatization of Tetrahydro-β-Carbolines by 2-Iodoxybenzoic Acid: Utility in a Total Synthesis of Eudistomin U. Org. Lett. 2010, 12, 4086–4089. 10.1021/ol101688x. 20715768 PMC2937063

[ref38] Vedadi M.; Niesen F. H.; Allali-Hassani A.; Fedorov O. Y.; Finerty P. J. Jr.; Wasney G. A.; Yeung R.; Arrowsmith C.; Ball L. J.; Berglund H.; Hui R.; Marsden B. D.; Nordlund P.; Sundstrom M.; Weigelt J.; Edwards A. M. Chemical Screening Methods to Identify Ligands That Promote Protein Stability, Protein Crystallization, and Structure Determination. Proc. Natl. Acad. Sci. U. S. A. 2006, 103, 15835–15840. 10.1073/pnas.0605224103. 17035505 PMC1595307

[ref39] Wienken C. J.; Baaske P.; Rothbauer U.; Braun D.; Duhr S. Protein-Binding Assays in Biological Liquids Using Microscale Thermophoresis. Nat. Commun. 2010, 1, 100 10.1038/ncomms1093. 20981028

[ref40] Xu C.; Wang Y.; Liu C.; Zhang C.; Han W.; Hong X.; Wang Y.; Hong Q.; Wang S.; Zhao Q.; Wang Y.; Yang Y.; Chen K.; Zheng W.; Kong L.; Wang F.; Zuo Q.; Huang Z.; Cong Y. Conformational Dynamics of SARS-CoV-2 Trimeric Spike Glycoprotein in Complex with Receptor ACE2 Revealed by Cryo-EM. Sci. Adv. 2021, 7, eabe5575 10.1126/sciadv.abe5575. 33277323 PMC7775788

[ref41] Benton D. J.; Wrobel A. G.; Xu P.; Roustan C.; Martin S. R.; Rosenthal P. B.; Skehel J. J.; Gamblin S. J. Receptor Binding and Priming of the Spike Protein of SARS-CoV-2 for Membrane Fusion. Nature 2020, 588, 327–330. 10.1038/s41586-020-2772-0. 32942285 PMC7116727

[ref42] Ginex T.; Marco-Marín C.; Wieczór M.; Mata C. P.; Krieger J.; Ruiz-Rodriguez P.; López-Redondo M. L.; Francés-Gómez C.; Melero R.; Sánchez-Sorzano C. Ó.; Martínez M.; Gougeard N.; Forcada-Nadal A.; Zamora-Caballero S.; Gozalbo-Rovira R.; Sanz-Frasquet C.; Arranz R.; Bravo J.; Rubio V.; Marina A.; Geller R.; Comas I.; Gil C.; Coscolla M.; Orozco M.; Llácer J. L.; Carazo J.-M. The Structural Role of SARS-CoV-2 Genetic Background in the Emergence and Success of Spike Mutations: The Case of the Spike A222V Mutation. PLoS Pathog. 2022, 18, e1010631 10.1371/journal.ppat.1010631. 35816514 PMC9302720

[ref43] Yang T.-J.; Yu P.-Y.; Chang Y.-C.; Hsu S.-T. D. D614G Mutation in the SARS-CoV-2 Spike Protein Enhances Viral Fitness by Desensitizing It to Temperature-Dependent Denaturation. J. Biol. Chem. 2021, 297, 101238 10.1016/j.jbc.2021.101238. 34563540 PMC8460419

[ref44] Zhang J.; Cai Y.; Xiao T.; Lu J.; Peng H.; Sterling S. M.; Walsh R. M. Jr.; Rits-Volloch S.; Zhu H.; Woosley A. N.; Yang W.; Sliz P.; Chen B. Structural Impact on SARS-CoV-2 Spike Protein by D614G Substitution. Science 2021, 372, 525–530. 10.1126/science.abf2303. 33727252 PMC8139424

[ref45] Camacho J.; Giménez E.; Albert E.; Zulaica J.; Álvarez-Rodríguez B.; Torres I.; Rusu L.; Burgos J. S.; Peiró S.; Vanaclocha H.; Limón R.; Alcaraz M. J.; Sánchez-Payá J.; Díez-Domingo J.; Comas I.; Gonzáles-Candelas F.; Geller R.; Navarro D.; Cumulative Incidence of SARS-CoV-2 Infection in the General Population of the Valencian Community (Spain) after the Surge of the Omicron BA.1 Variant. J. Med. Virol. 2023, 95, e28284 10.1002/jmv.28284. 36333837 PMC9828341

[ref46] Berger Rentsch M.; Zimmer G. A Vesicular Stomatitis Virus Replicon-Based Bioassay for the Rapid and Sensitive Determination of Multi-Species Type I Interferon. PLoS One 2011, 6, e25858 10.1371/journal.pone.0025858. 21998709 PMC3187809

[ref47] Buchrieser J.; Dufloo J.; Hubert M.; Monel B.; Planas D.; Rajah M. M.; Planchais C.; Porrot F.; Guivel-Benhassine F.; der Werf S.; Casartelli N.; Mouquet H.; Bruel T.; Schwartz O. Syncytia Formation by SARS-CoV-2-Infected Cells. EMBO J. 2020, 39, e106267 10.15252/embj.2020106267. 33051876 PMC7646020

[ref48] Lan J.; Ge J.; Yu J.; Shan S.; Zhou H.; Fan S.; Zhang Q.; Shi X.; Wang Q.; Zhang L.; Wang X. Structure of the SARS-CoV-2 Spike Receptor-Binding Domain Bound to the ACE2 Receptor. Nature 2020, 581, 215–220. 10.1038/s41586-020-2180-5. 32225176

[ref49] Deshpande C. N.; Xin V.; Lu Y.; Savage T.; Anderson G. J.; Jormakka M. Large Scale Expression and Purification of Secreted Mouse Hephaestin. PLoS One 2017, 12, e0184366 10.1371/journal.pone.0184366. 28880952 PMC5589216

[ref50] Hsieh C.; Goldsmith J. A.; Schaub J. M.; DiVenere A. M.; Kuo H.-C.; Javanmardi K.; Le K. C.; Wrapp D.; Lee A. G.; Liu Y.; Chou C.-W.; Byrne P. O.; Hjorth C. K.; Johnson N. V.; Ludes-Meyers J.; Nguyen A. W.; Park J.; Wang N.; Amengor D.; Lavinder J. J.; Ippolito G. C.; Maynard J. A.; Finkelstein I. J.; McLellan J. S. Structure-Based Design of Prefusion-Stabilized SARS-CoV-2 Spikes. Science 2020, 369, 1501–1505. 10.1126/science.abd0826. 32703906 PMC7402631

[ref51] de la Rosa-Trevín J. M.; Quintana A.; del Cano L.; Zaldívar A.; Foche I.; Gutiérrez J.; Gómez-Blanco J.; Burguet-Castell J.; Cuenca-Alba J.; Abrishami V.; Vargas J.; Otón J.; Sharov G.; Vilas J. L.; Navas J.; Conesa P.; Kazemi M.; Marabini R.; Sorzano C. O. S.; Carazo J. M. Scipion: A Software Framework toward Integration, Reproducibility and Validation in 3D Electron Microscopy. J. Struct. Biol. 2016, 195, 93–99. 10.1016/j.jsb.2016.04.010. 27108186

[ref52] Zivanov J.; Nakane T.; Scheres S. H. W. A Bayesian Approach to Beam-Induced Motion Correction in Cryo-EM Single-Particle Analysis. IUCrJ 2019, 6, 5–17. 10.1107/S205225251801463X. PMC6327179 30713699

[ref53] Zhang K. Gctf: Real-Time CTF Determination and Correction. J. Struct. Biol. 2016, 193, 1–12. 10.1016/j.jsb.2015.11.003. 26592709 PMC4711343

[ref54] Punjani A.; Rubinstein J. L.; Fleet D. J.; Brubaker M. A. CryoSPARC: Algorithms for Rapid Unsupervised Cryo-EM Structure Determination. Nat. Methods 2017, 14, 290–296. 10.1038/nmeth.4169. 28165473

[ref55] Sanchez-Garcia R.; Gomez-Blanco J.; Cuervo A.; Carazo J. M.; Sorzano C. O. S.; Vargas J. DeepEMhancer: A Deep Learning Solution for Cryo-EM Volume Post-Processing. Commun. Biol. 2021, 4, 874 10.1038/s42003-021-02399-1. 34267316 PMC8282847

[ref56] Pettersen E. F.; Goddard T. D.; Huang C. C.; Couch G. S.; Greenblatt D. M.; Meng E. C.; Ferrin T. E. UCSF Chimera?A Visualization System for Exploratory Research and Analysis. J. Comput. Chem. 2004, 25, 1605–1612. 10.1002/jcc.20084. 15264254

[ref57] Emsley P.; Lohkamp B.; Scott W. G.; Cowtan K. Features and Development of Coot. Acta Crystallogr., Sect. D: Biol. Crystallogr. 2010, 66, 486–501. 10.1107/S0907444910007493. 20383002 PMC2852313

[ref58] Brown A.; Long F.; Nicholls R. A.; Toots J.; Emsley P.; Murshudov G. Tools for Macromolecular Model Building and Refinement into Electron Cryo-Microscopy Reconstructions. Acta Crystallogr., Sect. D: Biol. Crystallogr. 2015, 71, 136–153. 10.1107/S1399004714021683. 25615868 PMC4304694

