# Supplementary material for: C-2 Thiophenyl Tryptophan Trimers Inhibit Cellular Entry of SARS-CoV-2 through Interaction with the Viral Spike (S) Protein
Source: J Med Chem. 2023 Jul 20;66(15):10432–57. doi: 10.1021/acs.jmedchem.3c00576 (PMC10424185; doi:10.1021/acs.jmedchem.3c00576)
Supplement: Supplementary file 1 — jm3c00576_si_001.pdf [file jm3c00576_si_001.pdf]

Supporting Information related to the article

C-2 Thiophenyl tryptophan trimers inhibit cellular entry of SARS-CoV-2 through interaction with the viral spike (S) protein

Marta Gargantilla,[1,∞] Clara Francés,[2,∞] Anmol Adhav,[3,#] Alicia Forcada Nadal,[3,4,#] Belén Martínez-Gualda,[1] Olaia Martí-Marí,[1] María Luisa López-Redondo,[3] Roberto Melero,[5] Clara Marco-Marín,[3,4] Nadine Gougeard,[3,4] Carolina Espinosa,[3] Antonio Rubio-del-Campo,[3] Rafael Ruiz-Partida,[3] María del Pilar Hernández-Sierra,[3] Laura Villamayor-Belinchón,[3] Jerónimo Bravo,[3] José-Luis Llacer,[3,4*] Alberto Marina,[3,4] Vicente Rubio,[3,4] Ana San-Félix,[1] Ron Geller[2*] and María-Jesús Pérez-Pérez[1*]

[1]*Instituto de Química Médica (IQM, CSIC) c/Juan de la Cierva 3, 28006 Madrid, Spain*

[2]*Institute for Integrative Systems Biology (I2SysBio), UV-CSIC, c/ Catedrático Agustin Escardino, 9, 46980 Paterna, Valencia, Spain*

[3]*Instituto de Biomedicina de Valencia (IBV, CSIC), c/ Jaime Roig 11, 46010 Valencia, Spain*

[4] Group 739, *Centro de Investigación Biomédica en Red en Enfermedades Raras (CIBERER-ISCIII)*

[5] *Centro Nacional de Biotecnología (CNB, CSIC), c/ Darwin 3, 28049, Madrid, Spain*

TABLE OF CONTENTS:

1. Synthesis and spectroscopic data of disulfides **20a-c** S2
2. Antiviral dose-response profiles measured using the VSV-S assay in both Vero E6 and A549-Ace2-TMPRSS2. Figure S1 S3
3. Correlation between the IC_{50} values for each compound observed in Vero E6 and A549-ACE2-TMPRSS2 obtained with the VSV-S antiviral assay. Figure S2. S4
4. ^{1}H and ^{13}C NMR spectra of selected compounds S6
5. HPLC chromatograms of selected compounds S24
6. References S26

1. Synthesis and spectroscopic data of disulfides 20a-c

General procedure for the synthesis of disulfides (General procedure D). To a solution containing the corresponding benzenesulfonyl chloride (1.0 mmol) in anhydrous DMF (3 mL), a solution of TBAI (3.0 mmol) in anhydrous DMF (3 mL) was added dropwise. The resulting solution was stirred at rt for 24 hours. Then, it was diluted with DCM (20 mL) and quenched with an aqueous solution of $Na_2S_2O_3$ (10 mL). The organic layer was washed with a saturated solution of $NaHCO_3$ (10 mL), dried over Na_2SO_4, filtered and evaporated to dryness and the residue was purified by flash chromatography.

4-Fluorophenyldisulfide (20a). Following the general procedure D, 4-cyanobenzenesulfonyl chloride (1.5 g, 7.71 mmol) and TBAI (8.54 g, 23.12 mmol) in anhydrous DMF (45 mL) reacted for 24 hours. After workup, the crude product was subjected to column chromatography (DCM/hexane, 1:2) to yield 213 mg (22%) of **20a** as yellow oil. ^{1}H NMR (400 MHz, DMSO-*d*$_6$) δ: 7.26 (m, 4H, Ar), 7.56 (m, 4H, Ar). ^{1}H NMR data are similar to those previously described.[1]

4-Acetylphenyldisulfide (20b). Following the general procedure D, 4-acetylbenzenesulfonyl chloride (1.0 g, 4.58 mmol) and TBAI (5.06 g, 13.92 mmol) in anhydrous DMF (26 mL) reacted for 24 hours. After workup, the crude product was subjected to column chromatography (DCM/hexane, 1:1) to yield 381 mg (22%) of **20b** as orange oil. ^{1}H NMR (400 MHz, DMSO-*d*$_6$) δ: 2.54 (s, 6H, $COCH_3$), 7.66 (m, 4H, Ar), 7.96 (m, 4H, Ar). ^{1}H NMR data are similar to those previously described.[2]

bis[4-(Methylsulfonyl)phenyl)disulfide (20c). Following the general procedure D, 4-(methylsulfonyl)benzenesulfonyl chloride (1.5 g, 5.97 mmol) and TBAI (6.6 g, 17.90 mmol) in anhydrous DMF (35.8 mL) reacted for 24 hours. After workup, the crude product was subjected to column chromatography (DCM/ethyl acetate, 10:1) to yield 290 mg (26%) of **20c** as an amorphous white solid. ^{1}H NMR (400 MHz, DMSO-*d*$_6$) δ: 3.21 (s, 6H, $SO_2C\underline{H_3}$), 7.80 (m, 4H, Ar), 7.93 (m, 4H, Ar).

2. **Antiviral dose-response profiles measured using the VSV-S assay in both Vero E6 and A549-Ace2-TMPRSS2**

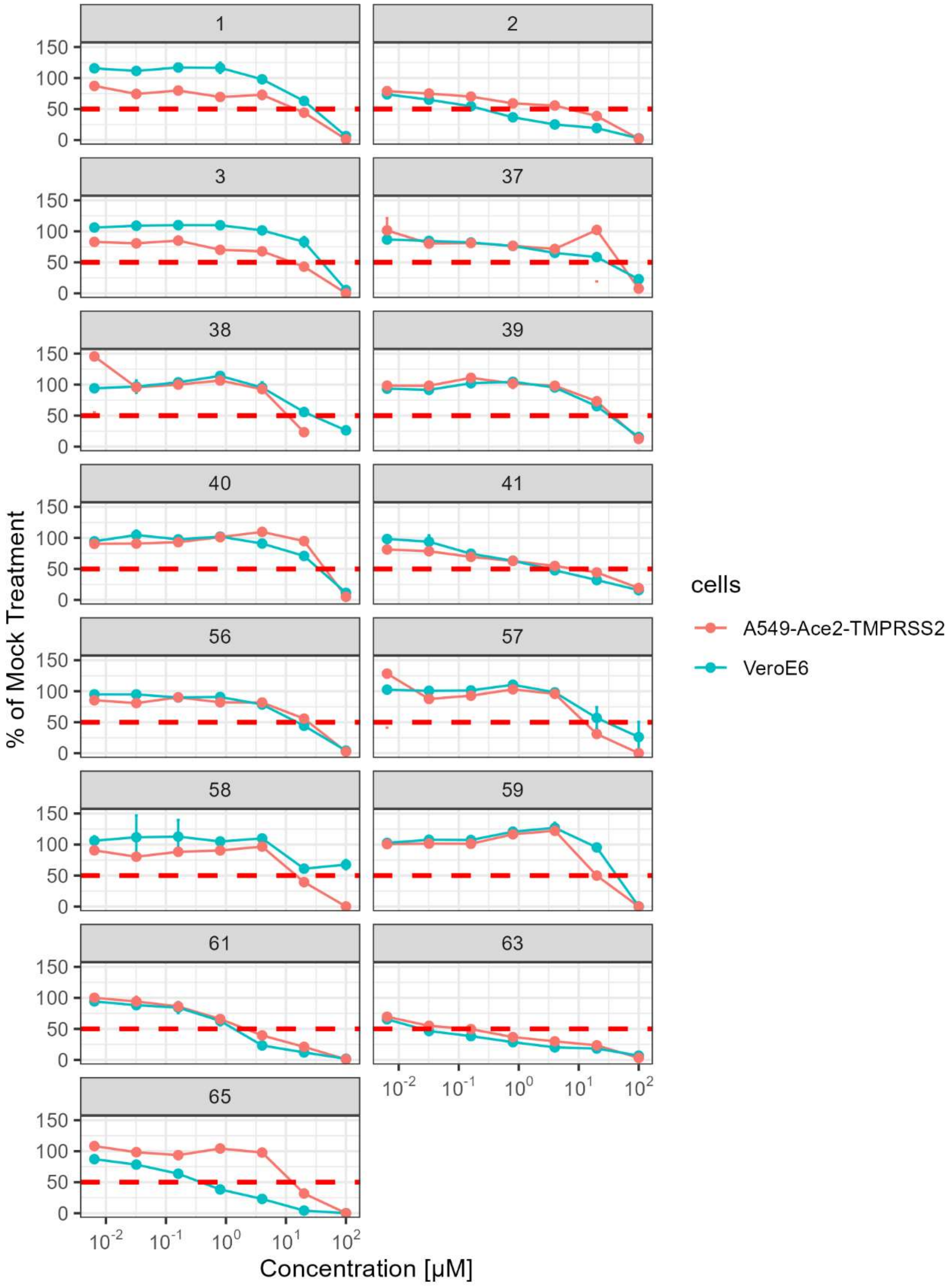

Figure S1. Antiviral dose-response profiles measured using the VSV-S assay in both Vero E6 and A549-Ace2-TMPRSS2. Data indicates the mean and SEM of at least 3 replicates in each cell line.

3. **Correlation between the IC_{50} values for each compound observed in Vero E6 and A549-ACE2-TMPRSS2 obtained with the VSV-S antiviral assay**

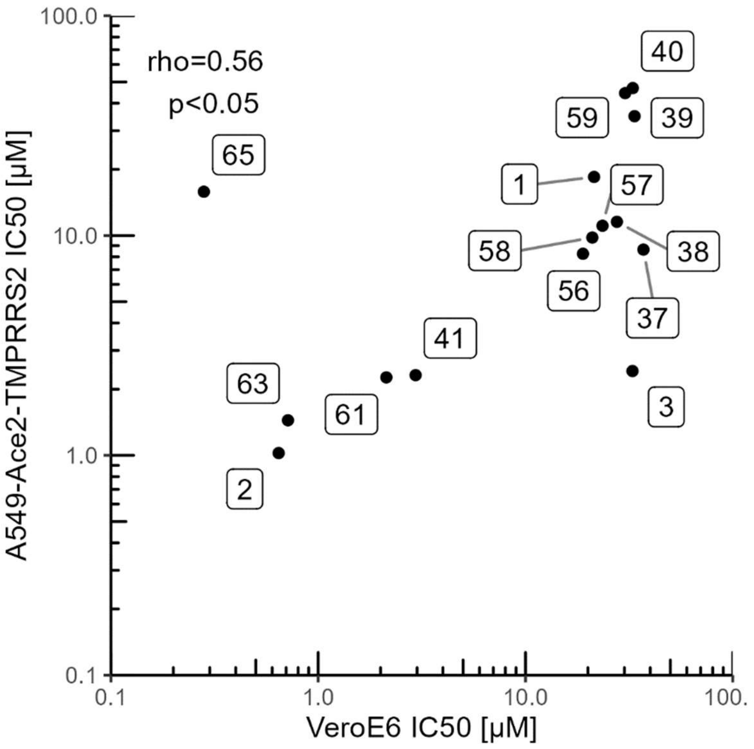

Figure S2. Correlation between the IC_{50} values for each compound observed in Vero E6 and A549-ACE2-TMPRSS2 obtained with the VSV-S antiviral assay. Data represents the mean of at least three independent replicates.

4. ^{1}H and ^{13}C NMR spectra of selected compounds

Tetramer 1

^{1}H NMR (400 MHz, DMSO-d_6)

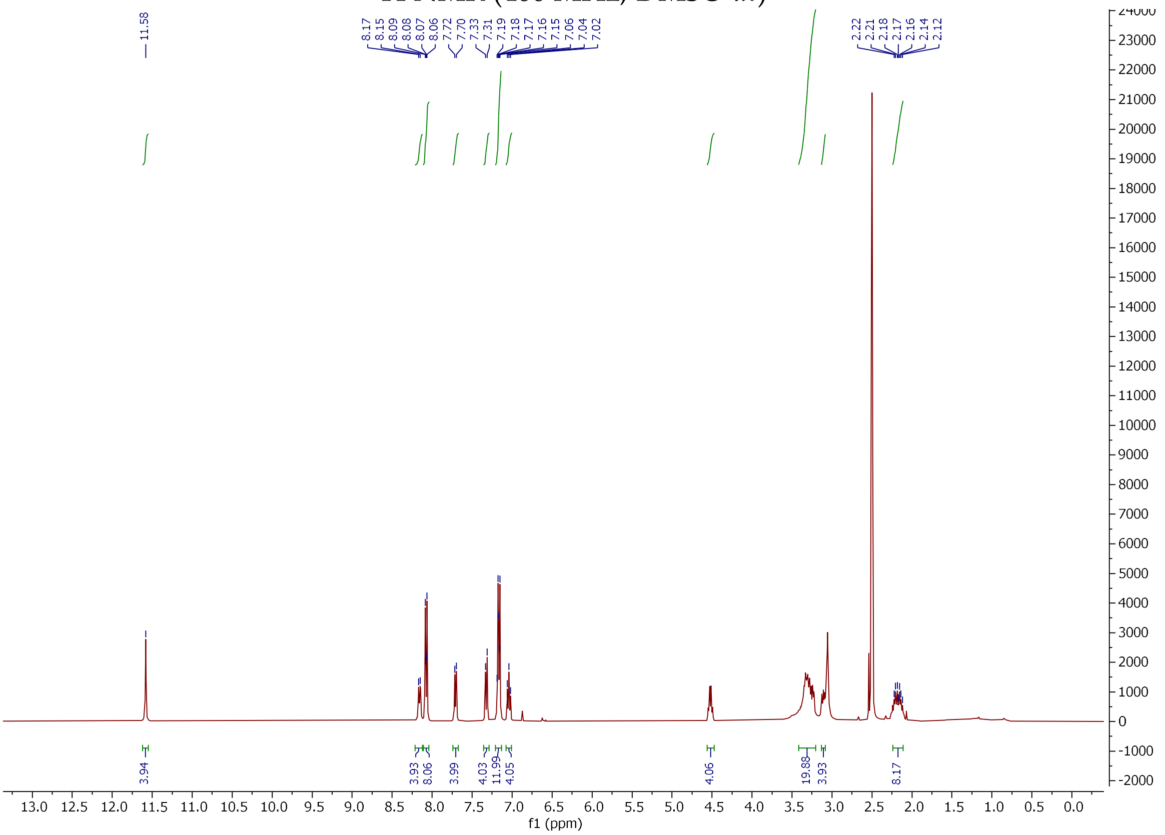

^{13}C NMR (101 MHz, DMSO-d_6)

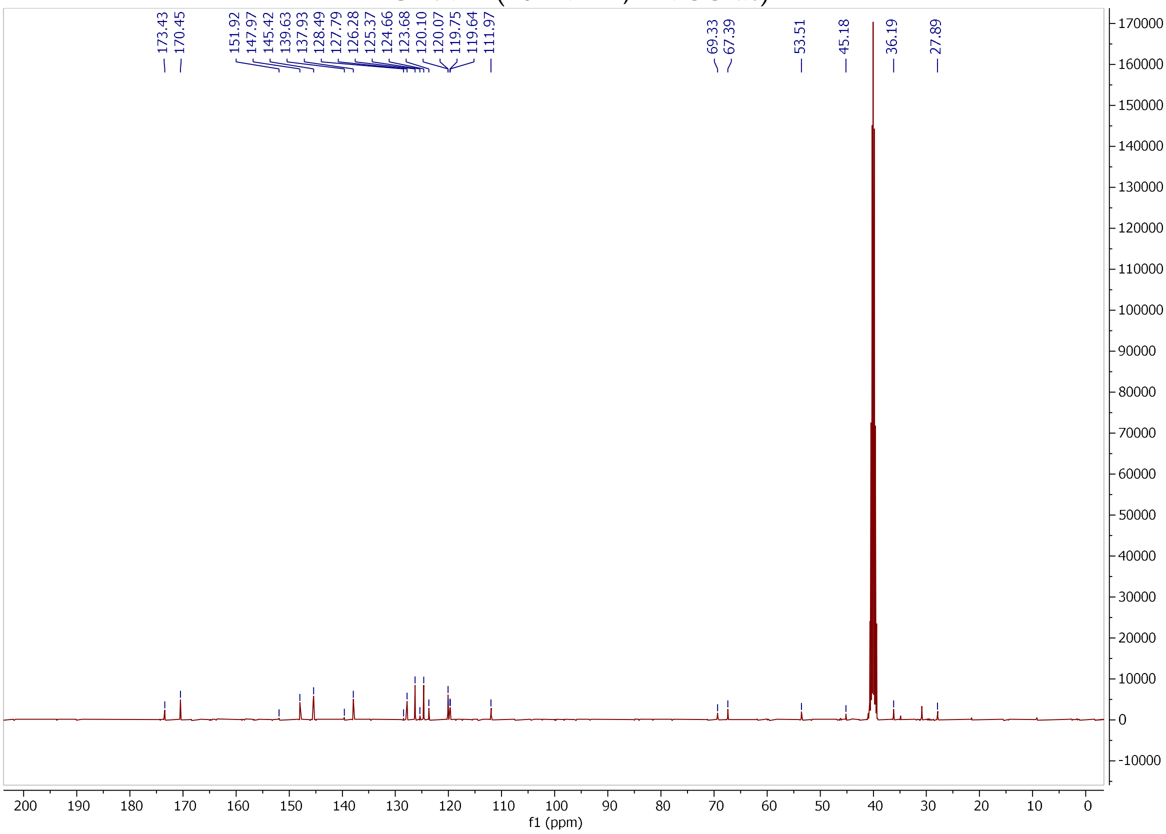

Trimer 2

^{1}H NMR (400 MHz, DMSO-d_6)

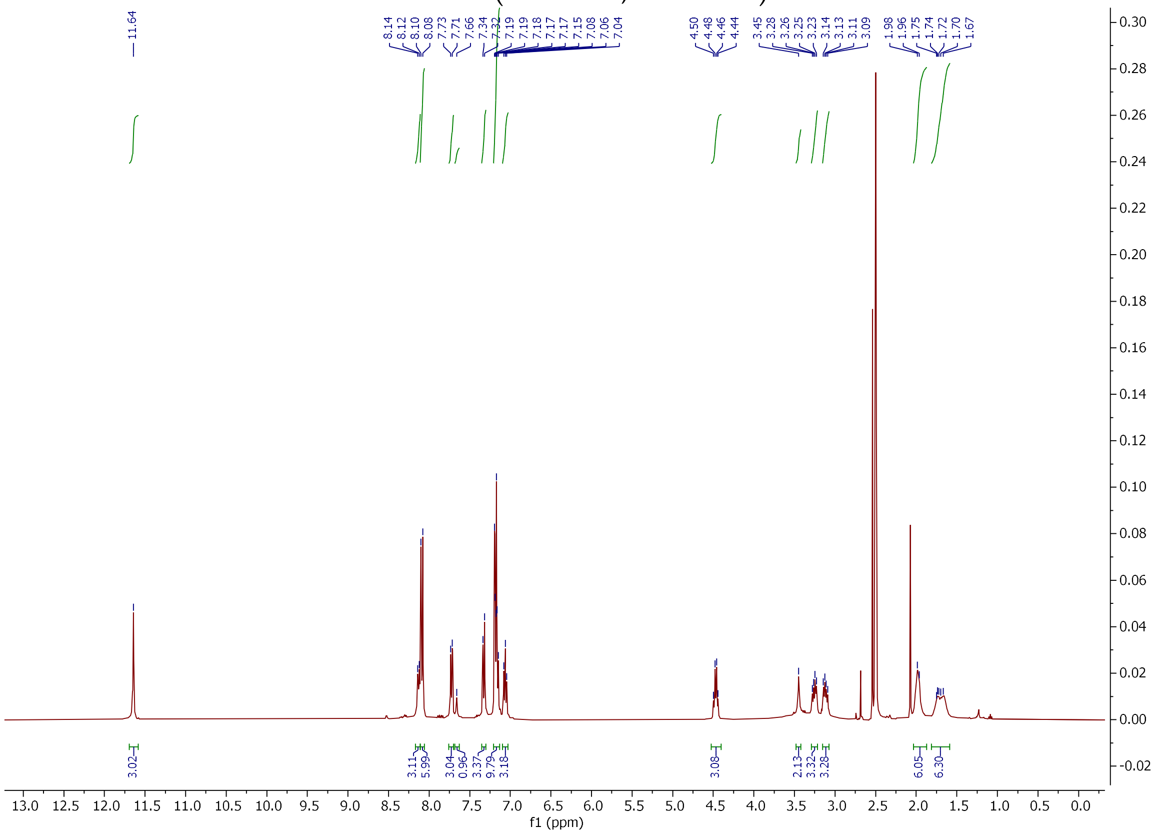

^{13}C NMR (101 MHz, DMSO-d_6)

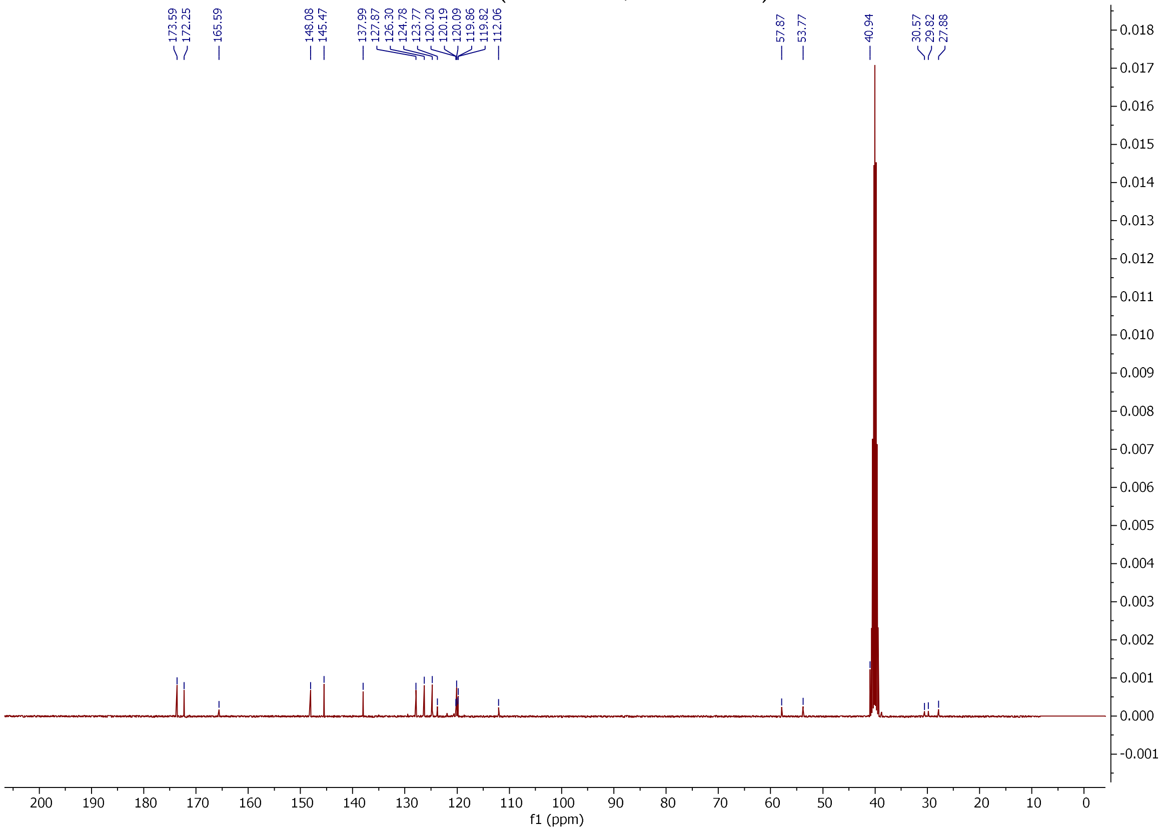

Trimer 3

^{1}H NMR (400 MHz, DMSO-d_6)

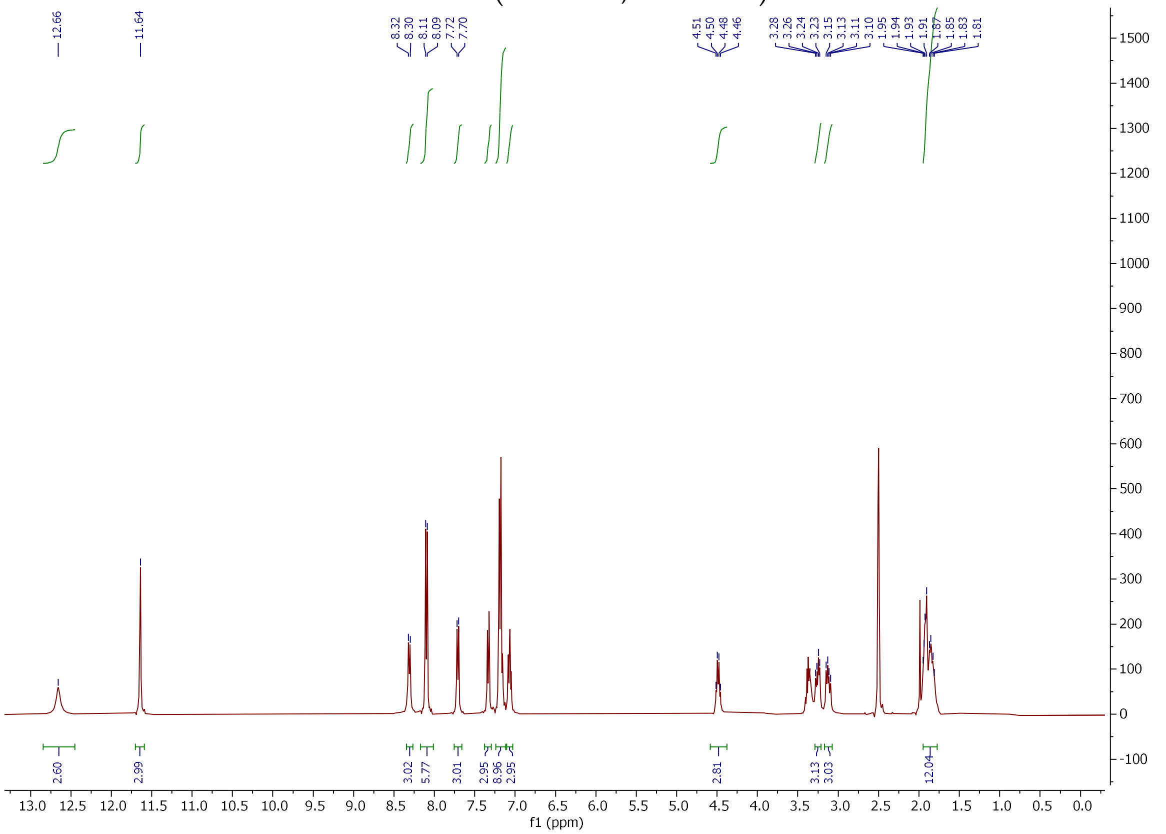

^{13}C NMR (101 MHz, DMSO-d_6)

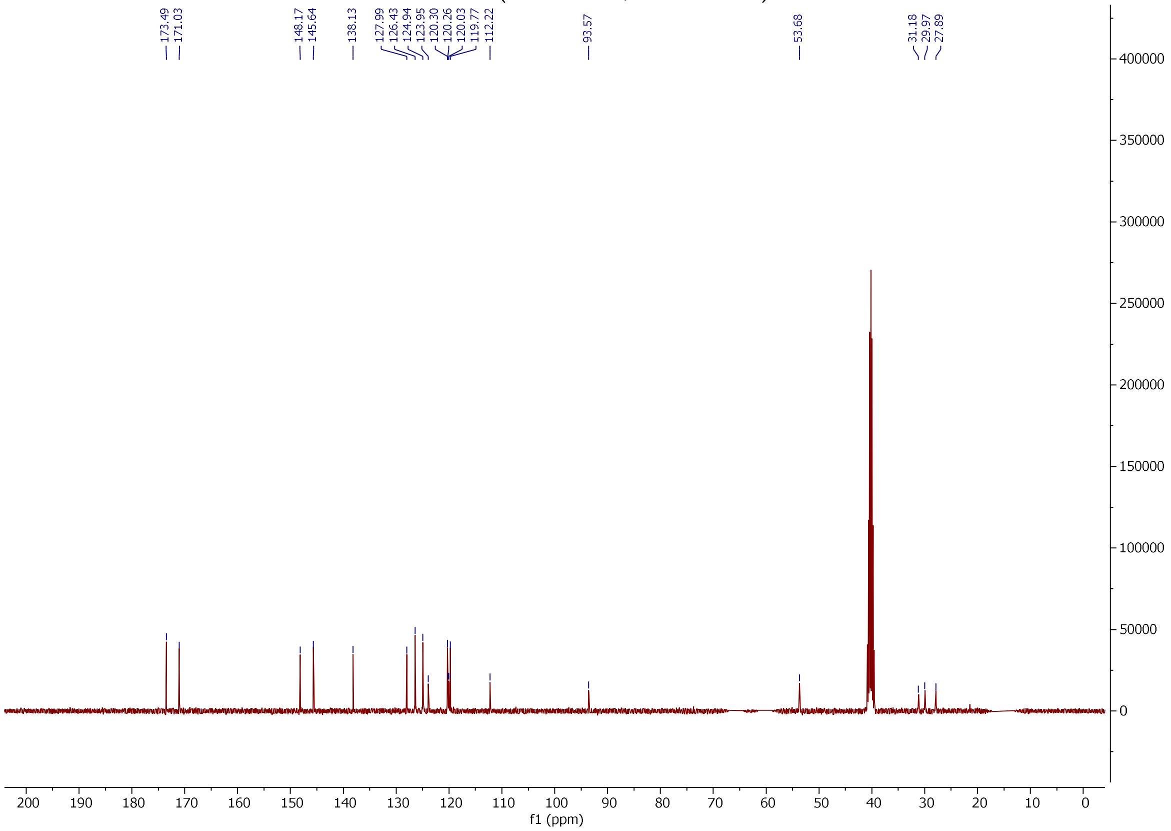

Trimer 36

^{1}H NMR (500 MHz, DMSO-d_6)

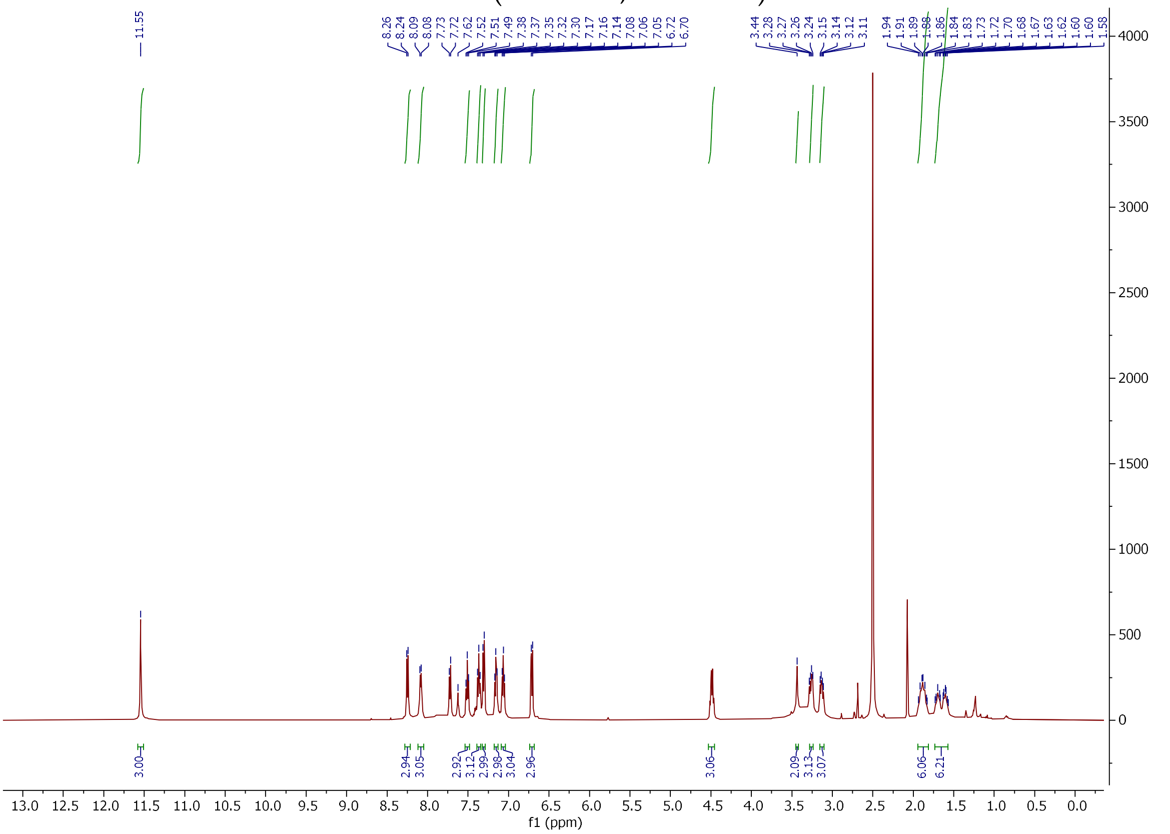

^{13}C NMR (126 MHz, DMSO-d_6)

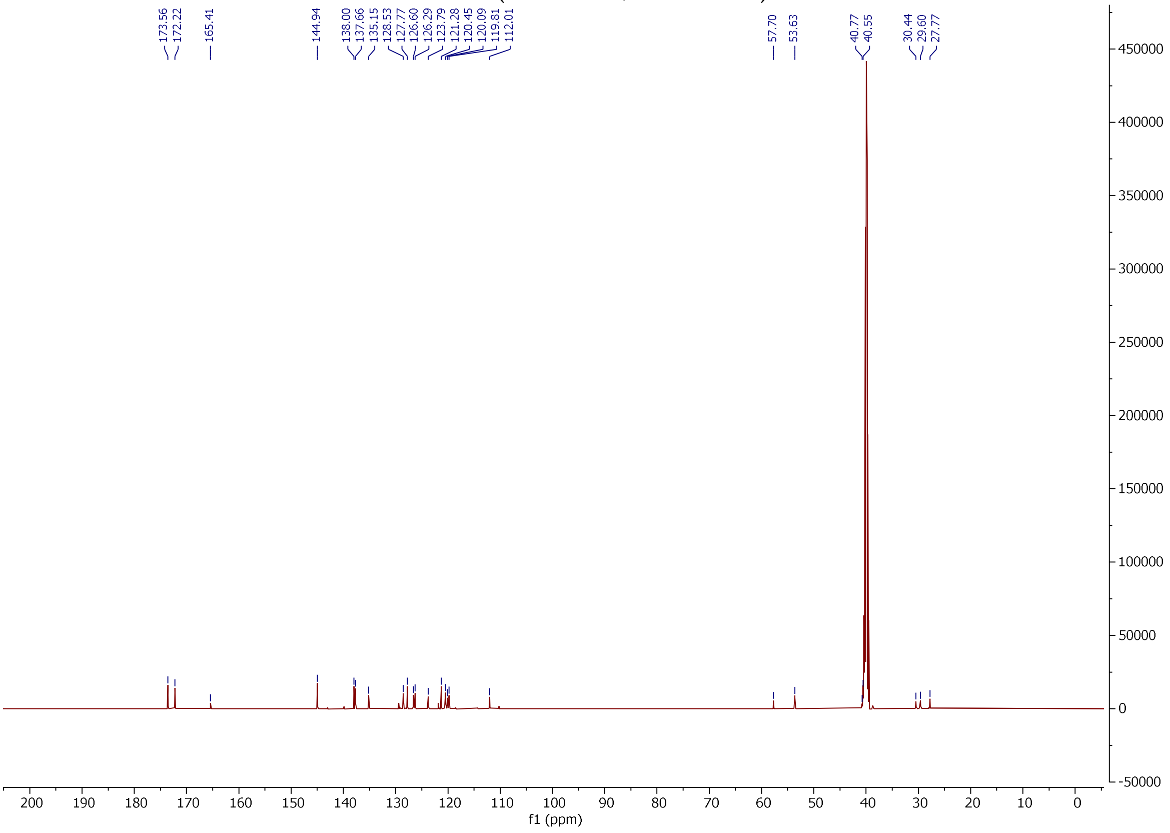

Trimer 37

¹H NMR (400 MHz, DMSO-d_6)

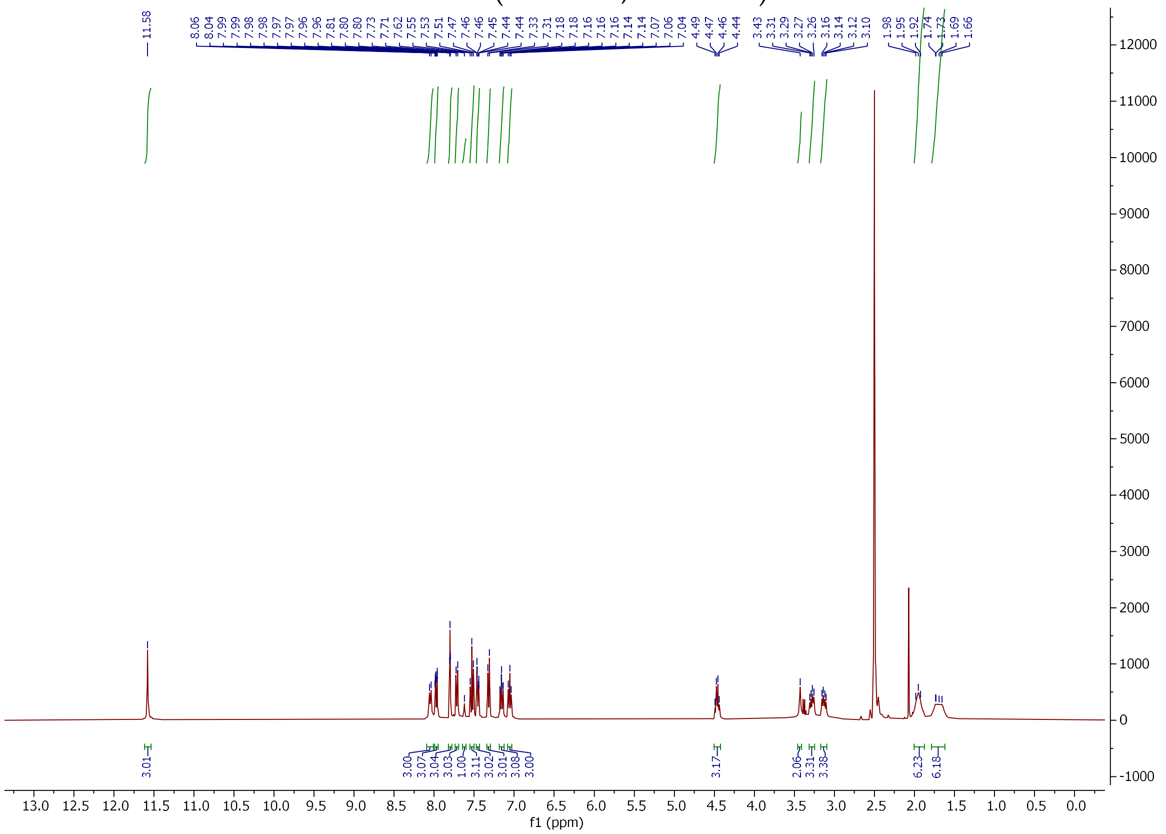

¹³C NMR (101 MHz, DMSO-d_6)

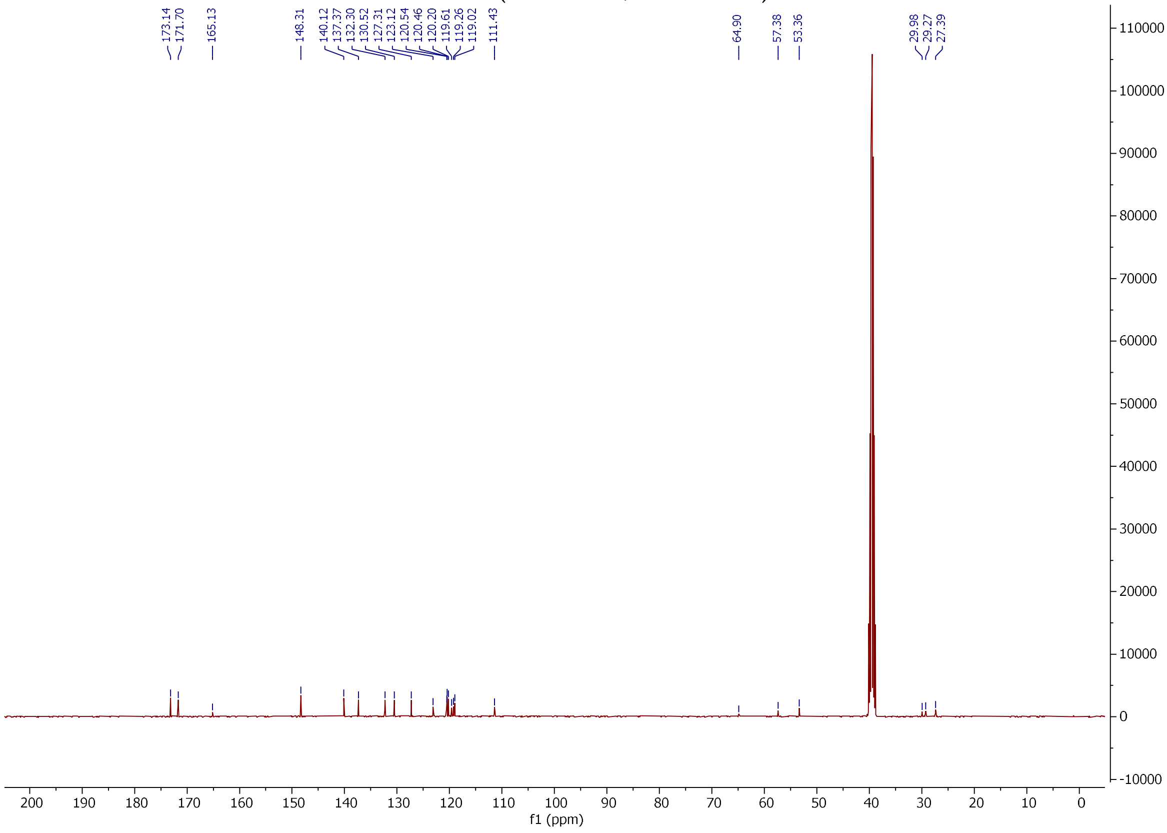

Trimer 38

^{1}H NMR (500 MHz, DMSO-d_6)

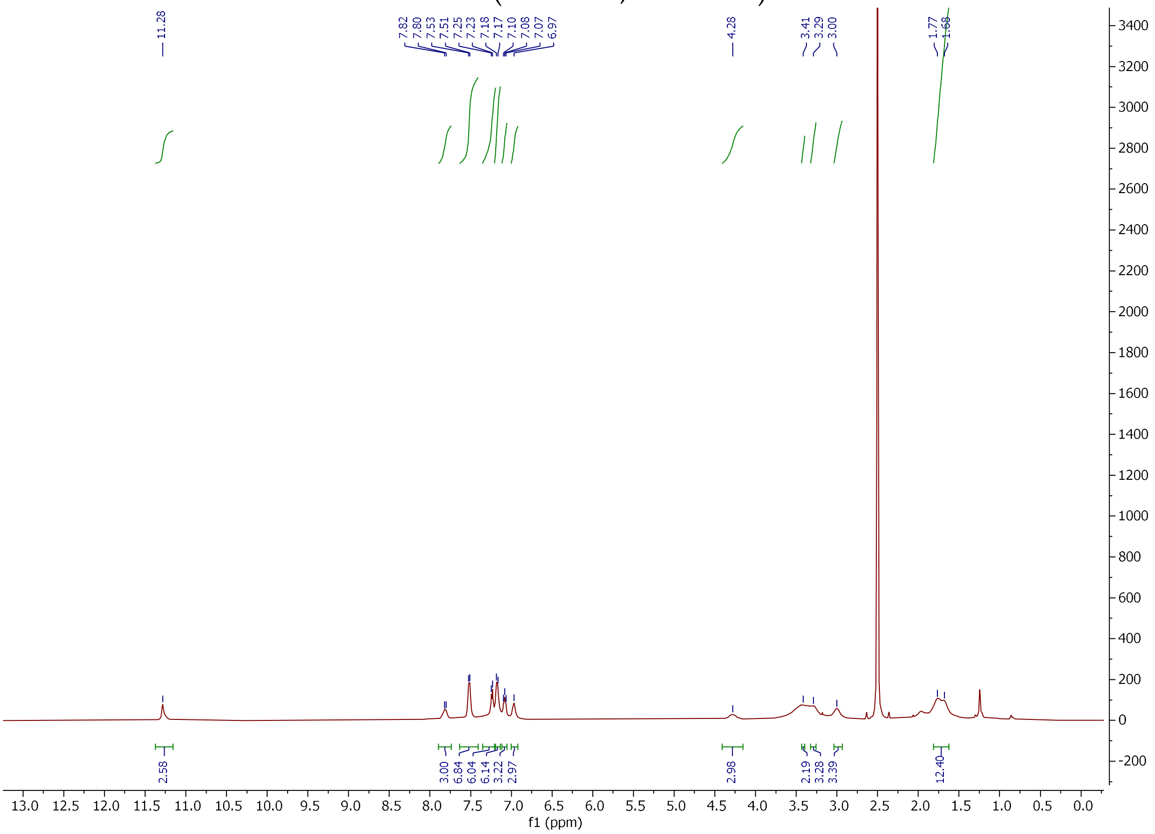

^{13}C NMR (126 MHz, DMSO-d_6)

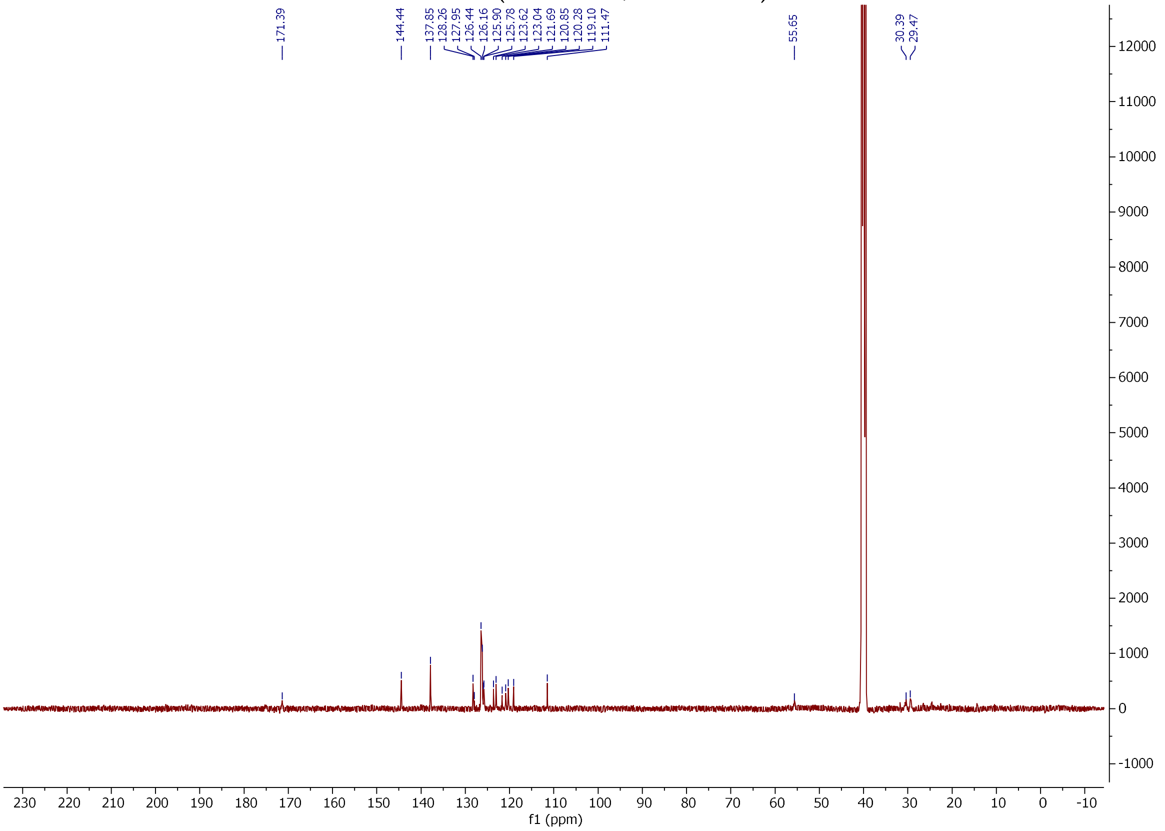

Trimer 39

^{1}H NMR (500 MHz, DMSO-d_6)

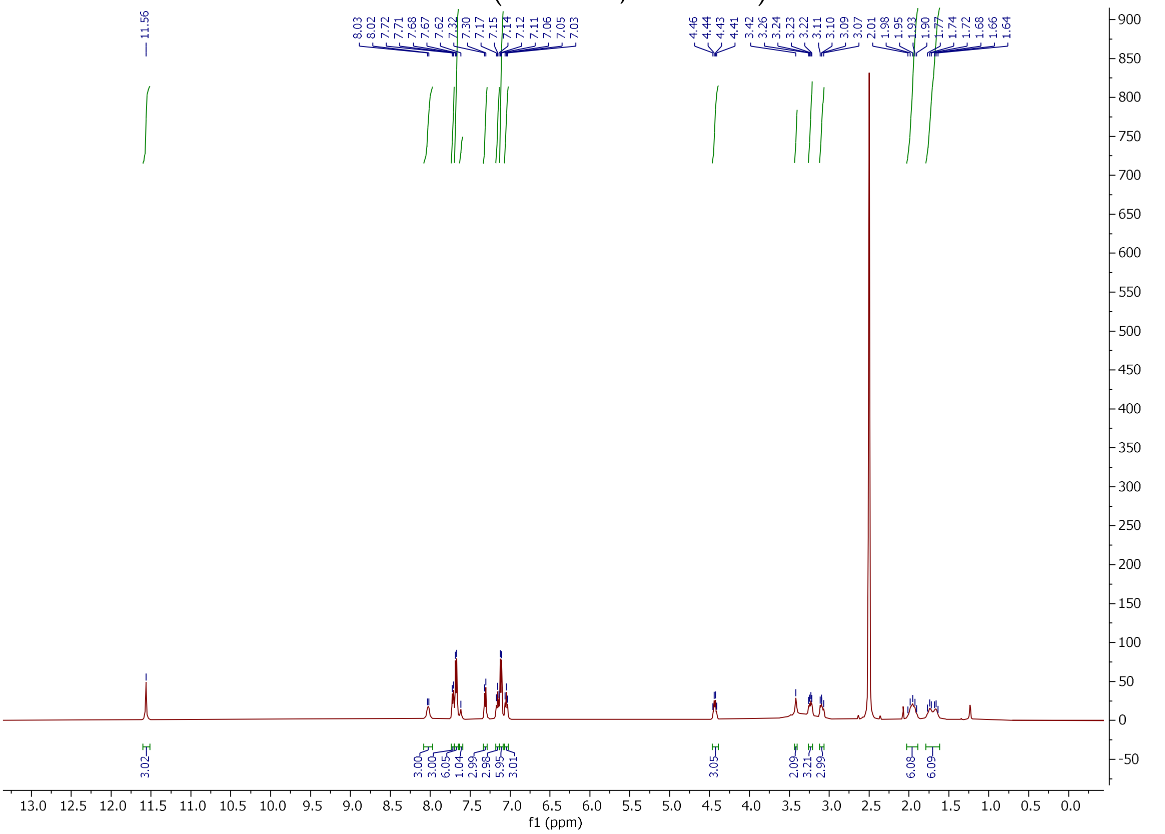

^{13}C NMR (126 MHz, DMSO-d_6)

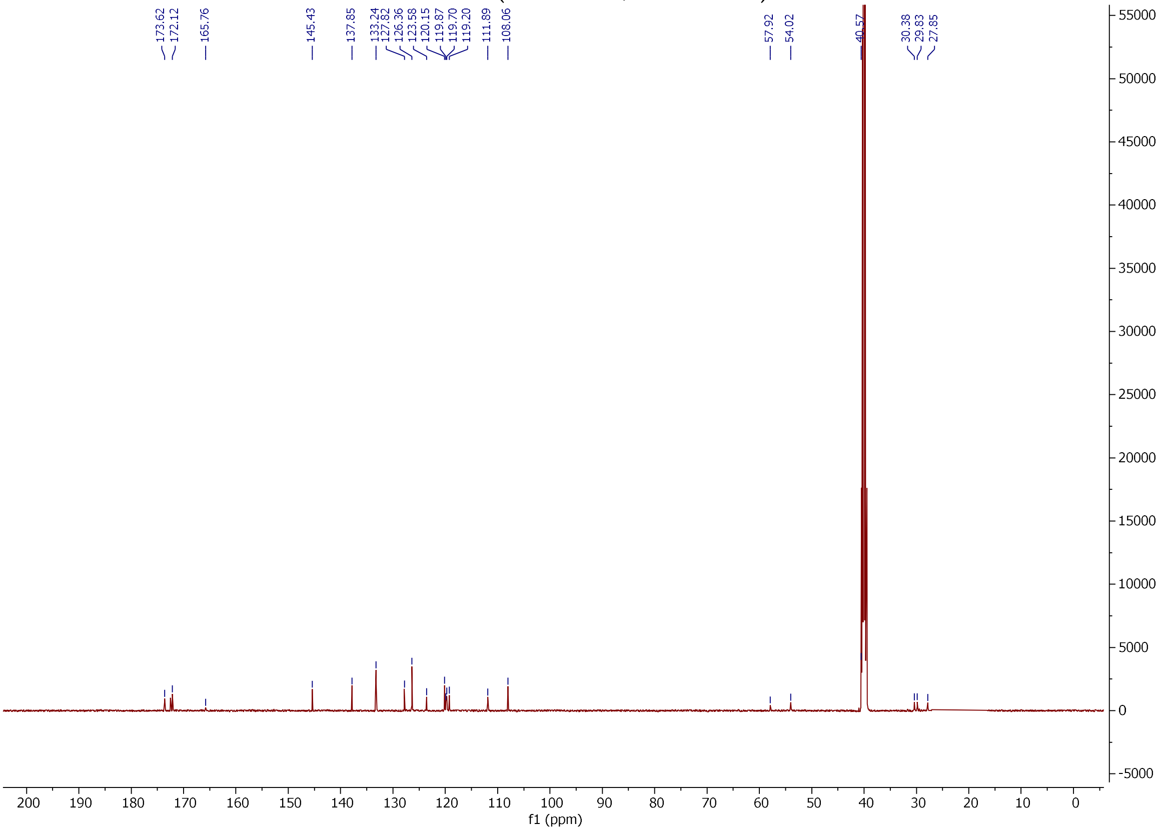

Trimer 40

^{1}H NMR (500 MHz, DMSO-d_6)

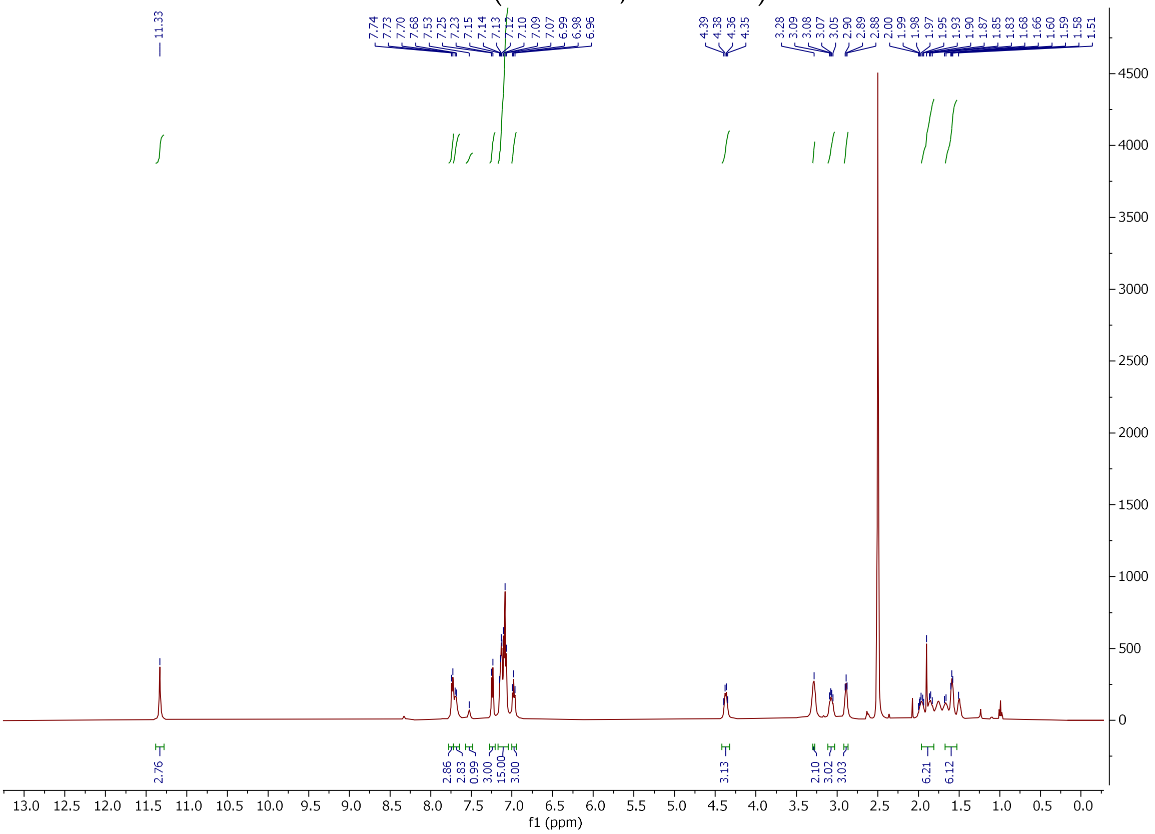

^{13}C NMR (126 MHz, DMSO-d_6)

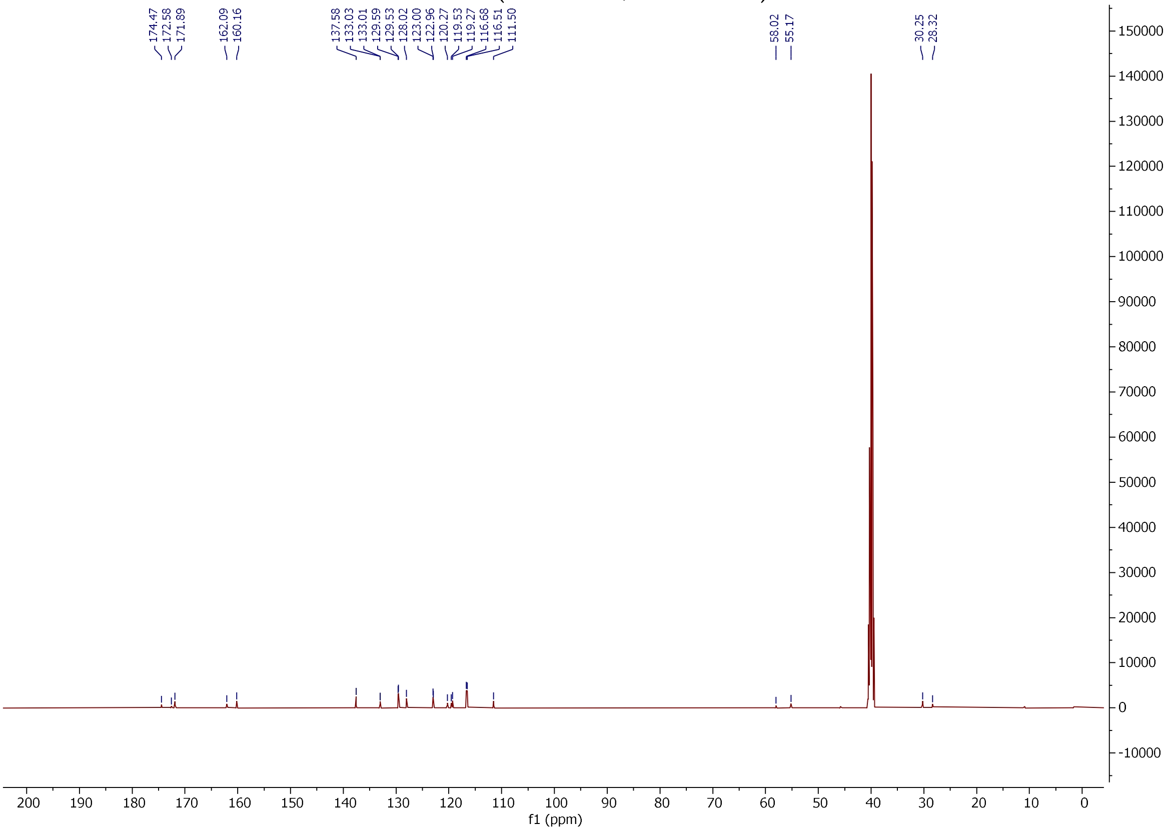

Trimer 41

^1H NMR (400 MHz, DMSO-d_6)

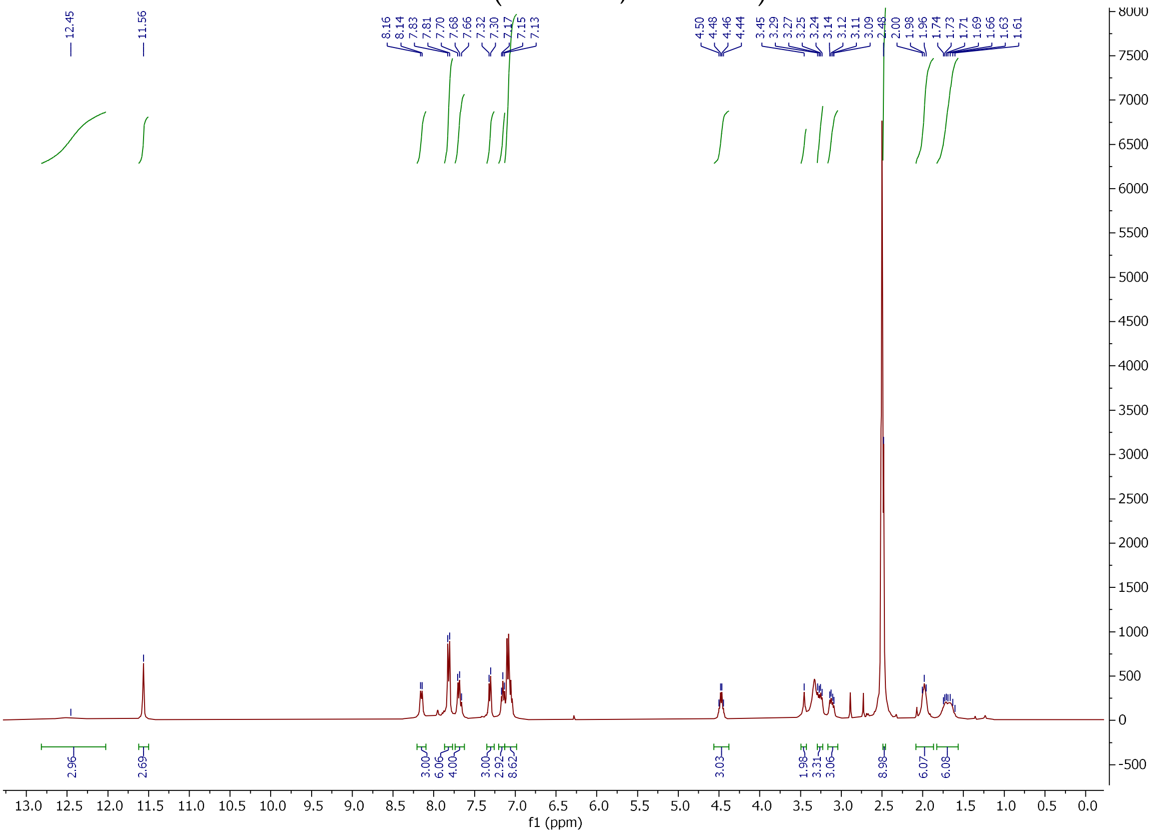

^{13}C NMR (101 MHz, DMSO-d_6)

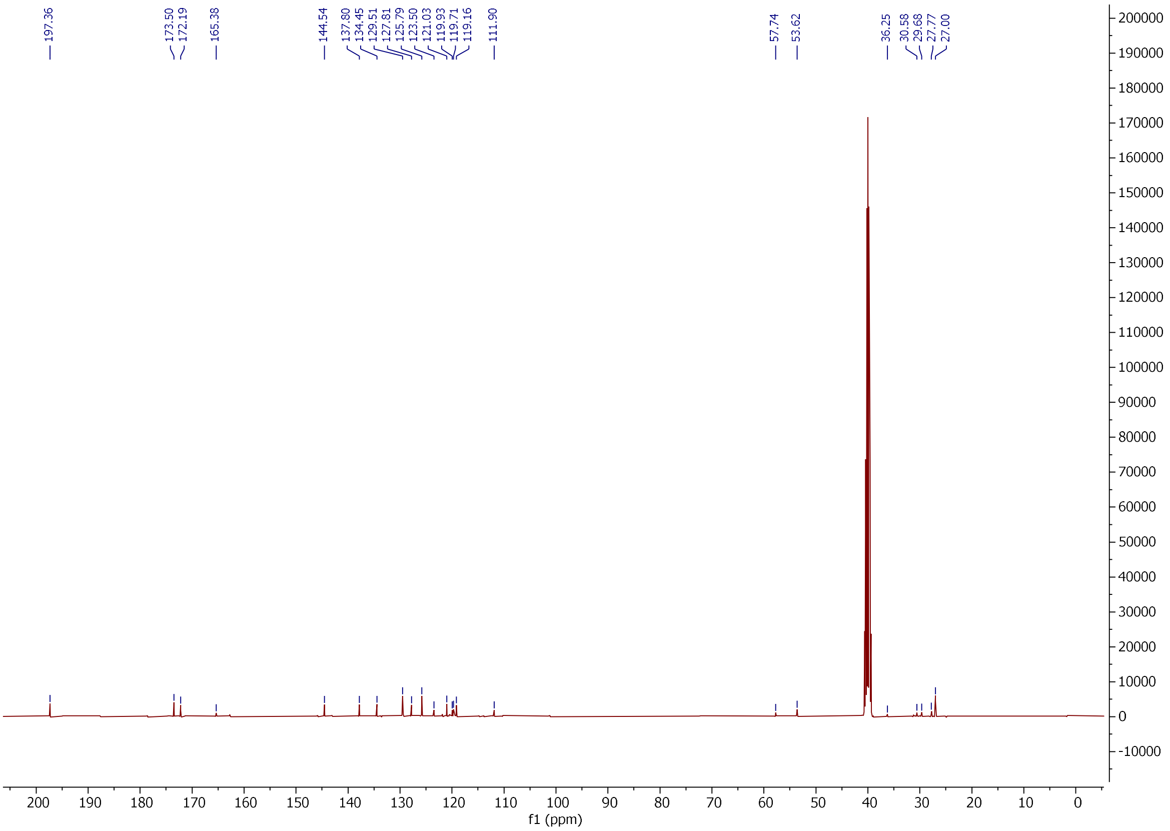

Trimer 42

¹H NMR (400 MHz, DMSO-*d*₆)

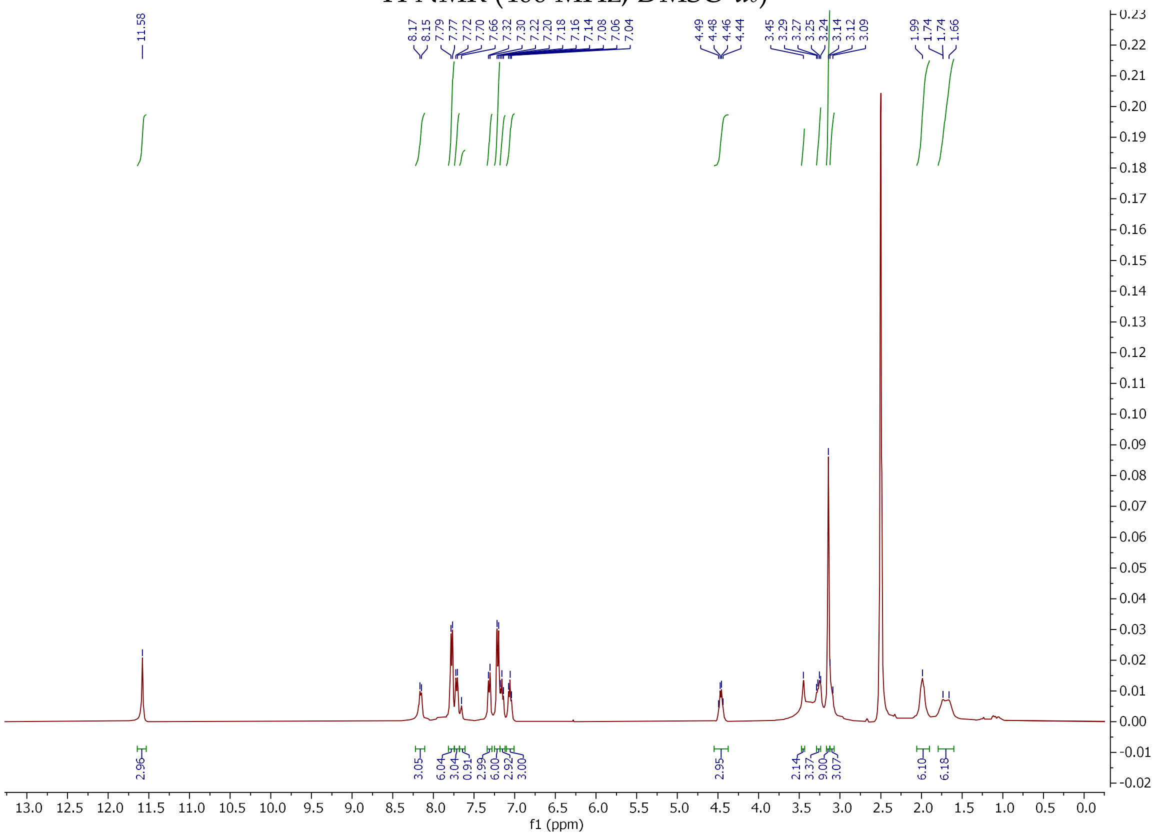

¹³C NMR (101 MHz, DMSO-*d*₆)

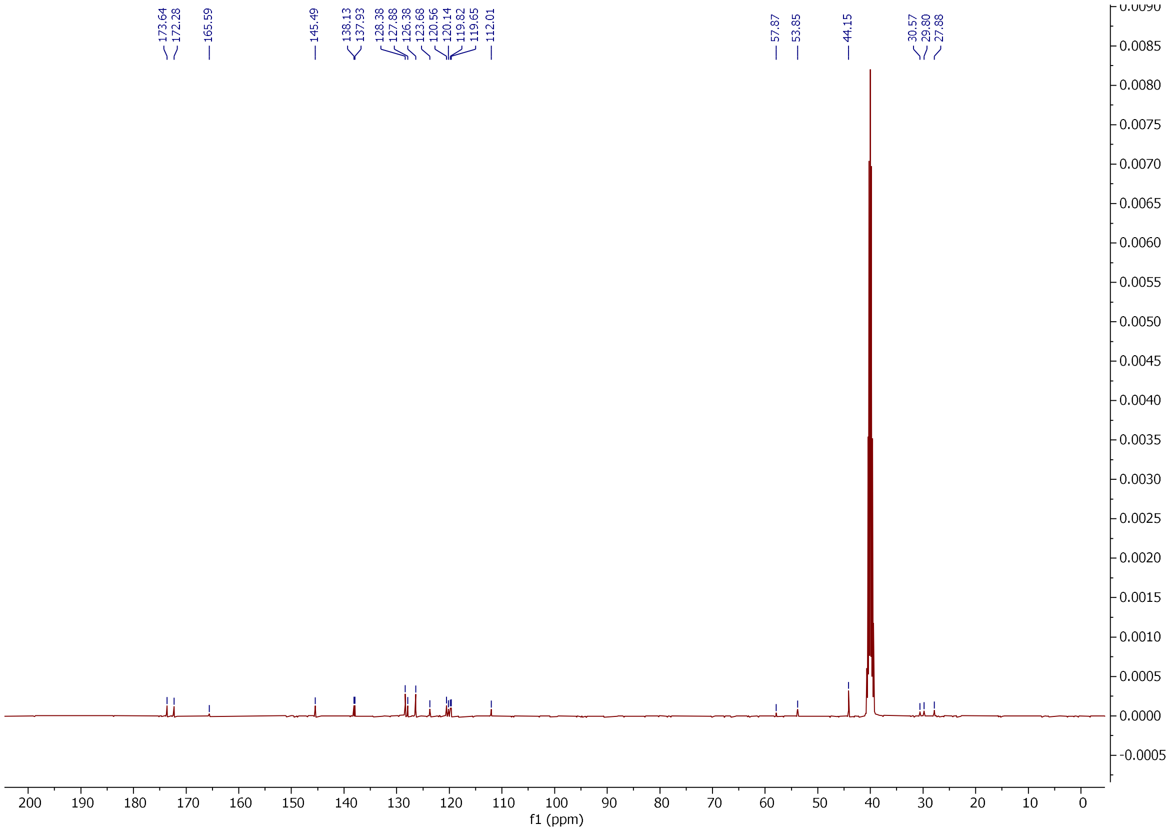

Trimer 46

^{1}H NMR (400 MHz, DMSO-d_6)

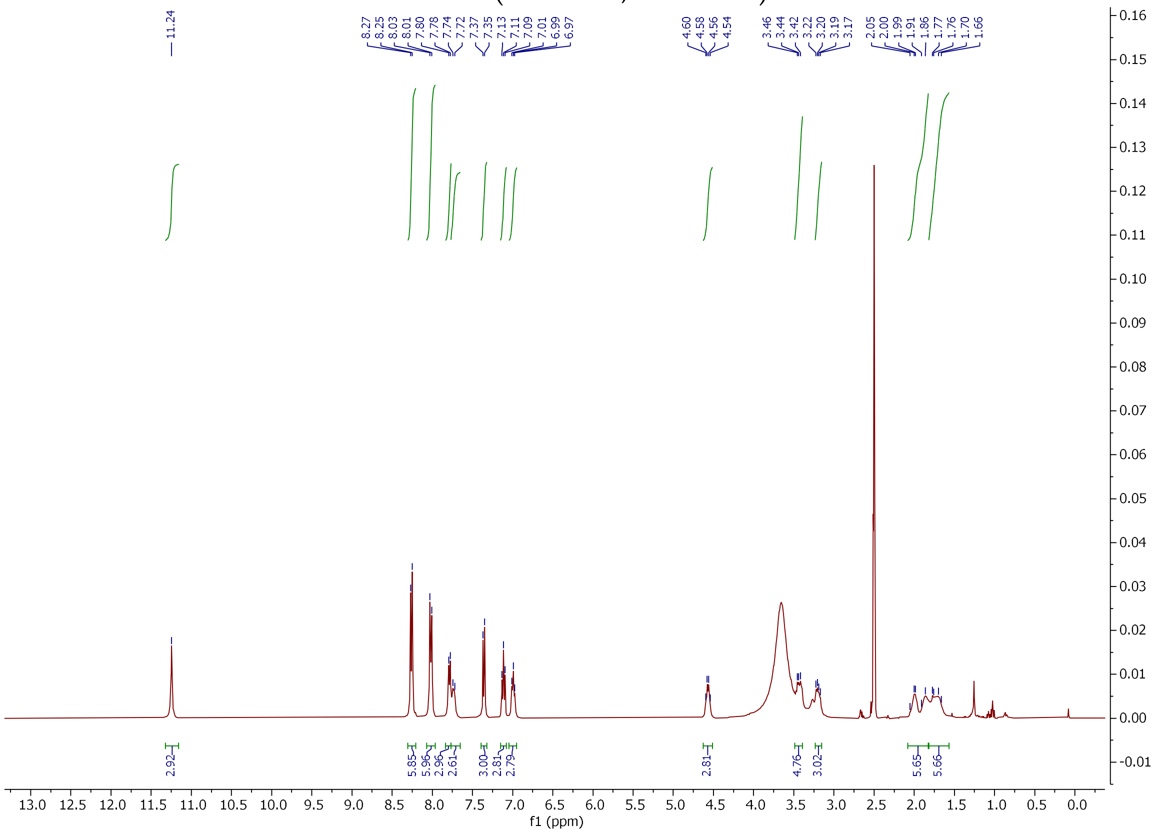

^{13}C NMR (101 MHz, DMSO-d_6)

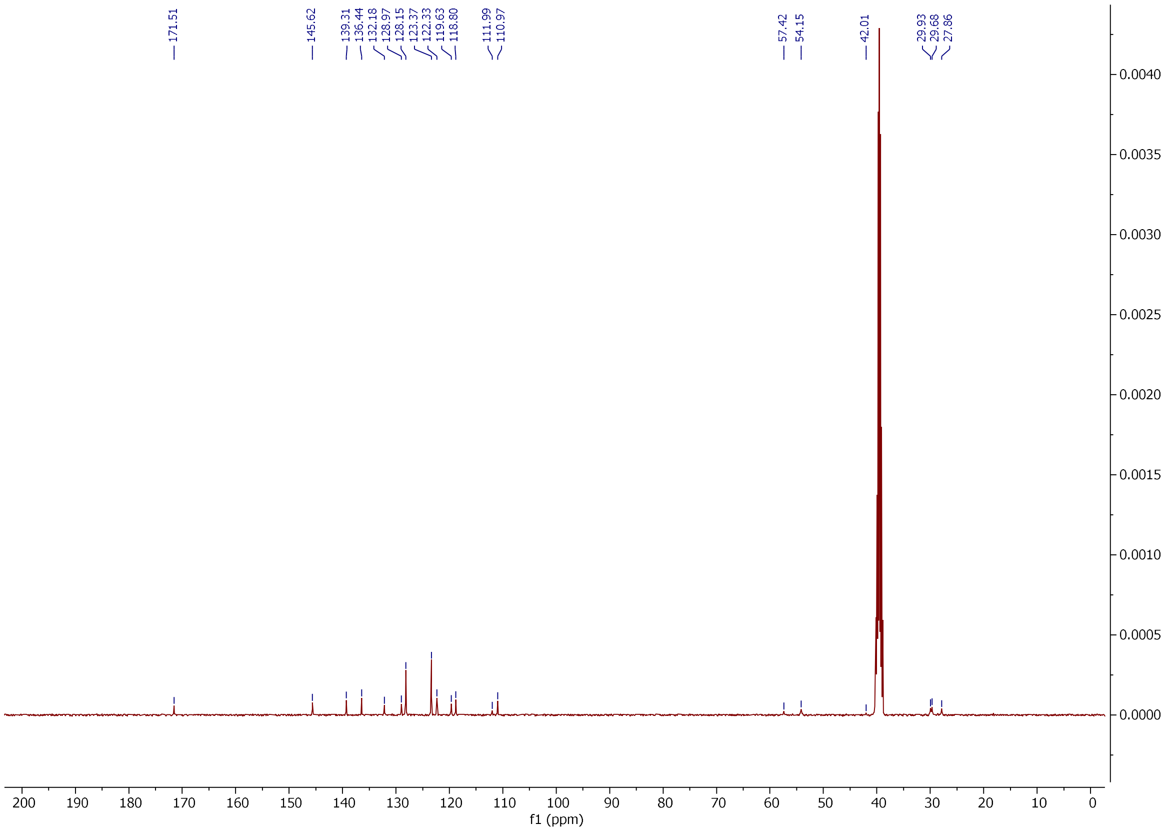

Trimer 50

^{1}H NMR (500 MHz, DMSO-d_6)

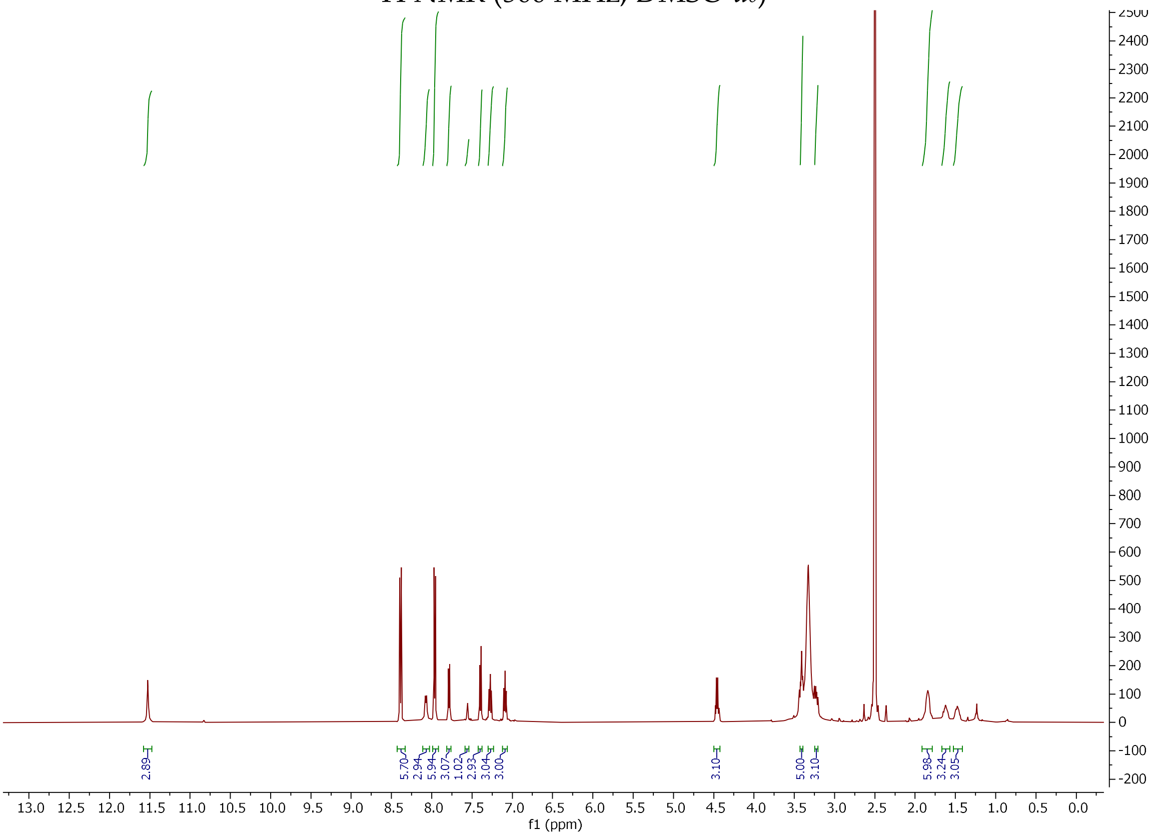

^{13}C NMR (126 MHz, DMSO-d_6)

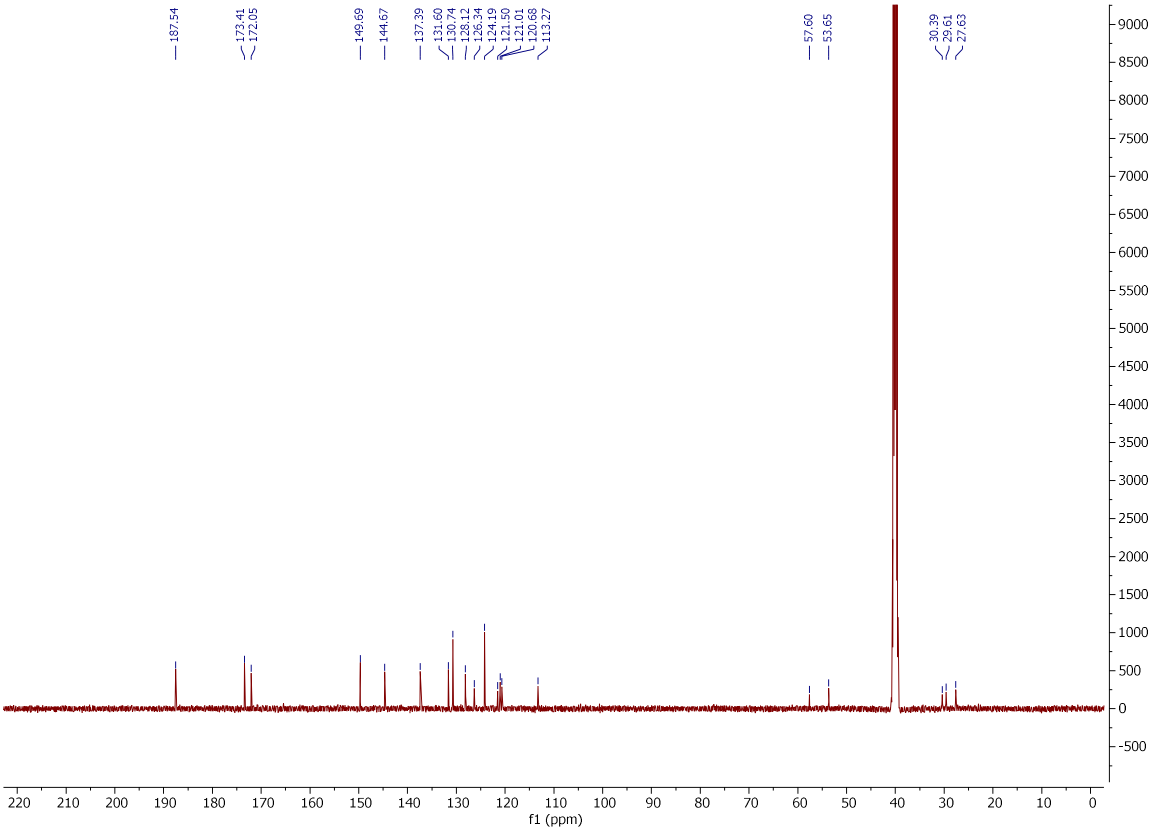

Trimer 56

^{1}H NMR (500 MHz, DMSO-d_6)

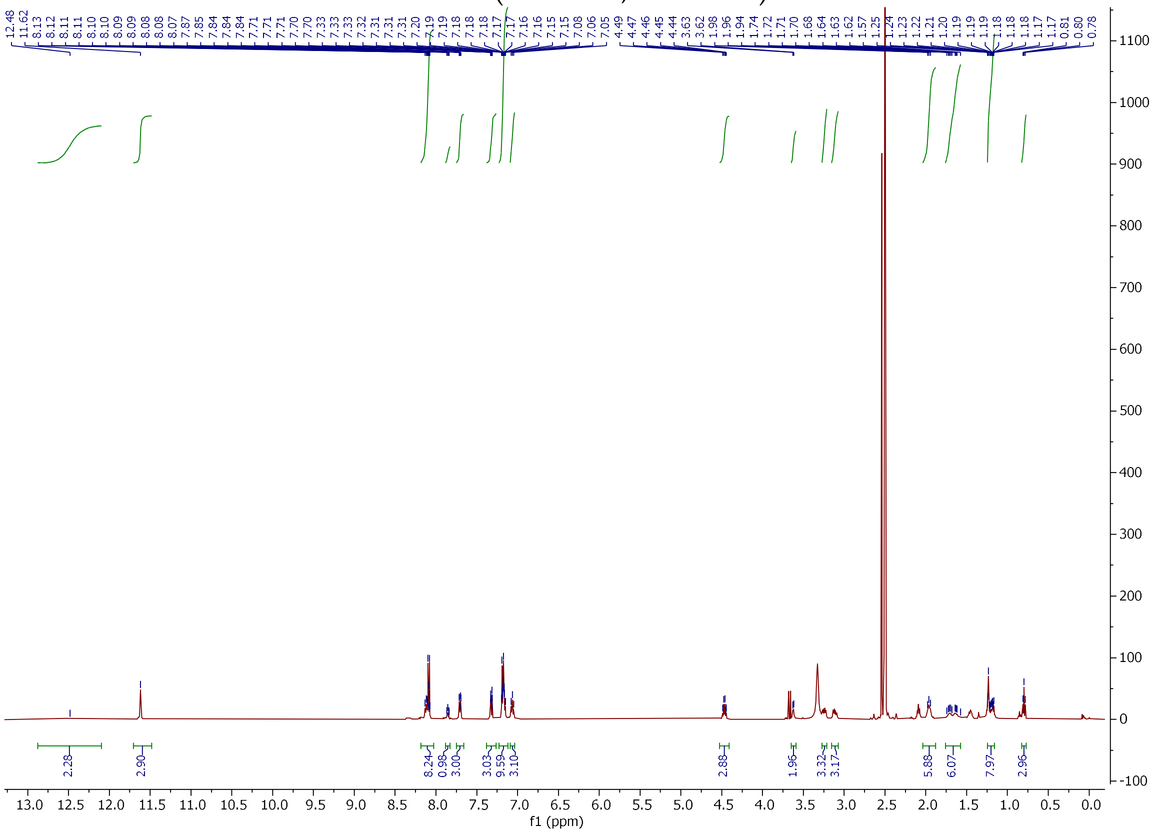

^{13}C NMR (126 MHz, DMSO-d_6)

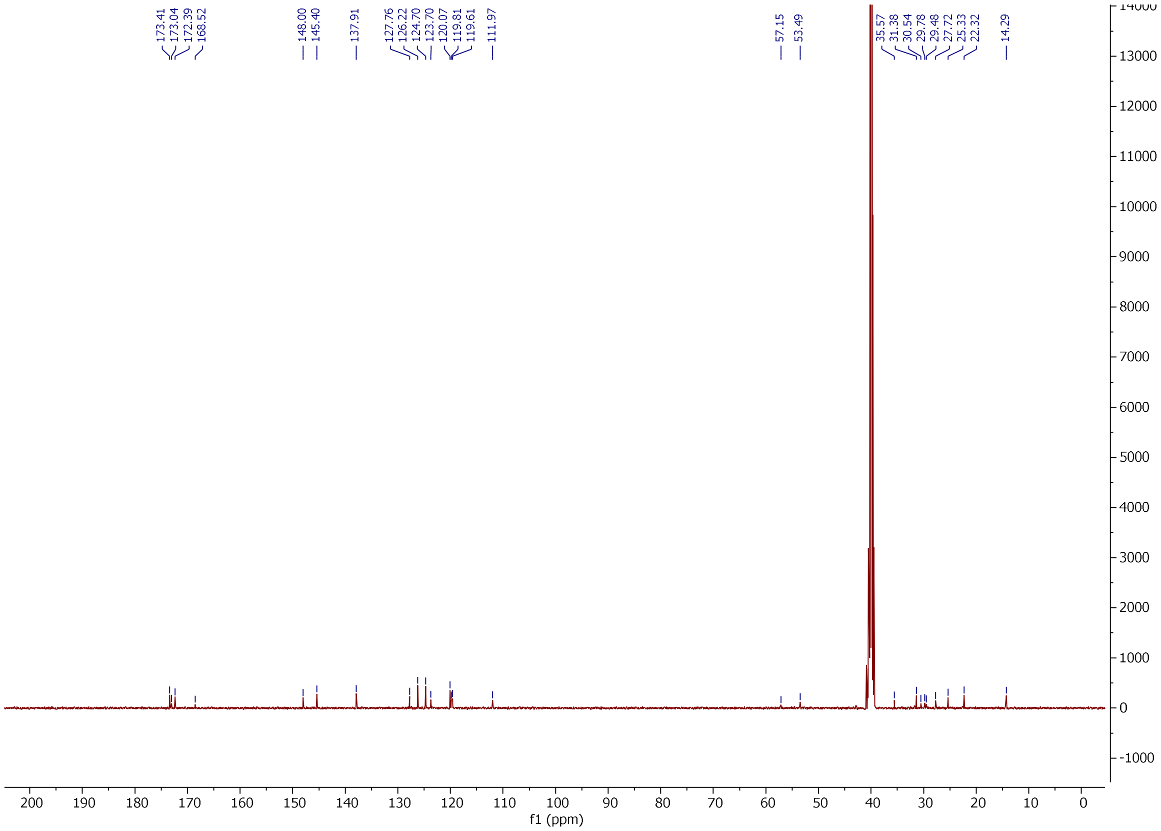

Trimer 57

^{1}H NMR (400 MHz, DMSO-d_6)

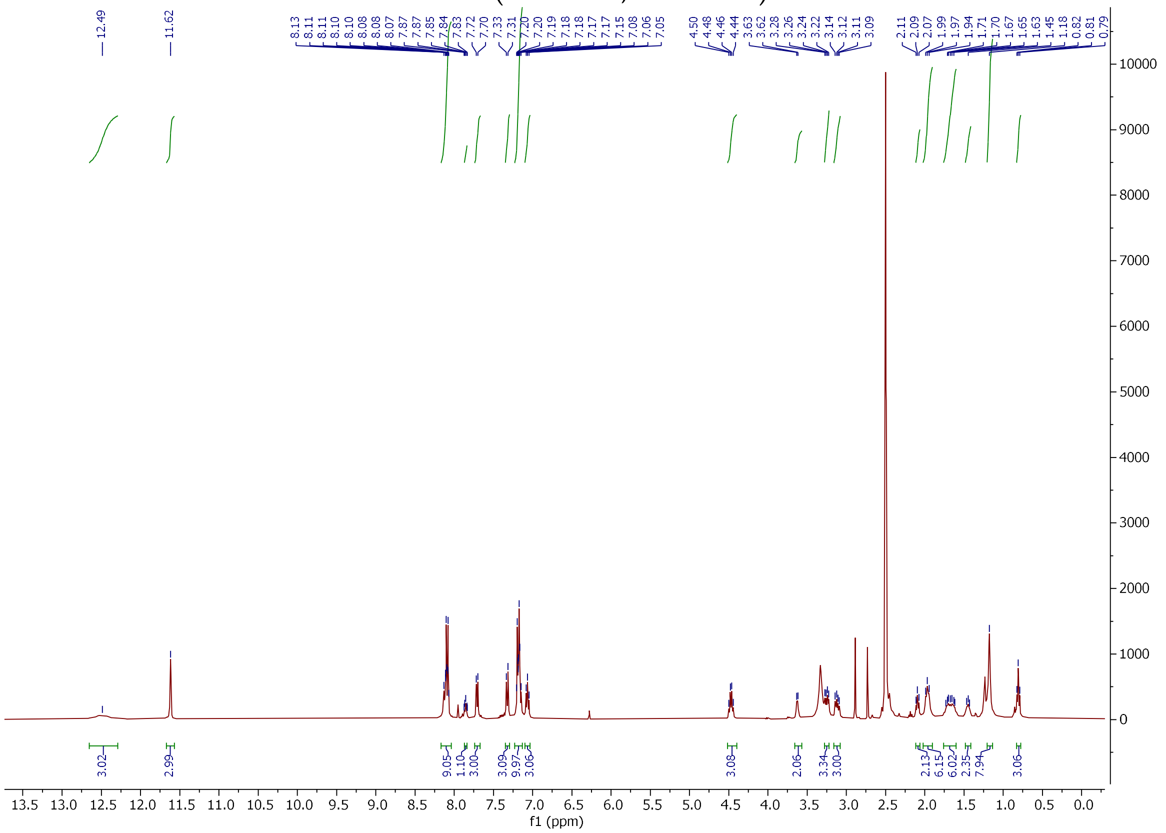

^{13}C NMR (101 MHz, DMSO-d_6)

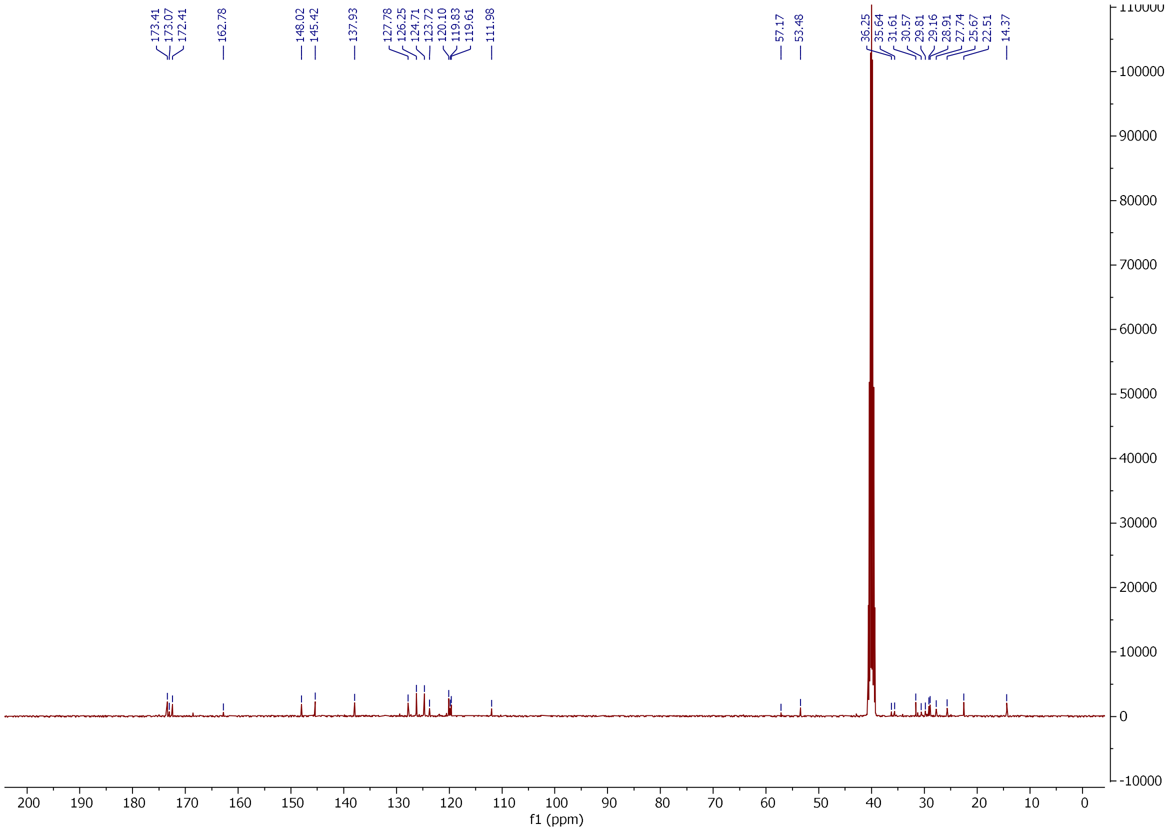

Trimer 58

^{1}H NMR (500 MHz, DMSO-d_6)

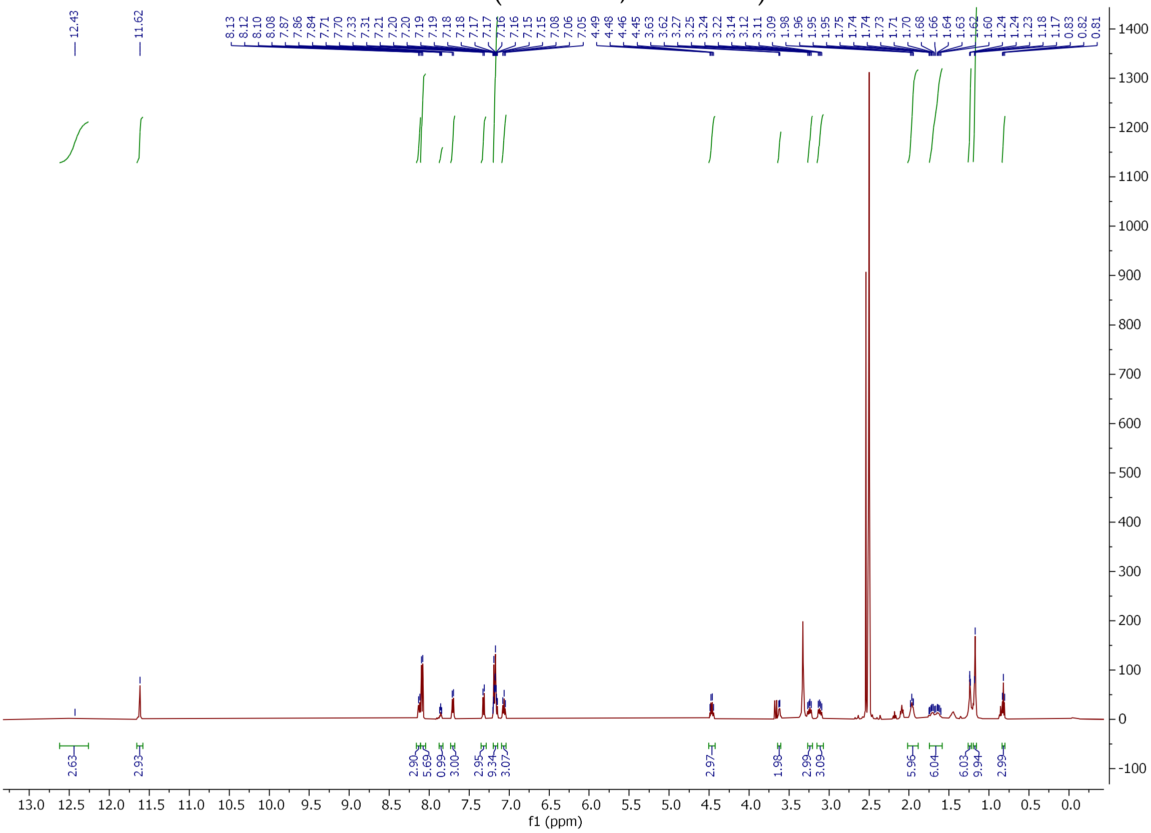

^{13}C NMR (126 MHz, DMSO-d_6)

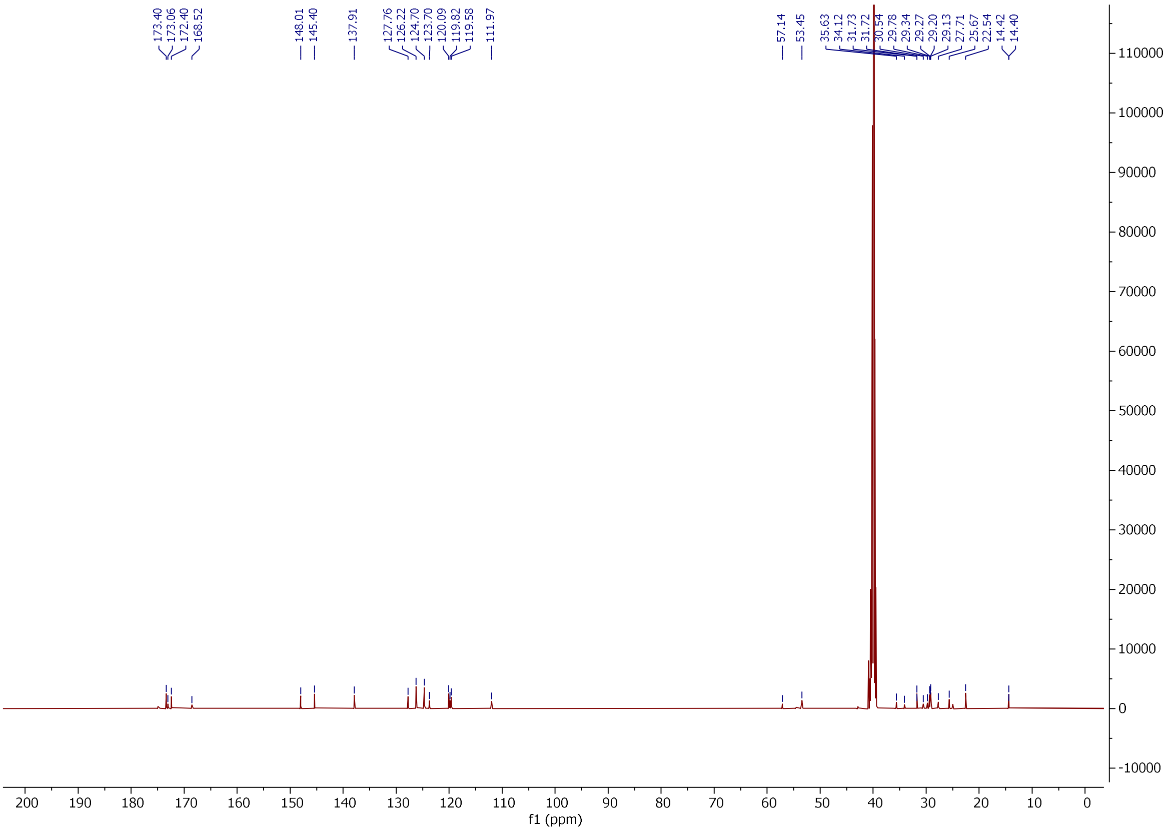

Trimer 59

^{1}H NMR (400 MHz, DMSO-d_6)

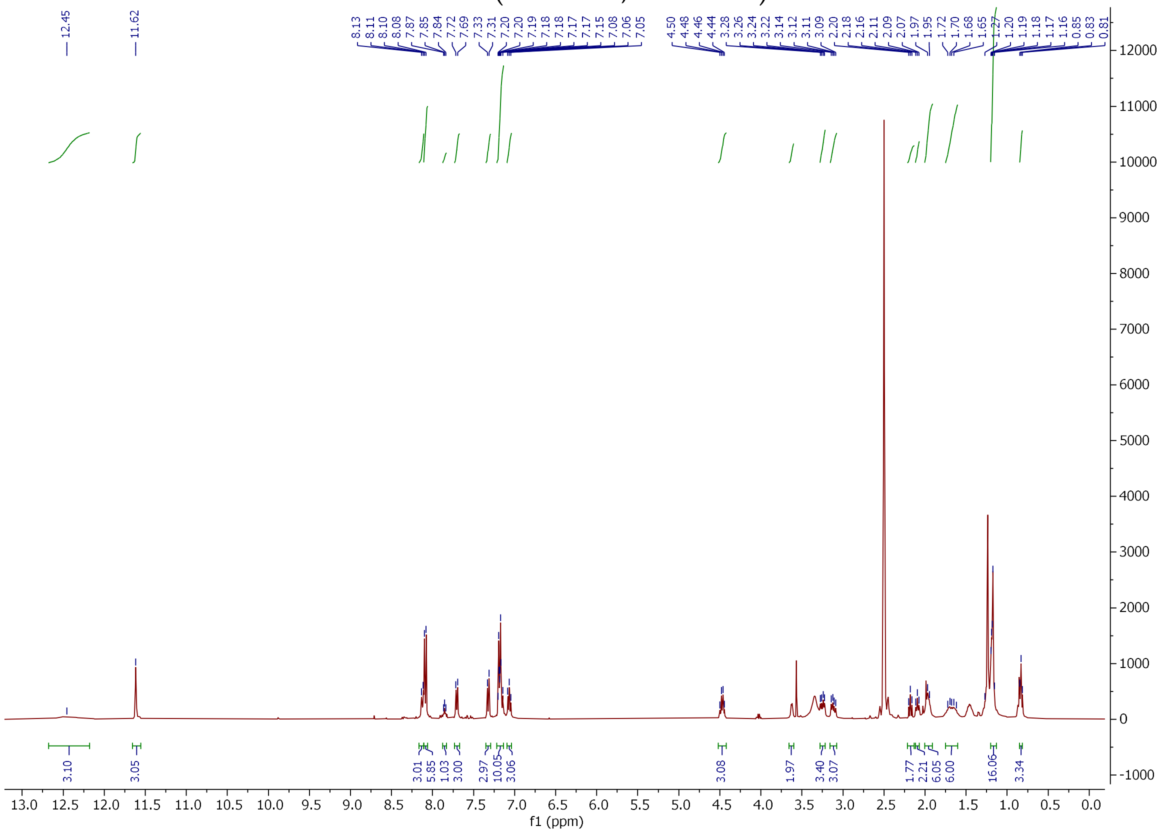

^{13}C NMR (101 MHz, DMSO-d_6)

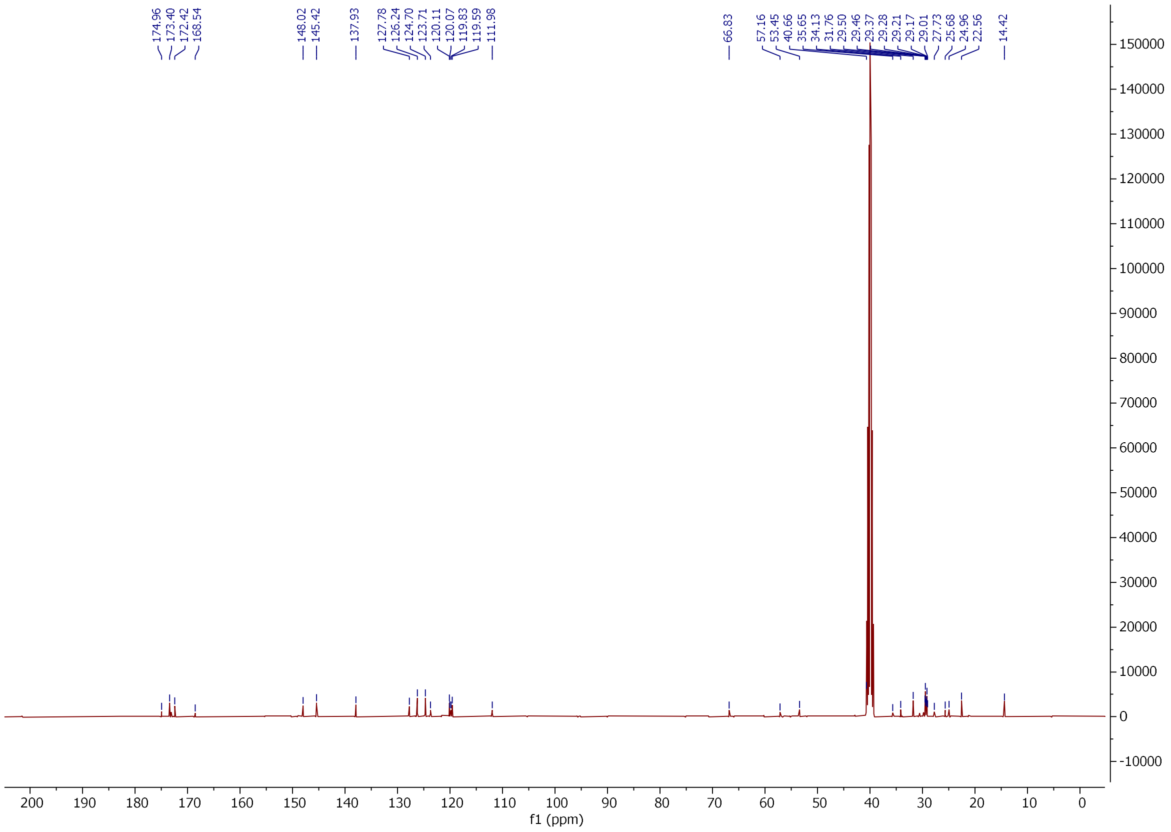

Trimer 61

^{1}H NMR (500 MHz, DMSO-d_6)

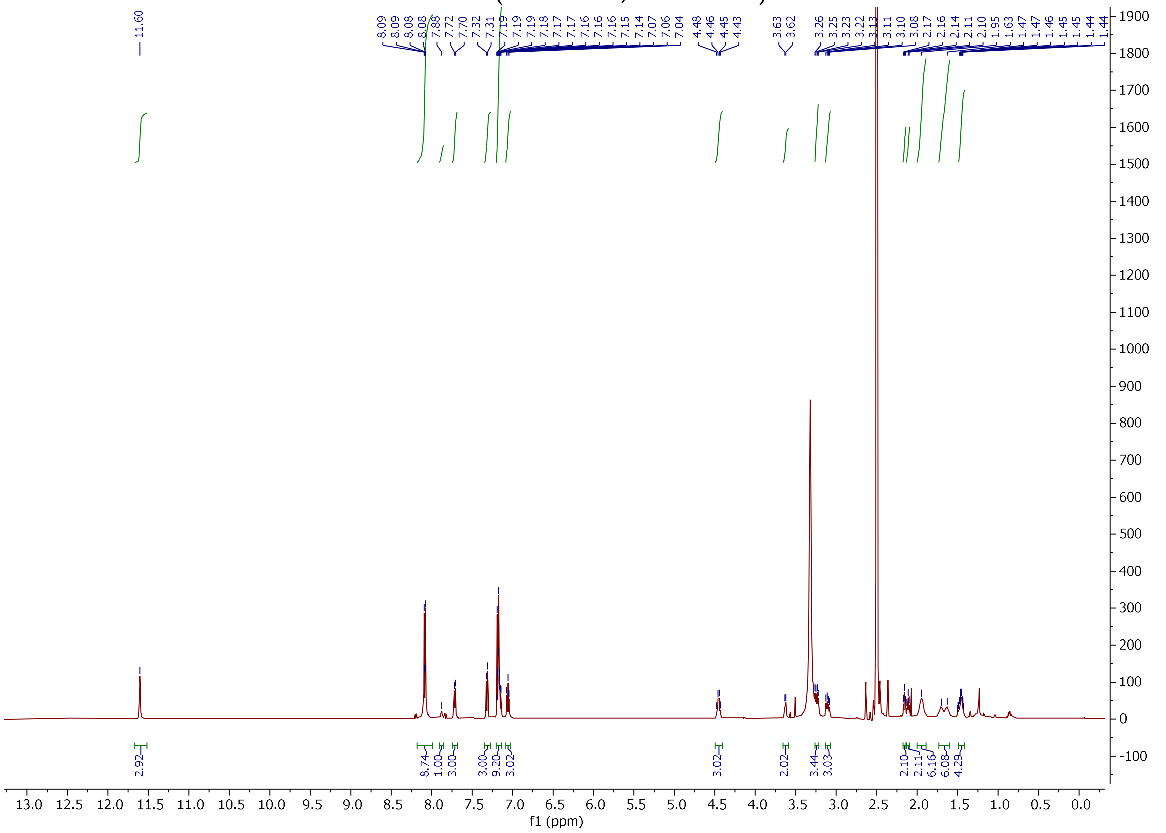

^{13}C NMR (126 MHz, DMSO-d_6)

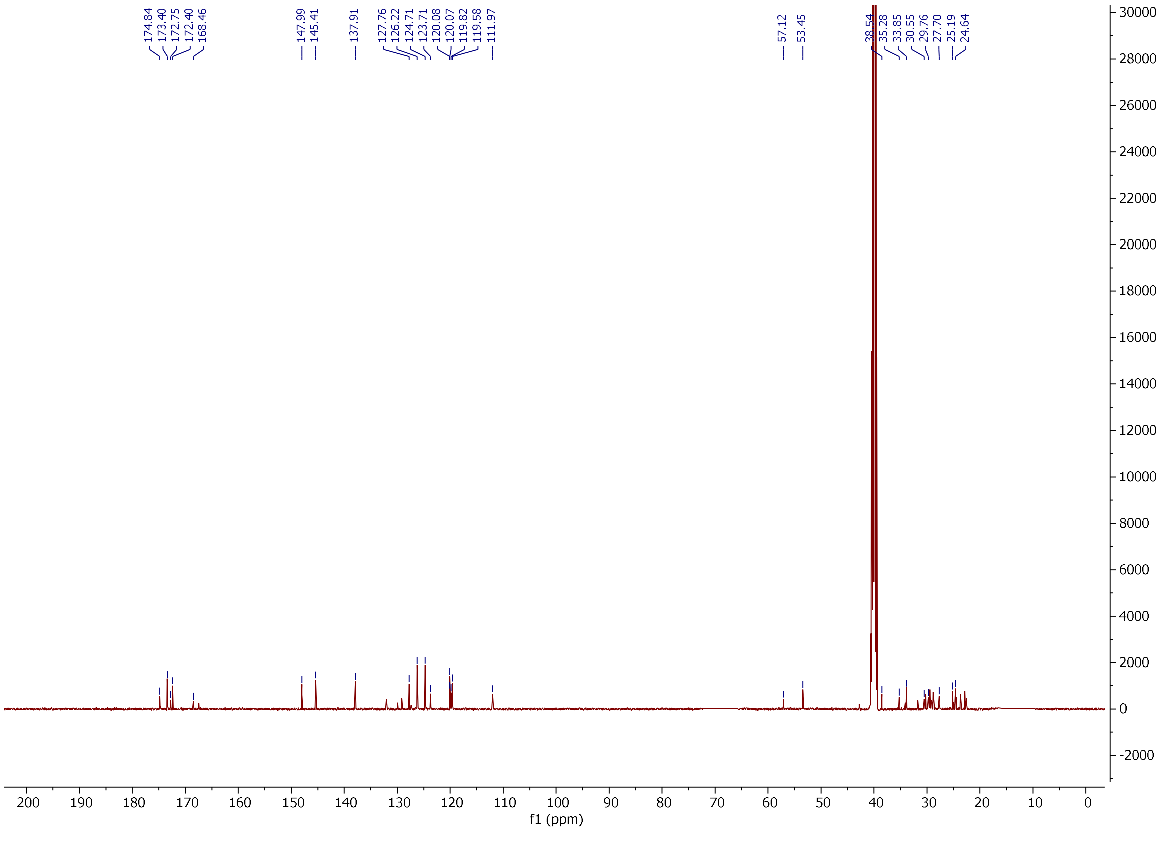

Trimer 63

^{1}H NMR (500 MHz, DMSO-d_6)

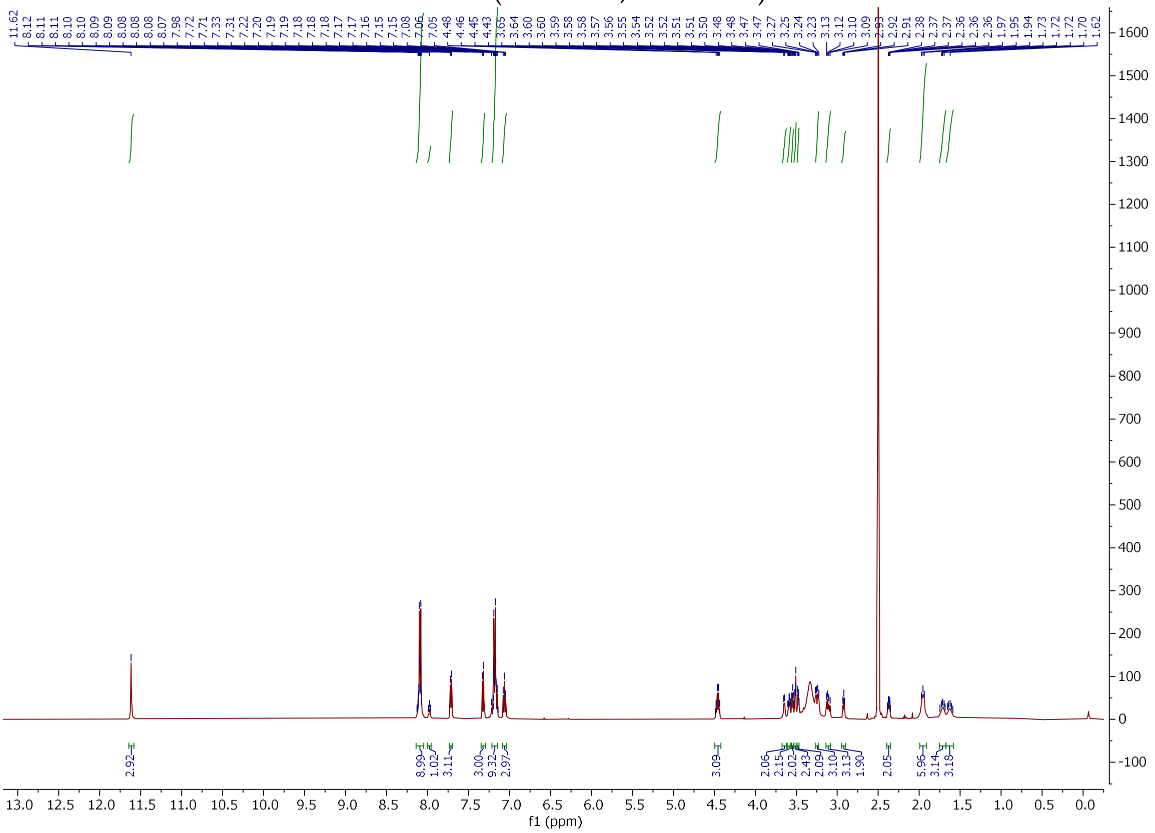

^{13}C NMR (126 MHz, DMSO-d_6)

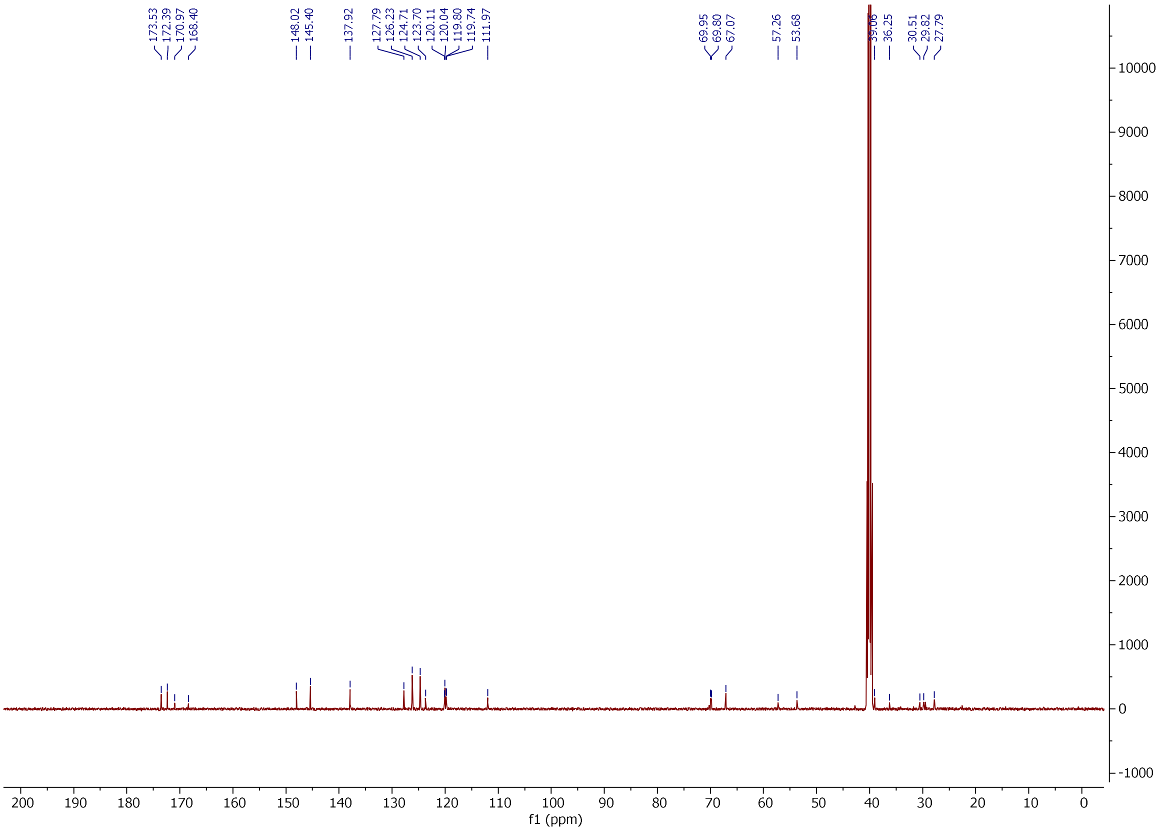

Trimer 65

^{1}H NMR (500 MHz, DMSO-d_6)

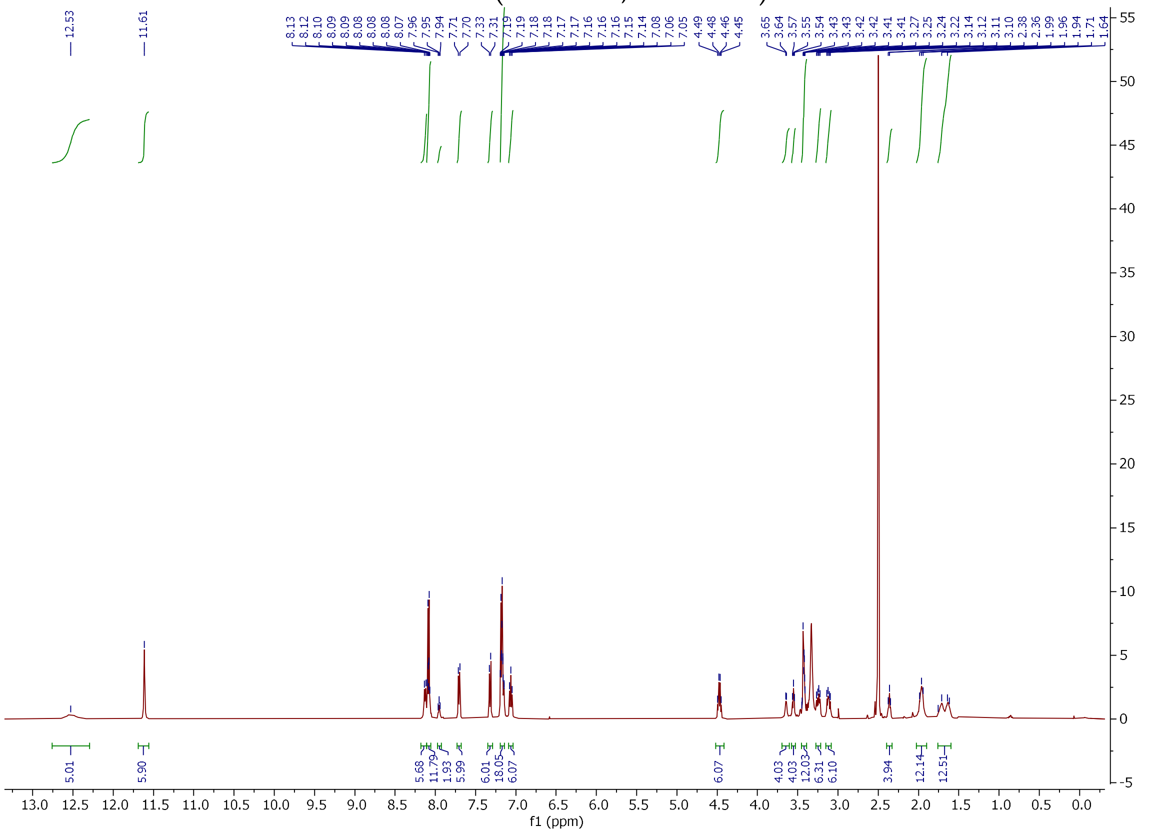

^{13}C NMR (126 MHz, DMSO-d_6)

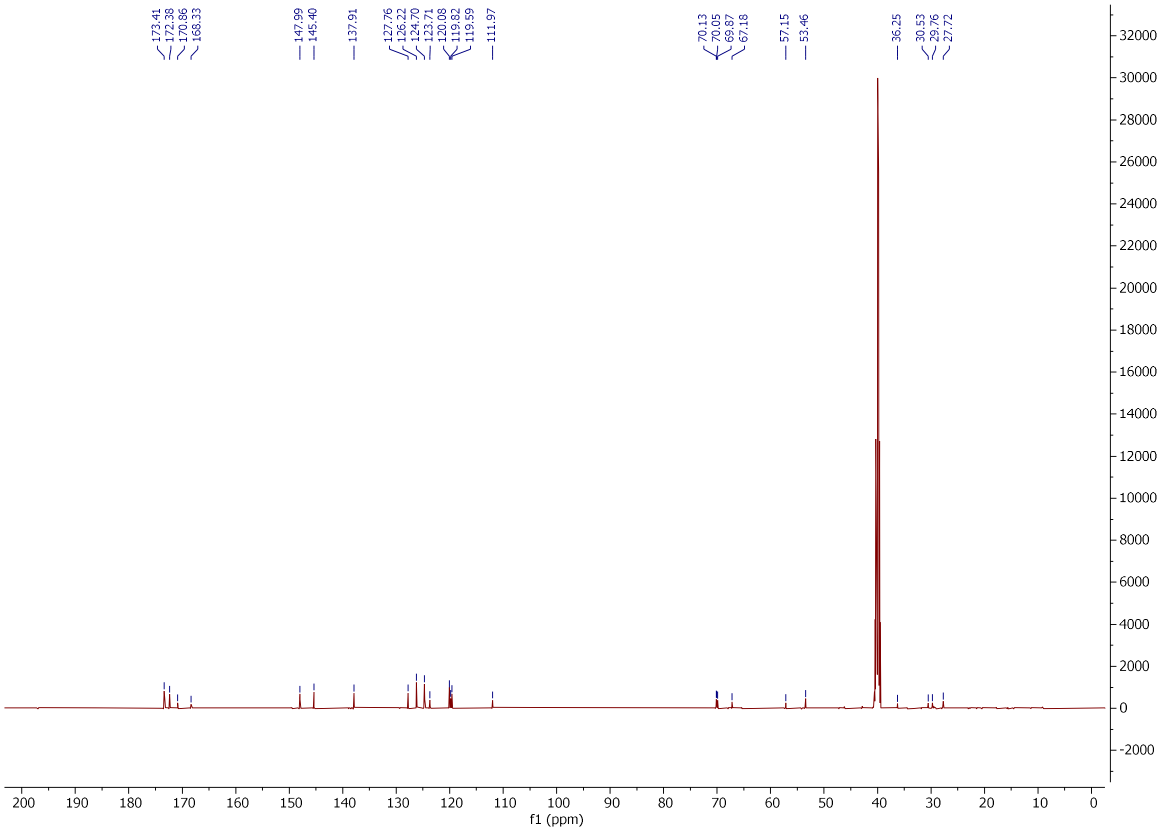

5. HPLC chromatograms of selected compounds

Trimer 2

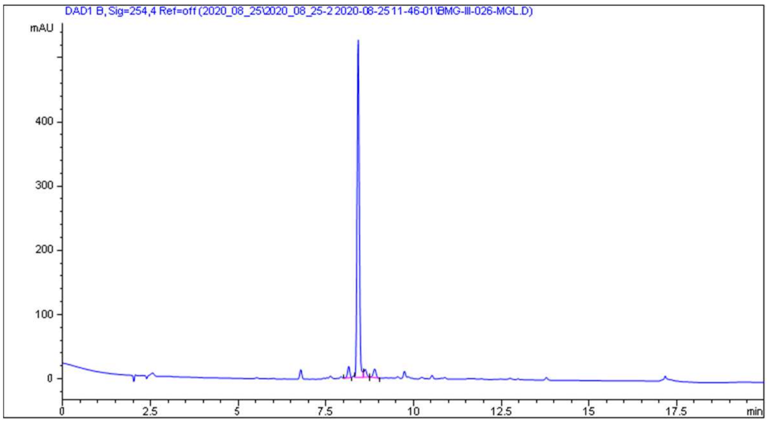

Trimer 36

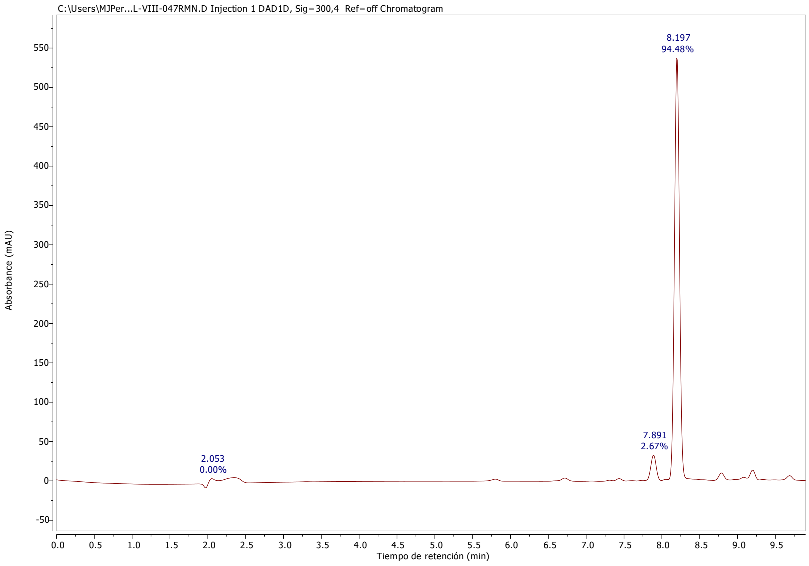

Trimer 37

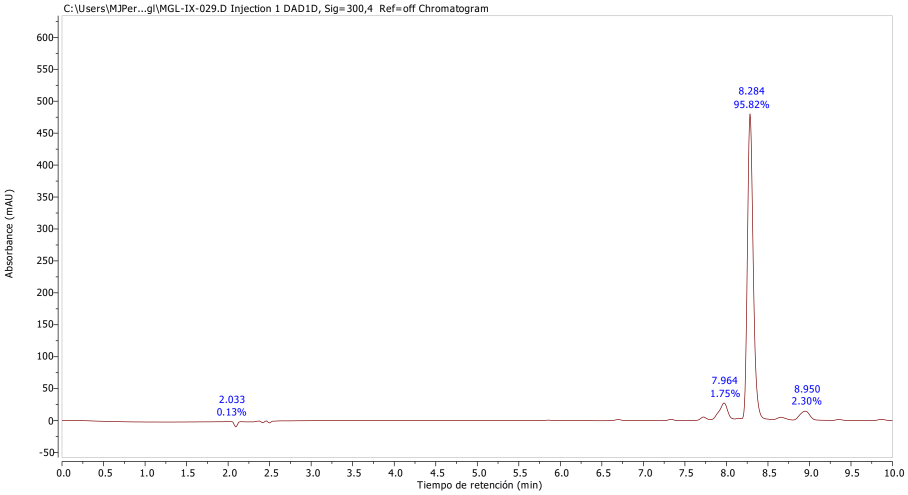

Trimer 39

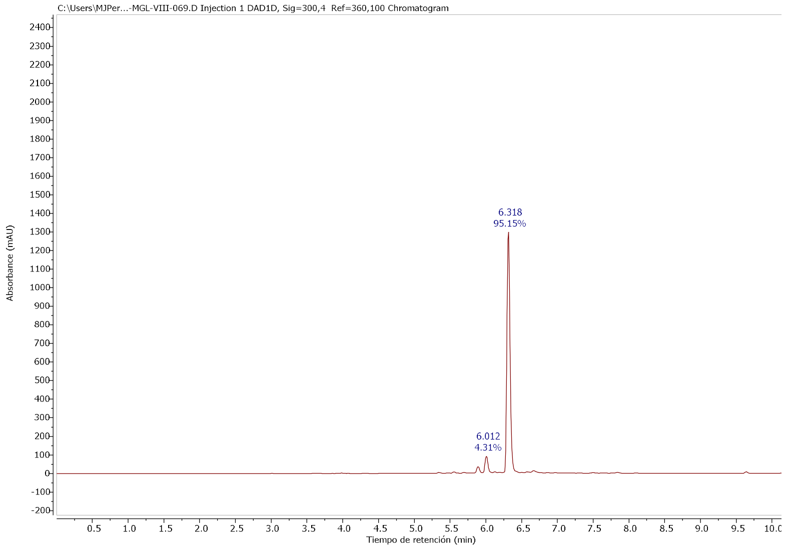

Trimer 41

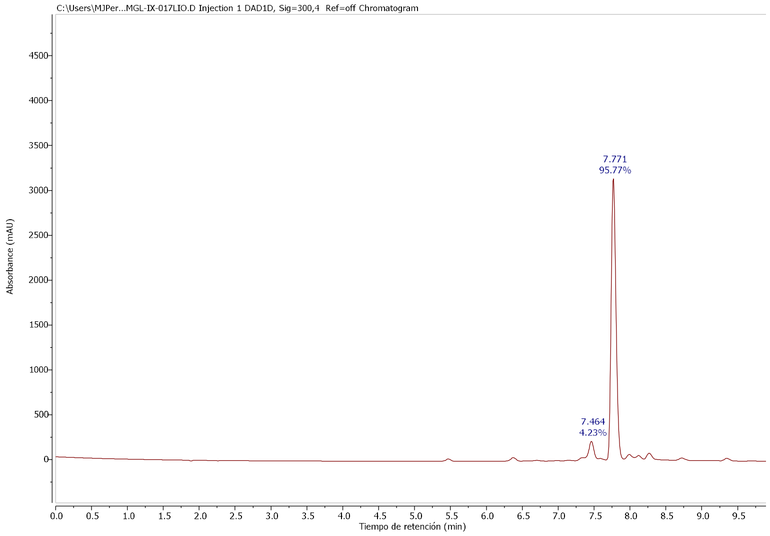

Trimer 42

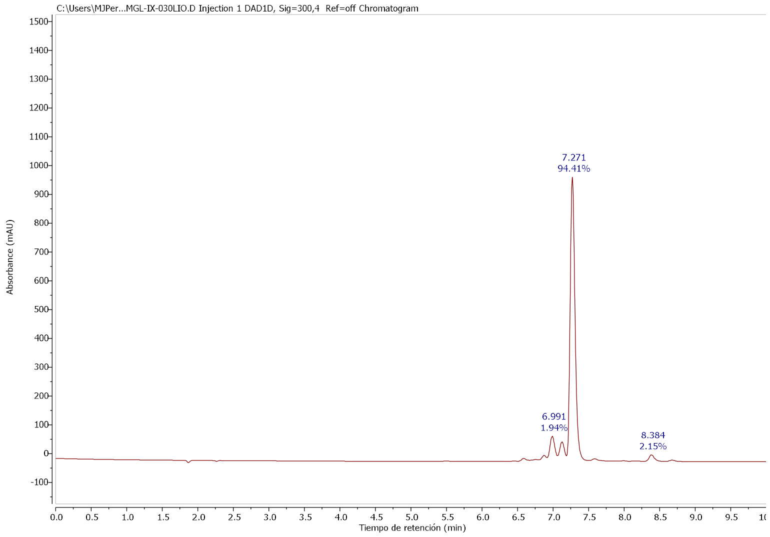

Trimer 46

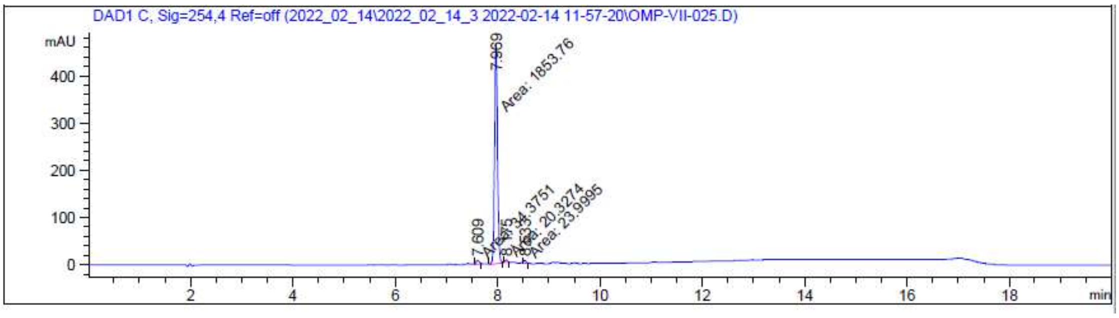

Trimer 63

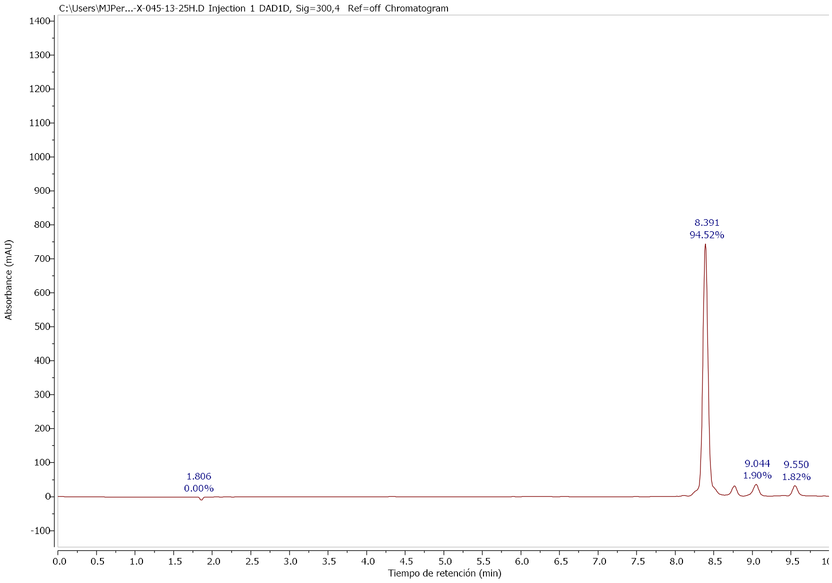

6. References

(1) Habibi, A.; Baghersad, M. H.; Bilabary, M.; Valizadeh, Y. Dithioates of Meldrum's Acid, Dimedone, and Barbituric Acid, Novel Sulfur Transfer Reagents for the One-Pot Copper-Catalyzed Conversion of Aryl Iodides into Diaryl Disulfides. *Tetrahedron Lett.* **2016**, *57* (5), 559–562. https://doi.org/10.1016/j.tetlet.2015.12.085.

(2) Li, Z.; Ke, F.; Deng, H.; Xu, H.; Xiang, H.; Zhou, X. Synthesis of Disulfides and Diselenides by Copper-Catalyzed Coupling Reactions in Water. *Org. Biomol. Chem.* **2013**, *11* (18), 2943–2946. https://doi.org/10.1039/C3OB40464A.
